# Utopia Point Bayesian Optimization Finds Condition-Dependent Selectivity for *N*-Methyl Pyrazole Condensation

**DOI:** 10.1021/jacs.4c01616

**Published:** 2024-05-28

**Authors:** Derek M. Dalton, Richard C. Walroth, Caroline Rouget-Virbel, Kyle A. Mack, F. Dean Toste

**Affiliations:** † Department of Synthetic Molecule Process Chemistry, Genentech, Inc., South San Francisco, California 94080, United States; ‡ Department of Chemistry, University of California, Berkeley, California 94720, United States

## Abstract

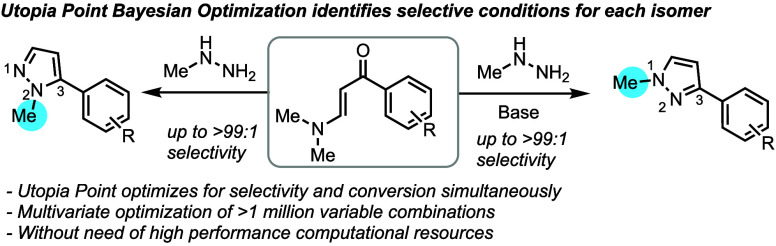

Utopia Point Bayesian Optimization (UPBO) was used to identify reaction conditions that are highly selective for the formation of N1 and N2-methyl-3-aryl pyrazole constitutional isomers. UPBO was used to explore a wide chemical space and identify basic reaction conditions for a typically acid-catalyzed Knorr pyrazole condensation. These studies revealed that selectivity in the reaction stems from a condition-dependent equilibrium of intermediates prior to dehydration. For the N2-methyl isomer reaction pathway, a hemiaminal intermediate was found to form reversibly under the reaction conditions, enabling a highly selective synthesis of the N2 isomer upon dehydrative workup. UPBO was able to successfully optimize conversion and selectivity simultaneously with search spaces of >1 million potential variable combinations without the need for high-performance computational resources.

## Introduction

**Figure 1:**
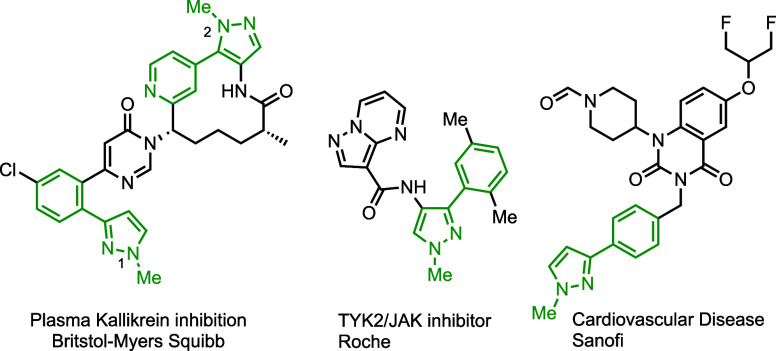
Medicinal drug candidates containing *N*-methyl-3-aryl pyrazole substituents (highlighted).

**Scheme 1:**
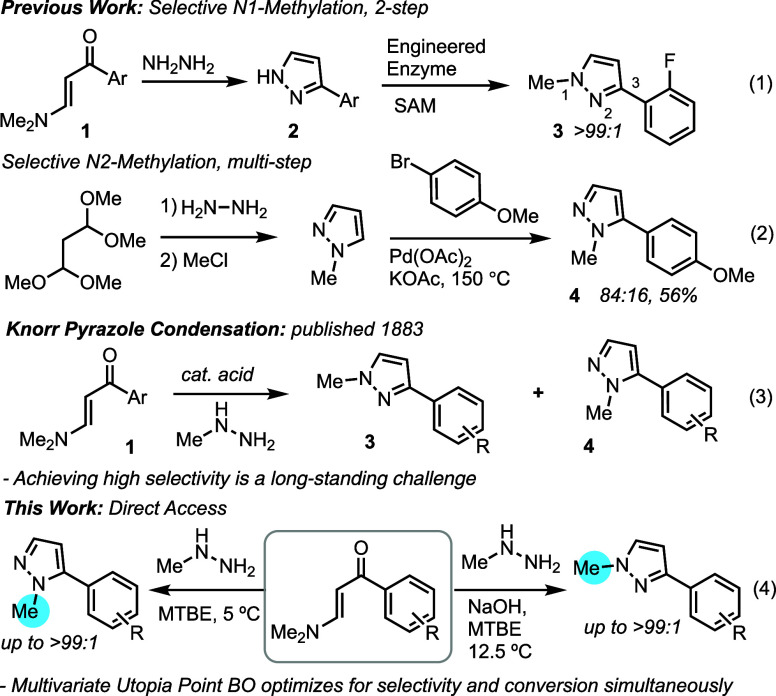
Methodologies to Access N1 and N2-Methyl 3-Aryl Pyrazoles

*N*-Methyl aryl pyrazoles are common motifs in medicinal candidates under evaluation for the treatment of a diverse array of diseases (Figure 1).^1^ The majority of the pyrazoles disclosed are *N*1-methyl constitutional isomers, with only a single candidate containing an N2 isomer.^2^ Identifying reaction conditions that selectively form either the N1 or N2 isomers from a simple starting material would be of benefit to the synthesis of existing drug candidates while enabling the discovery of new drug targets. Herein, we report the use of Utopia Point Bayesian optimization (UPBO) to identify selective conditions to access either the N1 or N2-methyl pyrazole isomers from the same starting material with excellent selectivity (Scheme 1, eq 4). Remarkably, UPBO explored a wide chemical space and identified that basic solvents were optimal in typically acid-catalyzed Knorr pyrazole condensation.

Many methods exist for the synthesis of *N*-methyl pyrazoles.^3^*N*-Methylation of an N–H pyrazole is a common means of forming both N1 (major) and N2 (minor) isomers. Controlling *N*-methylation selectivity can be challenging due to the relatively small size of the methylating reagent. Recently, Hammer and coworkers reported a biocatalytic *N*-methylation of pyrazole that accesses the N1 isomer with high selectivity (eq. 1).^4^ Few methods exist for the selective synthesis of N2 methyl aryl pyrazoles, apart from the cross-coupling of a functionalized N2-methyl pyrazole with an aryl electrophile (eq. 2).^5^*N*-Methyl pyrazoles are also commonly made by Knorr pyrazole condensation, in which an acid catalyst facilitates the condensation of alkyl hydrazine with a 1,3-dicarbonyl equivalent to form a mixture of N1 and N2-methyl pyrazoles (eq. 3).^6^

**Figure 2:**
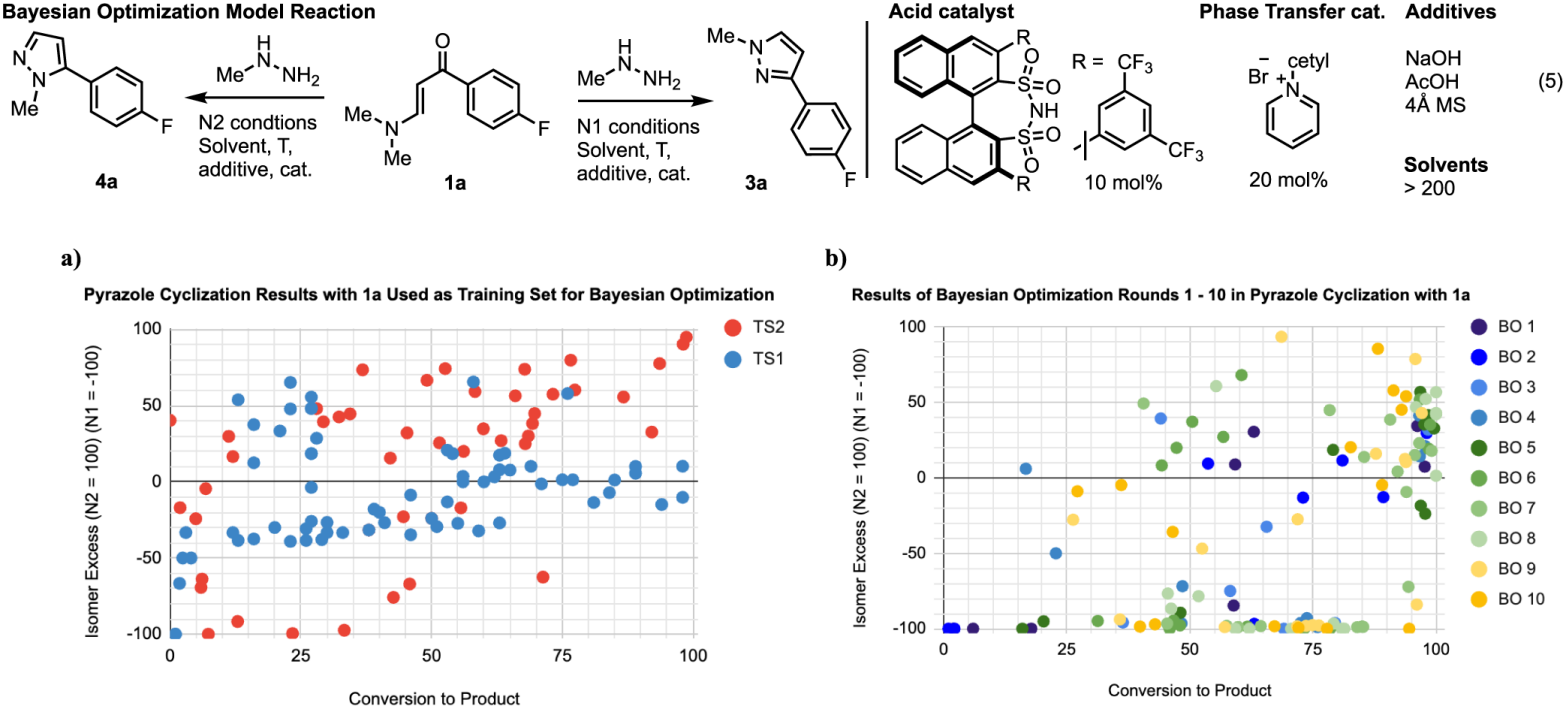
(a) Training data set used for the initial Bayesian optimization. Training set 1 (TS1, blue) shows the results of the initial acid catalysis screen, and training set 2 (TS2, red) shows the results of an expanded reaction scope (see Supporting Information for details). (b) Overlay of Bayesian optimization results for rounds 1–10 (see Supporting Information for details).

On the basis of our previous studies on chiral phosphoric acid (CPA)-catalyzed site selective acylation,^7^ we envisioned that engineered 3,3′-substituted CPA-type catalysts could be tuned to achieve selectivity for either N1 or N2 isomers through secondary noncovalent interactions. A variety of phosphoric acid, *N*-triflyl phosphoramide, and disulfonamide catalysts were tried, and while increases in reactivity were observed, the ability of the catalysts to alter selectivity was moderate (Figure 2a, blue). In order to achieve >90:10 selectivity, we sought to leverage our human-machine partnership^8^ to assess whether a machine learning (ML)-based optimization could identify highly selective conditions for each isomer based on the data already collected.

More specifically, could ML identify highly selective conditions for both N1 and N2-methyl pyrazole formation for the Knorr pyrazole condensation?^9^ Most of the Bayesian Optimization (BO) literature published thus far has been retrospective, meaning that data sets have already been collected.^10^ In the present work, UPBO was used to predict conditions in a forward sense, thus, required fine-tuning in response to the performance of the algorithm in real time.^11^ Control of N1 and N2 selectivity in Knorr pyrazole condensation is a long-standing problem and represents a great opportunity to evaluate the utility of the BO.

BO uses surrogate functional models to fit empirical observations. Crucially, BO relies on models that can provide a measure of the uncertainty around the predicted value. Most often, the surrogate function will be a Gaussian process regressor, though other models such as Bayesian neural networks or random forests can also be employed. As the individual reactions took considerable time to run and analyze, multiple models were employed in order to generate multiple reaction conditions to try in each round (see SI).

Balancing exploring areas of high uncertainty or seeking areas where the model predicts greater success is accomplished by using an acquisition function. Multiple functions have been identified for this task. In this work, two acquisition functions were used for each of the functional models: one was tuned to be more exploratory, prioritizing areas of higher uncertainty, while the other was tuned to be more exploitative, favoring areas where the model predicted improvement in the target metric. Upon the conclusion of the optimization runs, a clearly superior model and acquisition function combination did not emerge. The relative rankings of the models varied in a random fashion, round over round (see Supporting Information).

## Results and Discussion

The Knorr condensation reaction was first optimized by traditional human-guided means (Figure 2, eq 5). This initial optimization led to a preliminary data set to seed the first round of BO. In our experience, this is a more typical starting point for optimization, where a chemist has already tried several standard conditions before turning to BO as a way to get over an apparent reactivity or selectivity limit. This initial training set was augmented with an expanded solvent screen that showed that MTBE provided a moderate improvement in selectivity for the N2 isomer (Figure 2a, red). Additionally, it was found that increasing amounts (5, 10, 20 equiv) of methylhydrazine in MTBE fortuitously provided the N2 isomer with 98% conversion and 97:3 N2/N1 selectivity, albeit with 20 equiv of methylhydrazine. Phase transfer conditions were also explored and found to provide excellent selectivity (1:99) for the N1 isomer; however, poor conversion (33%) (Figure 2a, red).

For the initial experimental design of the BO, the amount of methylhydrazine was limited to 5 equiv, as high excesses of methylhydrazine can be hazardous at the scales found in process chemistry settings (Figure 2b). Limiting the amount of methylhydrazine eliminated the top 3 results for the N2-selective condensation. A single acid catalyst (disulfonamide, DSI2, 10 mol %), and a phase transfer catalyst (cetylpyridinium bromide, CPB, 20 mol %) were included. Each was best performing in our initial experiments. Three additives were included: acetic acid, sodium hydroxide (50% aqueous), and 4 Å molecular sieves. The equivalents of acid and base were combined into a single numeric parameter, with excess acid equivalents represented by positive values and base equivalents by negative values. The temperature range was limited to 0 to 50 °C. Solvent volumes were restricted to 10–30 volumes (mL/g of reactant). More than 200 solvents were parametrized in Cosmotherm and made available for the model’s use.^12^ Overall, there were three Boolean parameters (use of DSI, use of CPB, and use of sieves), four numerical parameters (equivalents of methylhydrazine, temperature, equivalents of acid or base, and solvent volume), and four numerical parameters for the solvent.

**Figure 3:**
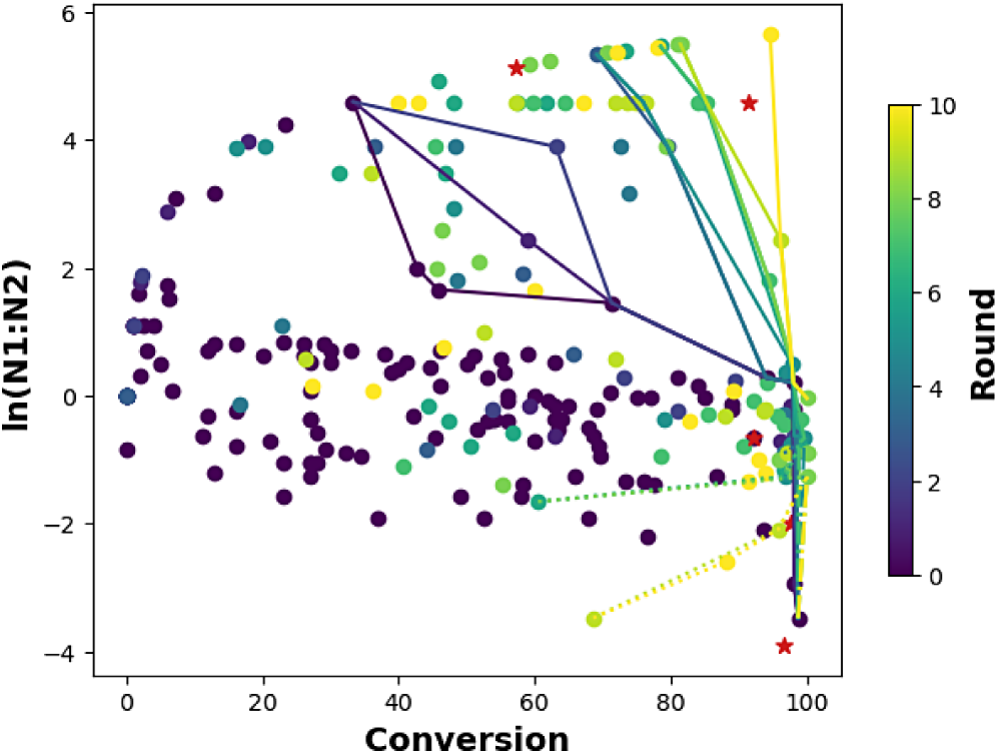
Plot of the results for 10 rounds of Bayesian optimization. Points are color coded by round. Lines represent Pareto front for total data set up to that round (solid for N1 selective, dashed for N2 selective, not including preliminary data). Red stars represent results for the predicted best conditions.

For the first 4 rounds of BO, conversion and selectivity were modeled individually and each subjected to an acquisition function (see Table 1 and Figure 2b). The values of the acquisition function were then pareto sorted to find points that offered the best trade-off between yield and selectivity (Figure 3). The process was started with five reaction conditions that were predictive for each isomer (10 reactions total). The conditions selected in this manner only resulted in modest improvements. Moreover, some of the best conditions suggested employed atypical solvents, such as *p*-cymene (round 1), diethylamine (round 2), and tributylamine (round 4). In general, many conditions were identified that provide high selectivity for the N1 isomer (99:1 N1/N2, 76%) with moderate conversion, but accessing the N2 isomer with high selectivity was challenging. While several conditions provided moderate selectivity for the N2 isomer with great conversion (71:29, N2/N1, 87%), none provided the desired high selectivity (>90:10).

**Table 1:** Highest N1 and N2 Selectivity and Conversion Results from Each Round of Bayesian Optimization

#	model	AcOH (equiv)	NaOH (equiv)	MeNHNH_2_ (equiv)	DSI2 (1/0)	PTC (1/0)	MS (1/0)	temp (°C)	vol (mL/g)	solvent	N2	N1	prod (%)
BO1	GPR_ucb_2_n2	5	0	5	1	1	1	50	30	*p*-Cymene	70	30	98
BO1	RFR_ucb_2_n2	0	5	2	1	0	1	50	30	2-MeTHF	8	92	59
BO2	MLP_ei_05_n1	3	0	5	1	1	1	50	10	MeCN	65	35	98
BO2	RFR_ucb_2_n2	0	5	5	0	0	1	25	10	diethylamine	2	98	63
BO3	MLP_ucb_2_n1	5	0	5	0	0	0	0	10	isoamyl alcohol	66	34	98
BO3	RFR_ei_05_n1	0	2.8	5	0	1	0	25	10	MTBE	2	98	80
BO4	GPR_ei_05_n1	5	0	3	0	1	0	25	10	cyclohexane	71	29	97
BO4	RFR_ucb_2_n2	0	2.8	5	0	1	0	12.5	10	tributylamine	1	99	76
BO5	MLP_ucb_2_n1	5	0	5	1	1	0	12.5	10	*m*-cresol	78	22	97
BO5	GPR_ucb_2_n2	0	1.7	5	0	0	0	25	10	triethylamine	5	95	48
BO6	RFR_ei_05_n1	0	0.6	5	0	1	0	12.5	10	MTBE	84	16	61
BO6	Grad_ei_05_n2	0	5	5	1	1	1	12.5	23	oleyl alcohol	1	99	85
BO7	RFR_ei_05_n1	0.6	0	5	0	0	0	0	10	MTBE	72	28	78
BO7	Grad_ei_05_n2	0	5	5	1	0	1	12.5	30	tributylamine	1	99	85
BO8	RFR_ucb_2_n1	5	0	5	1	1	0	12.5	8	*m*-cresol	78	22	100
BO8	Grad_ucb_2_n2	0	5	4	1	0	1	12.5	30	oleyl alcohol	1	99	81
BO9	GPR_ei_05_n1	0.6	0	16	0	0	0	0	23	*n*-propylamine	97	3	69
BO9	MLP_ucb_2_n2	0	5	20	1	0	0	50	30	tributylamine	8	92	96
B10	GPR_ei_05_n1	0.6	0	20	0	1	0	0	10	pyrrolidine	93	7	88
B10	MLP_ei_05_n2	0	5	13	1	0	0	0	10	tributylamine	1	99	95
P1	N2_pred_best	0	0.5	14	1	0	0	0	10	tributylamine	90	10	99
P1	N1_pred_best	0	5	20	0	0	0	12.5	30	MTBE	1	99	86

**Figure 4:**
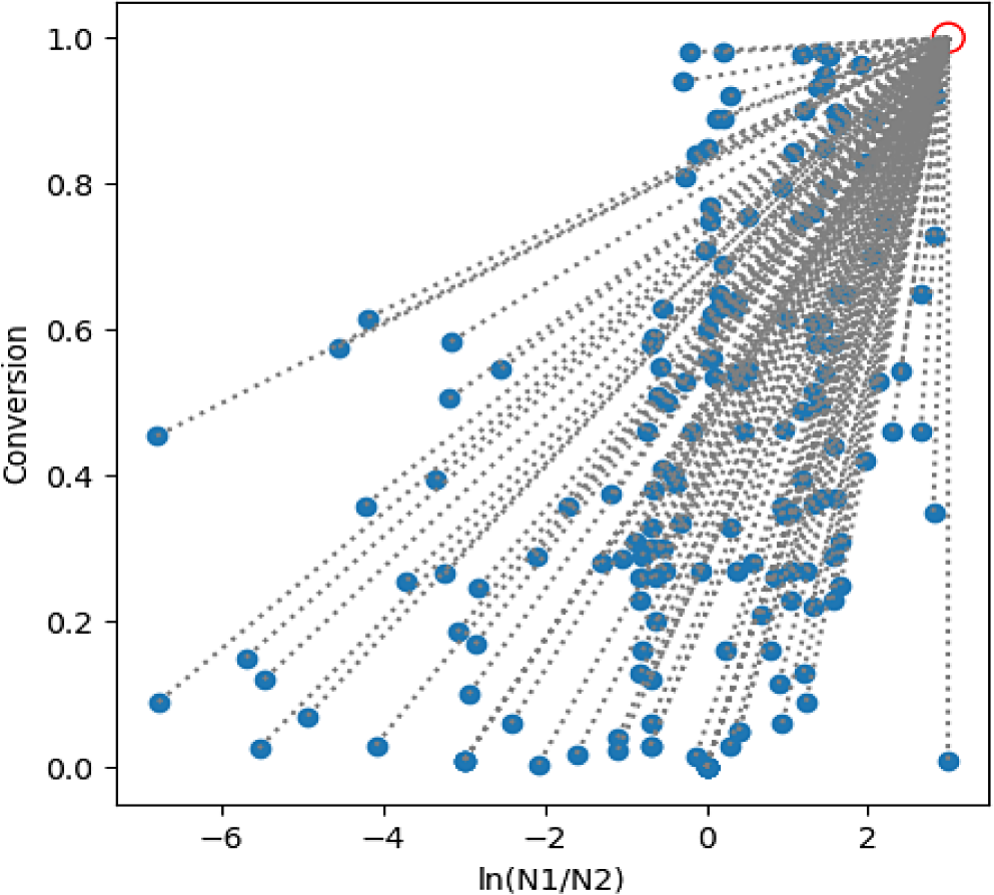
Plot of Utopia Point (red circle) for N2 isomer multiobjective optimization.

At this point, BO strategies were changed in order to find a better method to balance yield and selectivity optimization. Multiple options have been previously reported for multiobjective optimization, including multidimensional algorithms, such as expected hypervolume improvement and scalarization algorithms, which convert multiple objectives into a single objective problem. Utopia point-based optimization was selected, as it is mathematically simple to implement the scalarization method and allows for simultaneous optimization of multiple objectives (Figure 4).^13^ In this method, an unreachable Utopia point is selected, and all data points are measured against it. Instead of individual components being modeled, the distance to the unreachable Utopia point is modeled. The point is selected to be unreachable because if any outcome were to exceed the Utopia point, it would be perceived by the model as less than optimal. The placement of the Utopia point in 2-D space can be used to weight the optimization. For example, to weight selectivity over conversion, a point is selected that is further along the selectivity axis. To weight conversion, a Utopia point is selected that is further along the conversion axis. Two Utopia points (one for each N1 and N2) were created for this optimization and were weighted equally for selectivity and conversion. This method has two advantages over other methods. First, as a simple Euclidean distance, it is intuitive to understand for nonexperts in ML. Also, it is highly scalable to larger search spaces as it is not as memory-intensive as other multiobjective algorithms that rely on numerical methods.^14^ Search spaces were generated with over 1 million potential combinations of conditions and solvents, which were calculated without the need for high-performance computer resources. As most synthetic chemists need to optimize both selectivity and reactivity simultaneously, this method is likely to find great interest in the general community.

After implementing the Utopia point method, small gains in selectivity for the N2 isomer (Figure 6) and significant increases in the conversion for the N1 isomer were observed (Figure 4). The number of reactions for each isomer was increased to 8 (16 total) per round. After an additional 2 rounds of optimization (8 total rounds), conditions leading to improvement in the N1 conversion while maintaining high selectivity were identified (99:1 N1/N2, 85% conversion) without human intervention. In contrast, only a modest increase in selectivity for the N2 isomer was observed (BO6, 84:16 N2/N1, 61% conv). Previous experiments suggested that the equivalency of methylhydrazine affected selectivity; thus, we intervened and relaxed the enforced limit of hydrazine equivalents from 5 to 20.

**Figure 5:**
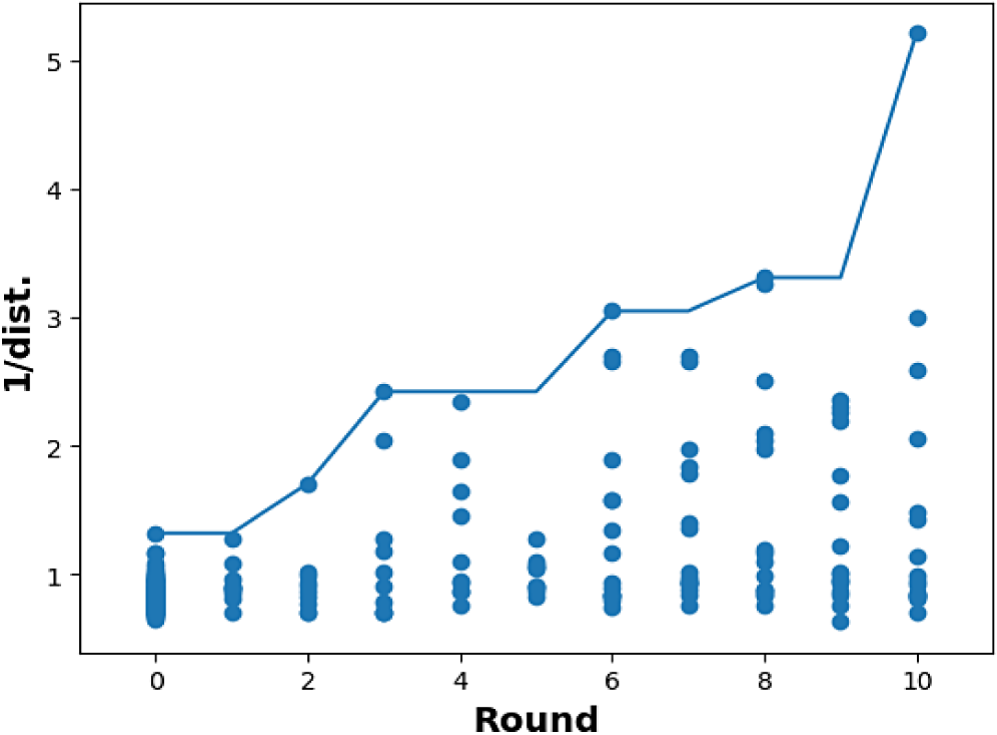
Plot of inverse distance to Utopia point for N1 selective cyclization vs Bayesian optimization round. Lines represent running maxima at each round.

**Figure 6:**
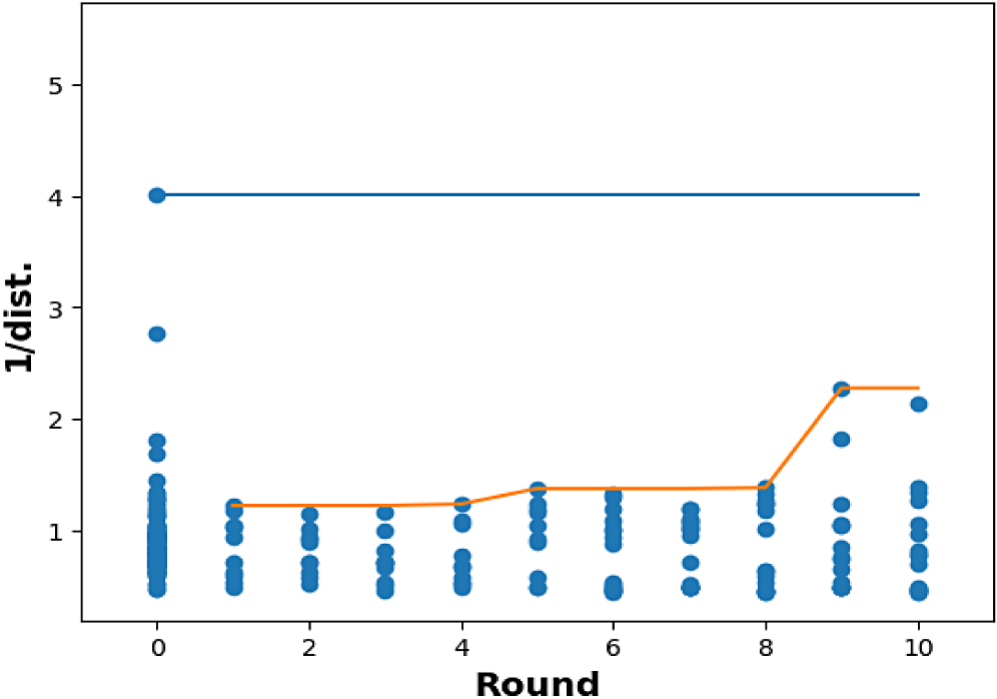
Plot of inverse distance to Utopia point for N2 selective cyclization vs Bayesian optimization round. Lines represent running maxima at each round.

Fortunately, allowing the models to increase the methylhydrazine equivalents led to an increase in selectivity for the N2 isomer. After the ninth round of BO, unique conditions were identified that met the aims of achieving >90:10 selectivity for both the N1 and N2 isomers. For the N2 isomer, conditions were generated that provided 97:3 N2/N1 selectivity with a conversion of 69%; hoping to improve this, a 10th round of optimization was completed. The 10th round identified excellent conditions for both N2 and N1 isomers, affording 93:7 N2/N1 with 88% conversion and 100:0 N1/N2 with 95% conversion. It is remarkable that the solvents selected were pyrrolidine (N2 selective) and tributylamine (N1 selective). These are both basic solvents, which provide a significantly different environment from the typical acid-catalyzed Knorr pyrazole reaction conditions (in DCM) that were employed at the outset of this study.

At this point, we felt the data set was large enough to try for a predictive model instead of one that would leverage BO methods. A random forest regressor was identified as having the best fitting statistics (RMSE = 0.03 and 0.05 for the 1/dist. prediction for the N1 and N2 isomers, respectively) and was used to predict conditions that would give the shortest distance to the Utopia point. The identified conditions were found to indeed be among the best-performing reaction conditions, validating the predictive power of the model.

Ultimately, the optimization of the formation of the N1 isomer required minimal intervention on the part of the chemist to identify unique, highly selective conditions. and it serves as a valuable case study for ground-up, closed-loop BO optimization. On the other hand, the optimization of the N2 isomer may not have achieved the desired selectivity aim without the intervention of the chemist. Overall, we concluded that Bayesian optimization is a valuable tool to the chemist as it can remove bias during optimization, but the insight of an expert can also help to direct the BO toward optimal outcomes.

In this study, we implemented UPBO midway through the optimization when a large data set was already available. We find that in practice there is usually a large amount of preliminary data when optimizing a reaction, but to compare with the rest of the literature, we also ran synthetic benchmarks from random starting points. The UPBO algorithm converged to the optimal solutions on average in 8 rounds or less for both isomers when using the experimental data set as a search space. In contrast, random selection of data points could not find the optimum conditions on average (see Supporting Information Figure S7). In order to test on the largest possible search space, we used an average of the Gaussian process, random forest, and neural network predictors to predict all values for our larger search space and again ran multiple rounds of UPBO from random starting points. While the algorithm did not always reach the global maximum, it did manage to show round-over-round improvement. We also ran a feature importance test to determine whether the solvent parameters were indeed necessary for the models. We found that for selectivity and conversion, the collected solvent parameters were second only to the amount of acid or base added in terms of feature importance (Figure S9).

**Figure 7:**
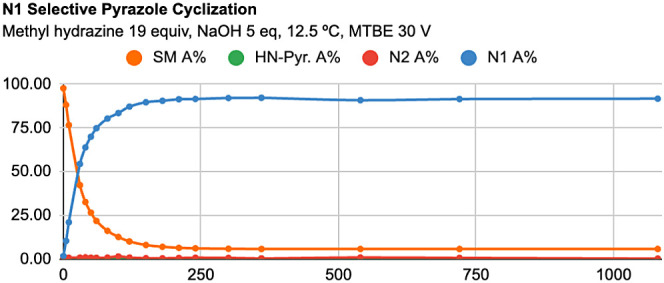
Reaction profile of N1-selective condensation with 1a.

**Figure 8:**
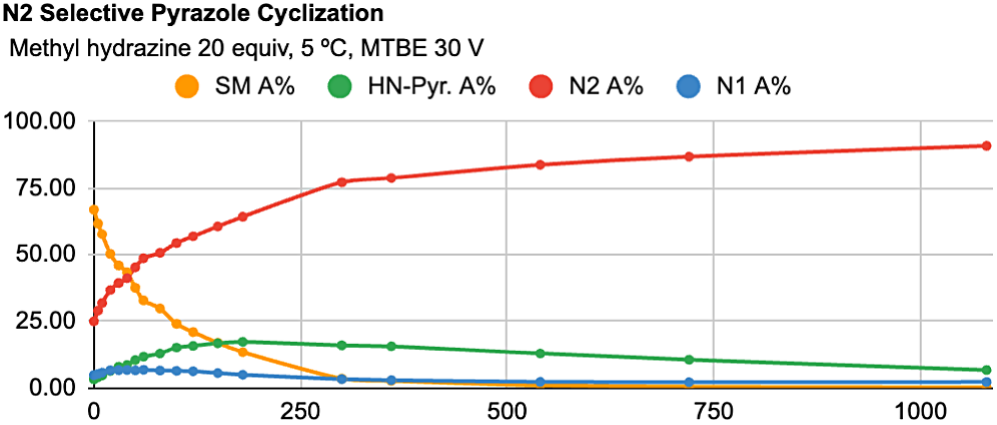
Reaction profile of N2-selective condensation with 1a.

Reaction profiles for the N1 and N2 selective pyrazole condensations were determined via HPLC analysis. The N1 selective pyrazole condensation showed complete consumption of the starting material within 210 min with a 99:1 (N1:N2) product ratio, 92% product formation, and a clean reaction profile (Figure 7). For the N2-selective condensation, starting material consumption was completed at ∼300 min; however, N2 product formation continued to increase up until 1080 min. Additionally, the formation of the N–H pyrazole increased early in the reaction and then decreased over time. Finally, the ratio of N2/N1 product increased from 84:16 initially to 98:2, with a 93% conversion at 1080 min (Figure 8).

**Scheme 2:**
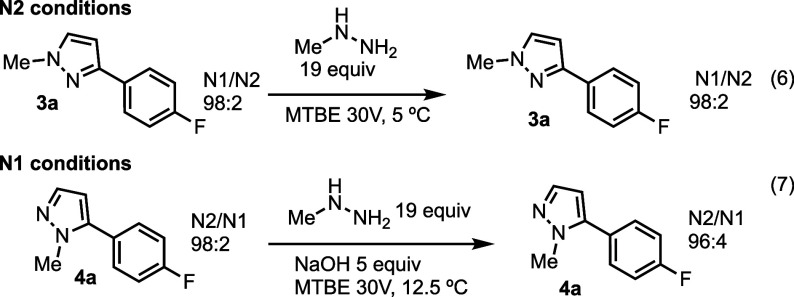
Control Reactions With N1 Isomer **3a** Subjected to N2 Conditions (Top) and N2 Isomer **4a** Subjected to N1 Conditions (Bottom)

Subjection of the N1 product **3a** to the N2 condensation conditions had no apparent effect on the product ratio, and N1 product **3a** was observed (Scheme 2, eq. 6). However, when 50% aq sodium hydroxide was added to the reaction mixture after observing complete N2 product **4a** formation by HPLC, the N2 product was readily converted to the N1 product (see Supporting Information for details). Interestingly, this reversibility was not observed with chromatographed N2 product **4a** subjected to the same conditions (Scheme 2, eq 7).

**Scheme 3:**
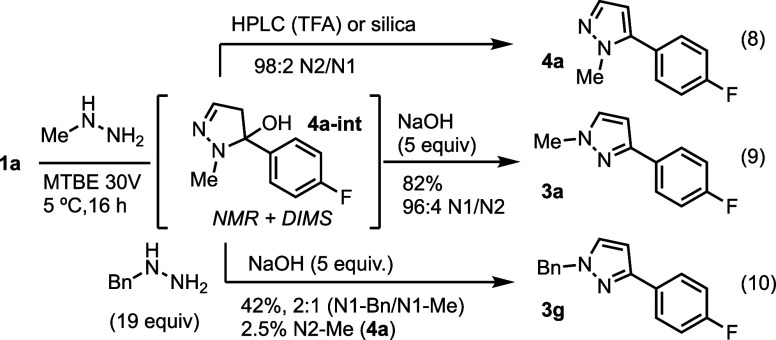
Control Reactions With N2 Intermediate **4a-int** Subjected to Acidic, Basic, and Alternative Hydrazine Conditions

A combination of ^1^H- and^19^F-NMR and direct injection mass spectroscopic analyses revealed that after 16 h under these conditions, the hemiaminal **4a-int** was present (Scheme 3). This intermediate rapidly dehydrates in the presence of acid, such as aqueous TFA (HPLC) or silica gel during chromatography, to give the N2-methyl pyrazole isomer almost exclusively (98:2 N2/N1, eq. 8). However, if the **4a** intermediate was subjected to aq. NaOH, the N1-methyl pyrazole isomer **3a** was formed again with very high selectivity (eq 9., 96:4 N1/N2). Furthermore, when benzylhydrazine and NaOH were added to a solution containing **4a-int**, the N2-methyl pyrazole intermediate was converted into N1-pyrazole products in 42% yield as a 2:1 ratio of N1-benzyl and N1-methyl pyrazoles with 2.5% of N2-methyl pyrazole remaining (eq. 10). This reversibility has not, to the best of our knowledge, been previously reported for pyrazole intermediates.

**Scheme 4:**
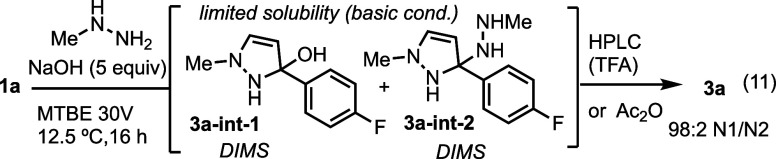
Control Reactions With N1 Intermediates **3a-int-1,-2** Subjected to Acidic or Dehydrating Conditions

The fact that the hemiaminal was observed under relatively neutral conditions prompted us to further evaluate the conditions that favored N1 formation. A similar evaluation of the N1 selective reaction mixture was complicated by the fact that the reaction is biphasic; a residue forms upon the addition of the NaOH solution. DIMS and NMR analysis identified two intermediates, hemiaminal **3a-int-1** and aminal **3a-int-2**, each was able to form N1-methyl pyrazole **3a** (Scheme 4, eq. 11). The high selectivity observed in the presence of NaOH may be due to the poor solubility of the intermediates in MTBE, which are sequestered from the reaction medium. In contrast to the N2 selective reaction profile, the N1 selective reaction profile does not show a change in selectivity over time, suggesting that there is little equilibration. The N1 intermediates form quickly and do not appear to equilibrate in the way that the N2 intermediates do.

**Figure 9:**
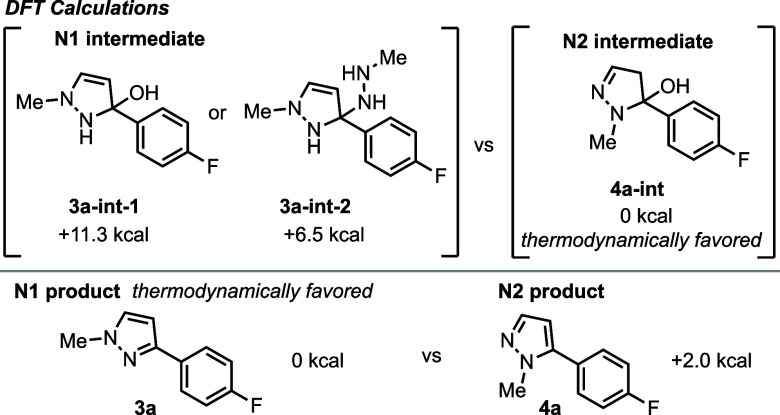
DFT calculations of 1a intermediates and products.

DFT calculations were performed to determine the relative energies of the intermediates and products of the N1 and N2 *N*-methyl pyrazoles **3a** and **4a** (Figure 9, see Supporting Information for details). The calculations show that while the N1 product **3a** is 2.0 kcal/mol lower in energy and thus thermodynamically favored over the N2 product **4a** (in THF), the relative energies of the intermediates reveal a different story. Because both reactions are run under basic conditions at low temperatures, the dehydration event is slow, and the hemiaminal or aminal intermediates are present until workup. As such, selectivity is determined by the relative ratios of the intermediates as opposed to the pyrazole products. DFT calculations show that the hemiaminal **4a-int** enroute to the N2 product is ∼7 kcal lower in energy than the **3a** intermediates. Under reversible reaction conditions, such as N2 conditions, **4a-int** is favored thermodynamically. The N2 reaction profile supports this, as the ratio of N2/N1 increases over 16 h to favor the N2 intermediate (Figure 8). In the presence of NaOH, there is an apparent reversal in the direction of the equilibrium favoring the N1 intermediates. This is not fully understood but may be derived from their poor solubility relative to the N2 intermediates. In short, the UPBO optimization has led us to conditions that favor the respective N1 and N2 intermediates and, in so doing, increased the selectivity for both product formations. Thus, the thermodynamically favored N1 product forms not by equilibration but through the trapping of a nonthermodynamic intermediate. While the higher energy N2 product is formed selectively through equilibration to a thermodynamically favored intermediate.

These conditions were expanded to substrates that are similar in structure to the optimized 4-F-aryl vinylogous amide **1a**, such as phenyl **1b** and meta-chlorophenyl **1d** substrates (Table 2). A heteroaromatic compound, such as 3-pyridyl **1c**, was also well tolerated. Electron-donating substituents 4-methoxyphenyl **1f** performed well, although a small amount of NH-pyrazole **2f** was observed with both condensation conditions, affecting the assay yield. Ortho-substituents are also well tolerated under the conditions that afford both N1 and N2 products.

**Table 2:**
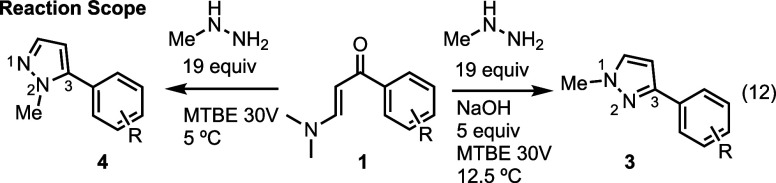

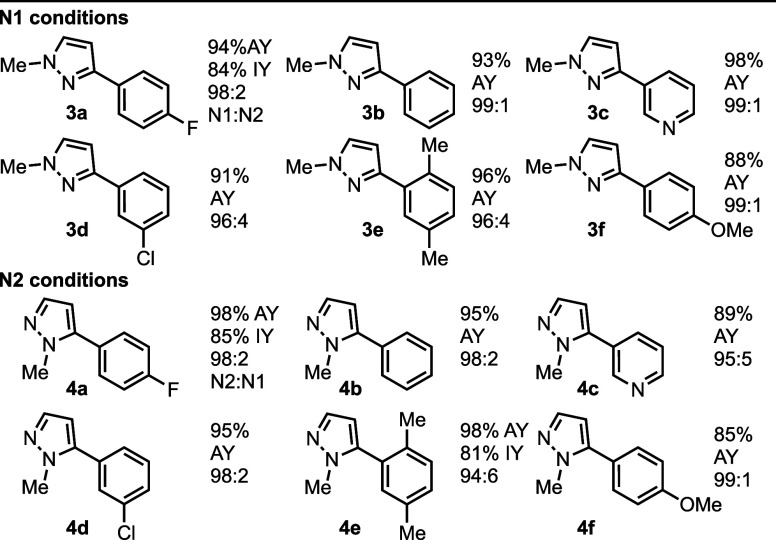
Reaction Scope for N1- and N2-Selective Pyrazole Condensation Conditions^a^

a Yields are reported as assay yield determined by HPLC with respect to an internal standard. Isomer ratio determined by HPLC and 1H-NMR spectroscopic analyses.

## Conclusion

In total, 248 optimization experiments were conducted to optimize two distinct reactions: 128 prior to the use of UPBO and 120 as part of it (60 for N1/N2, respectively). These experiments were selected out of a search space of 8 million possible combinations of solvent with the other variable; 90 individual values for these variables (including 44 solvents) were evaluated and optimized to arrive at highly selective conditions without the use of high-performance computing resources. Remarkably, UPBO overcame an inherent bias toward acid-catalyzed condensation, based on Knorr precedent, to develop basic conditions for selective N1 and N2 pyrazole condensations. Under basic conditions at low temperatures, N1 and N2 hemiaminal intermediates were formed selectively and readily dehydrated to the pyrazole product upon workup. Importantly, the separation of the cyclization and dehydration events enables the selective formation of both N1 and N2 intermediates. The N2 intermediate was found by DFT calculations to be thermodynamically favored, even though the N2 pyrazole product is not the thermodynamic product. The N2 intermediate formation was found to be reversible and can be converted to N1 under suitable conditions. These findings demonstrate that Utopia Point Bayesian optimization can be a valuable tool to aid the synthetic chemist in the optimization of challenging reactions.

## Supporting Information Available

Experimental procedures and supporting data (PDF)
