# Supplementary material for: Utopia Point Bayesian Optimization Finds Condition-Dependent Selectivity for N-Methyl Pyrazole Condensation
Source: J Am Chem Soc. 2024 May 28;146(23):15779–86. doi: 10.1021/jacs.4c01616 (PMC11177315; doi:10.1021/jacs.4c01616)
Supplement: Supplementary file 1 — ja4c01616_si_001.pdf [file ja4c01616_si_001.pdf]

Utopia Point Bayesian Optimization Finds Condition Dependent Selectivity for *N*-Methyl Pyrazole Condensation

Derek M. Dalton,[a,‡,*] *Richard C. Walroth,*[a,‡] *Caroline Rouget-Virbel,*[b] *Kyle A. Mack,*[a] *F. Dean Toste*[b,*]

[a] *Department of Small Molecule Process Chemistry, Genentech, Inc., South San Francisco, California 94080, United States*

[b] *Department of Chemistry, University of California, Berkeley, California 94720, United States*

Table of Contents

Materials and Methods **3**

General Procedure for Pyrazole Cyclizations **4**

Bayesian Optimization Training Dataset **7**

Procedure for Bayesian Optimization **10**

Bayesian Optimization Results **12**

UP-BO Benchmarks **17**

Feature Importance **20**

Pyrazole Cyclization Reaction Profiles **23**

Pyrazole Cyclization Control Reactions **25**

NMR and MS Reaction Analysis of N2-selective conditions with Vinylogous Amide 1a. **29**

MS Reaction Analysis of N1-selective conditions with Vinylogous Amide 1a. **33**

DFT Calculations of 3a and 4a **35**

HPLC Determination of Product ratio **40**

Single Crystal X-ray Analysis of 3a......46

Materials and Methods

All solvents and reagents, including vinylogous amides, hydrazines, disulfonamide catalyst and phase transfer catalysts were obtained from commercial sources and used without further purifications. Powdered 4 angstrom molecular sieves were activated by evacuation in a round bottom flask on a Schlenk line with heat. Yields and product ratios are reported with respect to an internal biphenyl standard and determined by HPLC with an absorption correction factor applied. TLC: Macherey-Nagel, TLC plates Alugram®Sil G/UV254. Detection under UV light at 254 nm. For HRMS data, samples were introduced into the mass spectrometer (Thermo Scientific Orbitrap Lumos, San Jose, CA, USA) via direct infusion (via syringe) with an electrospray ionization (ESI) source with the capabilities to perform atmospheric pressure photoionization (APPI) to aid in the ionization, as described previously. Analytical High Performance Liquid Chromatography (HPLC) analyses were performed with an Agilent 1200 Series or an Agilent 1260 Infinity Series HPLC instrument with Xbridge C18 (3 x 100mm, 2.5 um) as column (flow rate = 0.7mL/min; ACN/H2O = 80:20; UV- detection at 254 and 225 nm). Flash column chromatography was performed with prepackaged RediSep silica gel columns on a CombiFlash ISCO system using *i*-PrOAc in heptanes as eluent. Specific gradients are listed under experiment procedures. NMR spectra were recorded at 500, 400 for ^{1}H-NMR; 200, 125, 101 for ^{13}C-NMR and 471, 376, 282 MHz for ^{19}F-NMR, respectively, on Bruker 400 MHz, 500 MHz; and AMX 300 instruments. Single Crystal X-ray crystallography data was collected with a Rigaku Synergy Custom SC-XRD with HyPix 6000 Hybrid Pixel Detector. If not otherwise specified, chemical shifts (δ) are given in ppm. The structure of the major constitutional isomer is shown.

General Procedure for Pyrazole Cyclizations

The following reaction screens were conducted in which each vial equipped with a magnetic stirbar was added in the amount specified in the materials section as specified in the table below.

Procedure of addition to the vial: (2E)-3-(dimethylamino)-1-(4-fluorophenyl)prop-2-en-1-one (0.010 g, 0.050 mmol, 97 mass%), As indicated the following compounds were added (note: not all compounds may be specified for addition, refer to table below for guidance): (11bS)-2,6-Bis[3,5-bis(trifluoromethyl)phenyl]dinaphtho[2,1-d:1',2'-f][1,3,2]dithiazepine 3,3,5,5-tetraoxide (0.1 equiv., 0.0050 mmol, 95 mass%), cetylpyridinium bromide (0.2 equiv., 0.010 mmol, 98 mass%) catalyst (s) as, 10 mg 4Å powdered molecular sieves (activated with high heat under vacuum, 1:1 g/g ratio of starting material to molecular sieves). Solvent was added via graduated syringe and the vial was placed in an aluminum block at the specified temperature. After 1-2 min, liquid reagents were added directly to the reaction mixture solution (not to the side of the vial wall – this is an issue with aqueous 50% NaOH due to high viscosity) via calibrated syringe in the following order, AcOH (glacial), NaOH (aq. 50%) and ultimately, methylhydrazine (98%).

Samples were analyzed for conversion and assay yield by reverse phase HPLC (6 uL reaction sample in 500 uL diluent: 8:2 ACN/Water). Assay yield was calculated with reference to biphenyl internal standard (g/g) add as a solution in DMSO with an absorbance correction factor applied when required.

Workup:

Aqueous workup for N1 reaction: For a 100 mg scale (starting material) reaction with 3 mL MTBE, 0.2 ml of Ac_2O is added to the reaction mixture while cooled and stirred for 30 min. 0.5 mL of sat. $NaHCO_3$ is added to the reaction mixture and 2mL of iPrOAc is added. Mixture is shaken and the aqueous layer is separated. 0.2 ml of water is added to aqueous solution and back extracted with iPrOAc (2-3x). Organic layers are combined, dried over $MgSO_4$, filtered and concentrated by rotary evaporation.

For N2 reaction conditions, 0.5 ml Ac_2O or 0.5 ml aqueous 1M TFA or 1M HCl solution is added to the reaction mixture after complete consumption of starting material as monitored by HPLC and stirred for 30 min. Residue can be concentrated directly by rotary evaporation in the case of acetic anhydride or the above aqueous reaction workup described above can be used.

Chromatography:

Residue is dissolved in a minimal amount of DCM and directly loaded onto a prepacked silica column. Purification is completed by flash chromatography (Combiflash) with gradient elution from 0 to 20% iPrOAc/heptanes over 30 min (30% iPrOAc is needed for pyridyl substrates).

HPLC Analysis Method: Column: XBridge BEH C18, 3x100mm, 2.5 um. Rate: 0.7mL/min, 30 ^{o}C, 220 nm UV detection. Mobile Phase A: 0.05% TFA in Water, Mobile Phase B: 0.05% TFA in ACN. Elution: 0-0.5' 2%B; 4' 35%B; 7-10' 90%B, 10.1-12' 2% B.

Representative Sample HPLC Spectrum showing starting material, intermediates, by product and desired isomeric products:

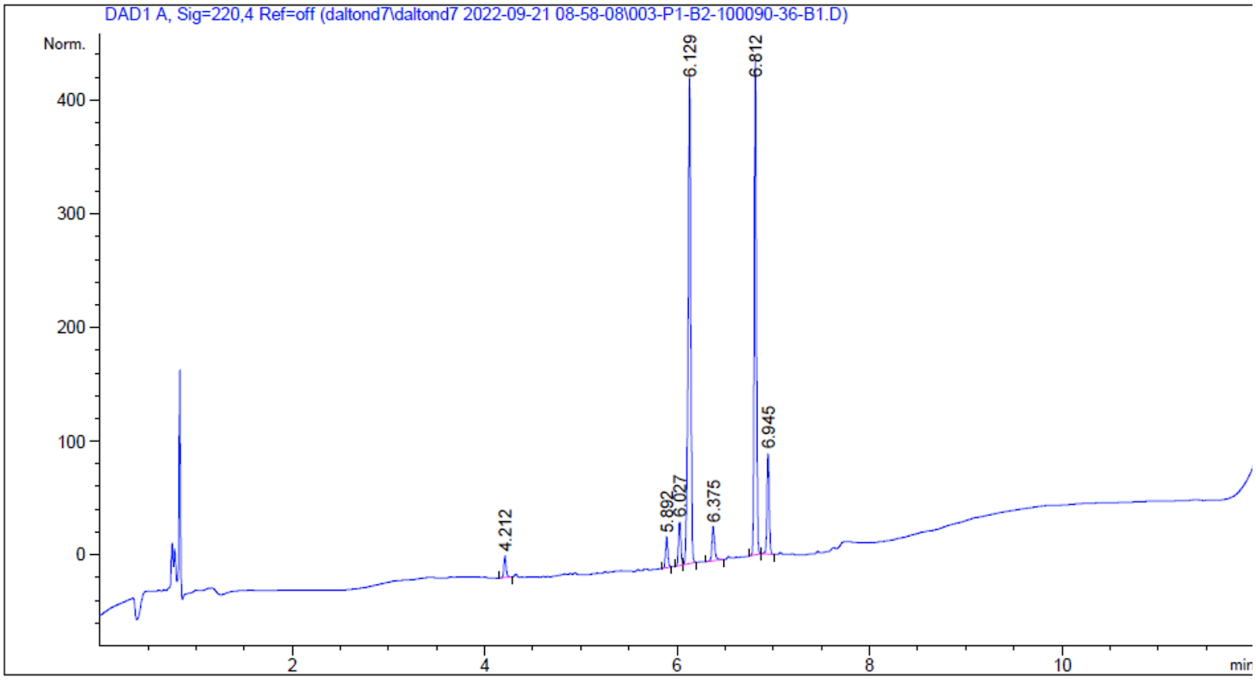

Figure SI-1. HPLC spectrum of crude reaction mixture containing vinylogous amide **1a**, pyrazole **2a**, N1 isomer **3a** and N2 isomer **4a**.

- N1 isomer **3a** observed at 6.812 min
- N2 Isomer **4a** observed at 6.945 min.
- Starting material **1a** observed at 6.129 min.
- NH-pyrazole **2a** 6.375 min.

Bayesian Optimization Training Dataset

Table SI-1. Initial BO training Dataset with substrate **1a**. Initial data set is highlighted in blue and expanded training data set is shown in red to correspond with Figure 2 in the text.

Rxn #	AcOH (equiv)	NaOH (equiv)	MeN2H3 (equiv)	DSI2 Catalyzed (1 - yes, 0 - no)	PTC Catalyzed (1/0)	4 Ang MS (1/0)	Temp °C	Solvent (V)	Solvent Name	SM Area*	N2 Area*	N1 Area*	Conv. to Product	N2'-N1'	N2'	N1'
1	0	0	1	1	0	0	5	60	DCM	76.0	19.0	4.0	23	65	83	17
2	0.2	0	1	1	0	0	5	60	DCM	67.0	18.0	10.0	28	29	64	36
3	0.8	0	1	1	0	0	5	60	DCM	40.0	32.0	22.0	54	19	59	41
4	1	0	1	1	0	0	5	60	DCM	43.0	32.0	22.0	54	19	59	41
5	1.5	0	1	1	0	0	5	60	DCM	33.0	38.0	26.0	64	19	59	41
6	2	0	1	1	0	0	5	60	DCM	43.0	32.0	21.0	53	21	60	40
7	0	0	3	1	0	0	5	60	DCM	72.0	21.0	6.0	27	56	78	22
8	0.2	0	3	1	0	0	5	60	DCM	70.0	16.0	11.0	27	19	59	41
9	0.8	0	3	1	0	0	5	60	DCM	28.0	29.0	27.0	56	4	52	48
10	1	0	3	1	0	0	5	60	DCM	18.0	39.0	38.0	77	1	51	49
11	1.5	0	3	1	0	0	5	60	DCM	6.0	47.0	42.0	89	6	53	47
12	2	0	3	1	0	0	5	60	DCM	8.0	43.0	42.0	85	1	51	49
13	0	0	5	1	0	0	5	60	DCM	38.0	48.0	10.0	58	66	83	17
14	0.2	0	5	1	0	0	5	60	DCM	20.0	60.0	16.0	76	58	79	21
15	0.8	0	5	1	0	0	5	60	DCM	32.0	37.0	26.0	63	17	59	41
16	1	0	5	1	0	0	5	60	DCM	27.0	34.0	29.0	63	8	54	46
17	1.5	0	5	1	0	0	5	60	DCM	6.0	49.0	40.0	89	10	55	45
18	2	0	5	1	0	0	5	60	DCM	1.0	54.0	44.0	98	10	55	45
19	0	0	1	0	0	0	5	60	DCM	92.0	0.0	1.0	1	-100	0	100
20	0.2	0	1	0	0	0	5	60	DCM	90.0	1.0	2.0	3	-33	33	67
21	0.8	0	1	0	0	0	5	60	DCM	62.0	10.0	20.0	30	-33	33	67
22	1	0	1	0	0	0	5	60	DCM	60.0	11.0	19.0	30	-27	37	63
23	1.5	0	1	0	0	0	5	60	DCM	58.0	16.0	23.0	39	-18	41	59
24	2	0	1	0	0	0	5	60	DCM	49.0	21.0	25.0	46	-9	46	54
25	0	0	3	0	0	0	5	60	DCM	96.0	0.0	1.0	1	-100	0	100
26	0.2	0	3	0	0	0	5	60	DCM	92.0	0.3	1.5	1.8	-67	17	83
27	0.8	0	3	0	0	0	5	60	DCM	67.0	5.0	11.0	16	-38	31	69
28	1	0	3	0	0	0	5	60	DCM	52.0	11.0	22.0	33	-33	33	67
29	1.5	0	3	0	0	0	5	60	DCM	42.0	15.0	26.0	41	-27	37	63
30	2	0	3	0	0	0	5	60	DCM	23.0	20.0	35.0	55	-27	36	64
31	0	0	5	0	0	0	5	60	DCM	84.0	9.0	7.0	16	13	56	44
32	0.2	0	5	0	0	0	5	60	DCM	72.0	13.0	14.0	27	-4	48	52
33	0.8	0	5	0	0	0	5	60	DCM	69.0	9.0	20.0	29	-38	31	69
34	1	0	5	0	0	0	5	60	DCM	76.0	7.0	16.0	23	-39	30	70
35	1.5	0	5	0	0	0	5	60	DCM	49.0	15.0	31.0	46	-35	33	67
36	2	0	5	0	0	0	5	60	DCM	40.0	20.0	39.0	59	-32	34	66
37	0	0	1	1	0	0	25	60	DCM	80.0	10.0	3.0	13	54	77	23
38	0.2	0	1	1	0	0	25	60	DCM	73.0	16.0	11.0	27	19	59	41
39	0.8	0	1	1	0	0	25	60	DCM	38.0	32.0	30.0	62	3	52	48
40	1	0	1	1	0	0	25	60	DCM	40.0	30.0	30.0	60	0	50	50
41	1.5	0	1	1	0	0	25	60	DCM	34.0	35.0	30.0	65	8	54	46
42	2	0	1	1	0	0	25	60	DCM	30.0	38.0	31.0	69	10	55	45
43	0	0	3	1	0	0	25	60	DCM	73.0	11.0	5.0	16	38	69	31
44	0.2	0	3	1	0	0	25	60	DCM	58.0	14.0	7.0	21	33	67	33
45	0.8	0	3	1	0	0	25	60	DCM	24.0	38.0	37.0	75	1	51	49
46	1	0	3	1	0	0	25	60	DCM	24.0	35.0	36.0	71	-1	49	51
47	1.5	0	3	1	0	0	25	60	DCM	11.0	39.0	45.0	84	-7	46	54

48	2	0	3	1	0	0	25	60	DCM	3.0	40.0	54.0	94	-15	43	57
49	0	0	5	1	0	0	25	60	DCM	73.0	17.0	6.0	23	48	74	26
50	0.2	0	5	1	0	0	25	60	DCM	67.0	20.0	7.0	27	48	74	26
51	0.8	0	5	1	0	0	25	60	DCM	28.0	28.0	28.0	56	0	50	50
52	1	0	5	1	0	0	25	60	DCM	16.0	35.0	46.0	81	-14	43	57
53	1.5	0	5	1	0	0	25	60	DCM	71.0	20.0	7.0	27	48	74	26
54	2	0	5	1	0	0	25	60	DCM	1.0	44.0	54.0	98	-10	45	55
55	0	0	1	0	0	0	25	60	DCM	99.0	0.0	0.0	0	0	0	0
56	0.2	0	1	0	0	0	25	60	DCM	94.0	1.0	3.0	4	-50	25	75
57	0.8	0	1	0	0	0	25	60	DCM	68.0	9.0	17.0	26	-31	35	65
58	1	0	1	0	0	0	25	60	DCM	69.0	10.0	17.0	27	-26	37	63
59	1.5	0	1	0	0	0	25	60	DCM	56.0	16.0	24.0	40	-20	40	60
60	2	0	1	0	0	0	25	60	DCM	47.0	23.0	30.0	53	-13	43	57
61	0	0	3	0	0	0	25	60	DCM	99.0	0.0	0.0	0	0	0	0
62	0.2	0	3	0	0	0	25	60	DCM	98.0	0.0	1.0	1	-100	0	100
63	0.8	0	3	0	0	0	25	60	DCM	71.0	8.0	18.0	26	-38	31	69
64	1	0	3	0	0	0	25	60	DCM	75.0	7.0	13.0	20	-30	35	65
65	1.5	0	3	0	0	0	25	60	DCM	44.0	18.0	33.0	51	-29	35	65
66	2	0	3	0	0	0	25	60	DCM	32.0	23.0	40.0	63	-27	37	63
67	0	0	5	0	0	0	25	60	DCM	100.0	0.0	0.0	0	0	0	0
68	0.2	0	5	0	0	0	25	60	DCM	97.0	0.6	1.8	2.4	-50	25	75
69	0.8	0	5	0	0	0	25	60	DCM	87.0	4.0	8.0	12	-33	33	67
70	1	0	5	0	0	0	25	60	DCM	86.0	4.0	9.0	13	-38	31	69
71	1.5	0	5	0	0	0	25	60	DCM	60.0	13.0	25.0	38	-32	34	66
72	2	0	5	0	0	0	25	60	DCM	45.0	19.0	31.0	50	-24	38	62
73	0	0	5	0	0	0	5	30	DCM	774.0	15.0	24.6	4.9	-24	38	62
74	0	0	5	0	0	1	5	30	DCM	1322.0	11.0	15.5	1.9	-17	42	58
75	0	0	5	1	0	1	5	30	DCM	781.0	611.0	407.5	56.1	20	60	40
76	0	0	5	1	0	0	5	60	DCM	991.2	675.3	400.6	51.5	26	63	37
77	0	0	5	1	0	0	5	120	DCM	974.1	533.0	273.9	45.3	32	66	34
78	0	3	5	0	0	0	5	30	DCM	65.9	18.5	7.9	0	40	70	30
79	0	3	5	0	0	0	5	30	DCM	806.0	5.0	115.0	12.9	-92	4	96
80	0	1.5	5	0	0	0	5	30	DCM	284.0	0.0	0.0	0	0	0	0
81	0	5	5	0	1	0	5	30	DCM	854.0	0.0	67.3	7.3	-100	0	100
82	0	5	5	0	1	0	5	20	DCM	1392.0	1.0	448.5	23.4	-100	0	100
83	0	5	5	0	1	0	5	10	DCM	1691.0	13.0	991.5	33.3	-97	1	99
84	0	0	5	0	1	0	5	10	DCM	3675.6	177.6	62.4	28	48	74	26
85	0	0	5	1	1	0	5	10	DCM	2094.3	38.6	174.8	6.1	-64	18	82
1	0	0	5	0	0	0	5	30	THF	249.0	463.0	176.5	69.7	45	72	28
2	0	0	5	0	0	0	5	30	CPME	491.0	595.0	152.4	58.3	59	80	20
3	0	0	5	0	0	1	5	30	EtOH	387.0	159.0	253.6	44.6	-23	39	61
4	0	0	5	0	0	1	5	30	iPrOH	196.0	603.0	149.6	77.4	60	80	20
5	0	0	5	0	0	1	5	30	TFE	876.0	414.0	1796.3	71.3	-63	19	81
6	0	0	5	0	0	0	5	30	PhMe	283.0	98.0	39.5	32.3	43	71	29
7	0	0	5	1	0	0	5	30	PhMe	157.0	169.0	81.8	59.9	35	67	33
8	0	0	5	0	0	0	5	30	MeCN	160.0	5.0	27.6	5.9	-69	15	85
9	0	0	5	1	0	0	5	30	MeCN	157.0	184.0	105.8	63.3	27	63	37
10	0	0	5	0	0	0	5	30	iPAc	391.0	32.0	17.3	11.2	30	65	35
11	0	0	5	1	0	0	5	30	iPAc	292.0	467.0	130.1	66	56	78	22
12	0	0	5	0	0	0	5	30	MeOH	203.0	119.0	168.1	55.6	-17	41	59
13	0	0	5	1	0	0	5	30	MeOH	166.0	235.0	141.1	67.9	25	62	38
14	0	0	5	0	0	0	5	30	MTBE	136.0	270.0	40.6	67.8	74	87	13
15	0	0	5	1	0	0	5	30	MTBE	32.0	428.0	217.5	92.1	33	66	34
16	0	0	5	1	0	1	5	30	PhMe	350.0	545.0	243.4	69.3	38	69	31
17	0	0	5	1	0	1	5	30	MeCN	542.0	241.0	176.1	42.1	16	58	42

18	0	0	5	1	0	1	5	30	iPAc	508.0	1015.0	546.0	68.5	30	65	35
19	0	0	5	1	0	1	5	30	MeOH	454.0	79.0	400.1	45.8	-67	16	84
20	0	0	5	1	0	1	5	30	MTBE	189.0	1061.0	302.4	86.7	56	78	22
21	0	0	1	0	0	1	5	30	MTBE	456.0	37.0	26.5	12	17	58	42
22	0	0	3	0	0	1	5	30	MTBE	419.0	189.0	72.5	34.4	45	72	28
23	0	0	5	0	0	1	5	30	MTBE	283.0	354.0	71.0	49.1	67	83	17
24	0	0	1	1	0	1	5	30	MTBE	470.0	244.0	37.3	36.8	73	87	13
25	0	0	3	1	0	1	5	30	MTBE	259.0	666.0	179.8	73.2	57	79	21
26	0	0	1	0	0	0	5	30	MTBE	1174.0	41.0	44.9	6.8	-5	48	52
27	0	0	3	0	0	0	5	30	MTBE	407.0	120.0	52.2	29.3	39	70	30
28	0	0	10	0	0	0	5	30	MTBE	12.0	1,420	72.3	98.1	90	95	5
29	0	0	20	0	0	0	5	30	MTBE	0.0	887	23.3	98.7	95	97	3
30	0	0	5	0	0	0	5	20	MTBE	397.0	409	60.4	52.6	74	87	13
31	0	0	5	0	0	0	5	10	MTBE	110.0	2,156	272.4	93.6	78	89	11
32	0	0	5	0	0	0	5	5	MTBE	520.0	1,739	195.5	76.6	80	90	10
33	0	0	5	0	0	1	5	30	MeOH	415.0	49.0	356.5	42.7	-76	12	88

Procedure for Bayesian Optimization

Bayesian optimization was carried out using previously published algorithms implemented in python using the Scikit-Learn library [Brochu 2010, Pedregosa et al, 2011, version 1.1.2]. Ensemble models such as random forest were modified to return a standard deviation and mean for the individual estimator predictions. For this work, each round consisted of ten to sixteen experiments conducted in parallel. These were split evenly between those targeting N1 selectivity and N2 selectivity. In order to recommend multiple sets of conditions, five models were trained: Gaussian process, random forest, extra random trees, gradient boost, and Bayesian neural network. Each one was used to predict both yield and selectivity initially, and then the inverse distance metric (vide infra). Their predictions and uncertainties were used in conjunction with two acquisition functions: expected improvement and upper confidence bound (see Brochu et al). Solvents were parameterized using the COSMO-RS parameters as reported by Moity et al [Moity 2012]. Use of molecular sieves or use of either catalyst were modeled as Boolean variables. Numeric variables such as temperature or solvent volume were scaled using a standard scaler prior to fitting. Equivalents of acetic acid and NaOH were combined into one numeric variable, with positive values equal to equivalents of acetic acid and negative values equal to the negative of NaOH equivalents. Boolean variables were modeled in the Gaussian process kernel using a transformed kernel as described by Garrido-Merchán et al [Garrido-Merchán 2020]. Data from all experiments were pooled for predicting subsequent rounds.

Initially, yield and selectivity were independently modeled and the acquisition function values were balanced by selecting from pareto optimal points. For Utopia Point Bayesian Optimization (UPBO), the objectives were scalarized by measuring their distance to a hypothetical optimum point:

$$\boldsymbol{d} = \sqrt{\boldsymbol{a}^2\left(\mathbf{1} - {}^{\boldsymbol{t}_1}\!/\!_{\boldsymbol{t}_{1u}}\right)^2 + \boldsymbol{b}^2\left(\mathbf{1} - {}^{\boldsymbol{t}_2}\!/\!_{\boldsymbol{t}_{2u}}\right)^2}$$

Here, a and b refer to the weights for each objective, t_1 and t_2 refer to the two objectives, and t_{1u} and t_{2u} refer to the coordinates of the utopia point. The models were trained on the inverse of the distance, d, and then treated as a single objective optimization problem. A yield of 110% and dr of 99.95:0.05 were used as coordinates of

the utopia point. Both weights were set to 1. In order to minimize distance, the models were trained on $1/d$ instead of directly modeling on d.

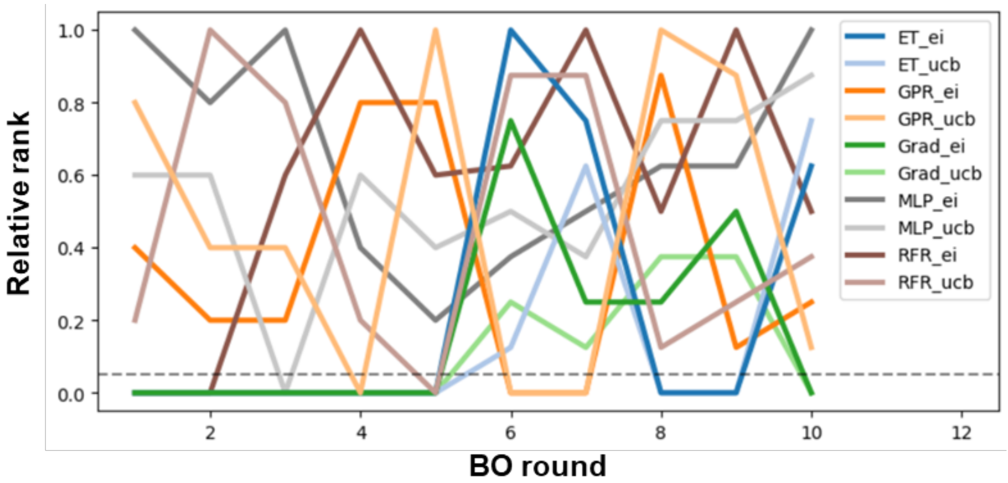

Figure SI-2: Relative performance of model/acquisition function combinations. GPR: Gaussian process; MLP: multi-layer perceptron; Grad: gradient boost; RFR: random forest; ET: extra random trees; ei: expected improvement; ucb: upper confidence bound.

Brochu, Eric, Vlad M. Cora, and Nando De Freitas. "A tutorial on Bayesian optimization of expensive cost functions, with application to active user modeling and hierarchical reinforcement learning." arXiv preprint arXiv:1012.2599 (2010).

Scikit-learn: Machine Learning in Python, Pedregosa et al., JMLR 12, pp. 2825-2830, 2011.

Moity, L., Durand, M., Benazzouz, A., Pierlot, C., Molinier, V., & Aubry, J. M. (2012). Panorama of sustainable solvents using the COSMO-RS approach. Green chemistry, 14(4), 1132-1145.

Garrido-Merchán, Eduardo C., and Daniel Hernández-Lobato. "Dealing with categorical and integer-valued variables in bayesian optimization with gaussian processes." Neurocomputing 380 (2020): 20-35.

Bayesian Optimization Results

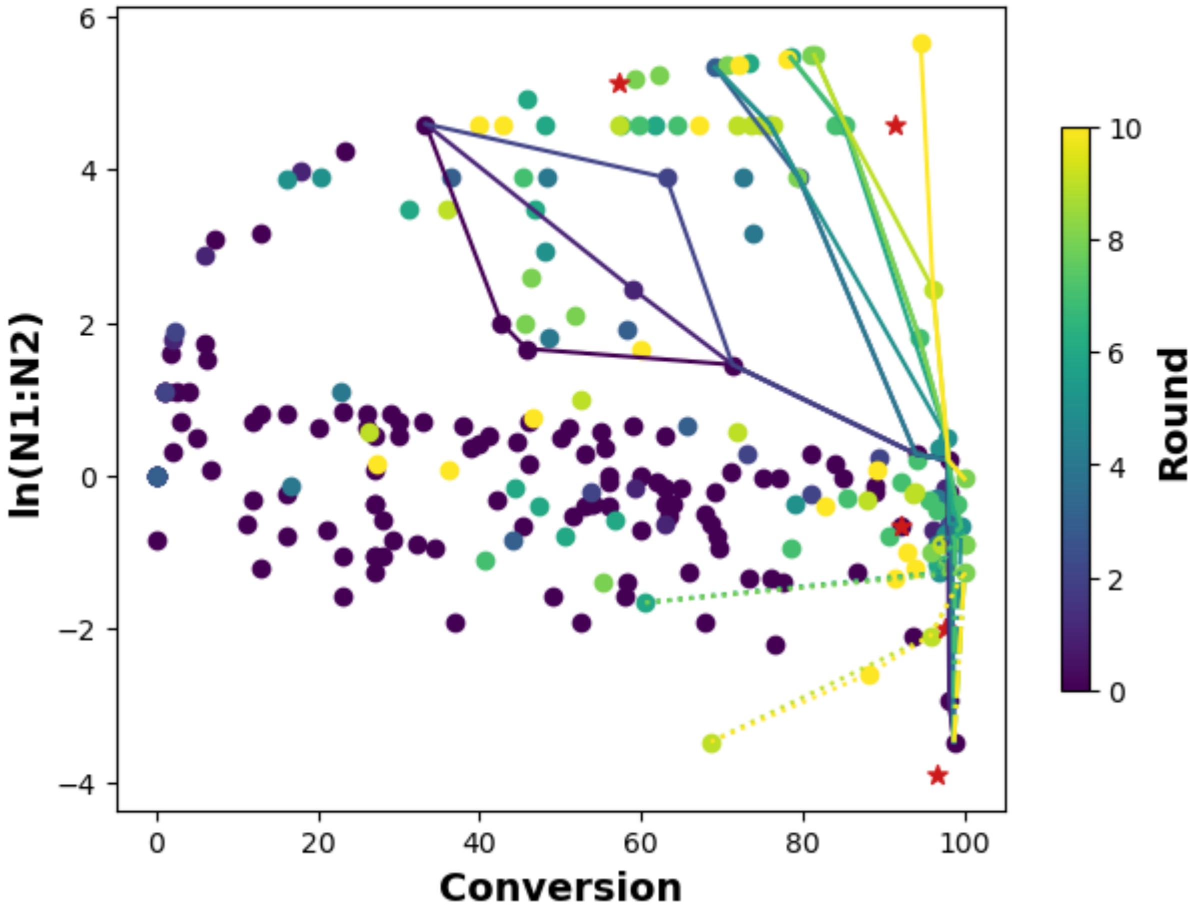

Figure SI-3: Plot of results for 10 rounds of Bayesian optimization. Points are color coded by round. Lines represent Pareto front for total dataset up to that round (solid for N1 selective, dashed for N2 selective not including preliminary data). Red stars represent results for predicted best conditions.

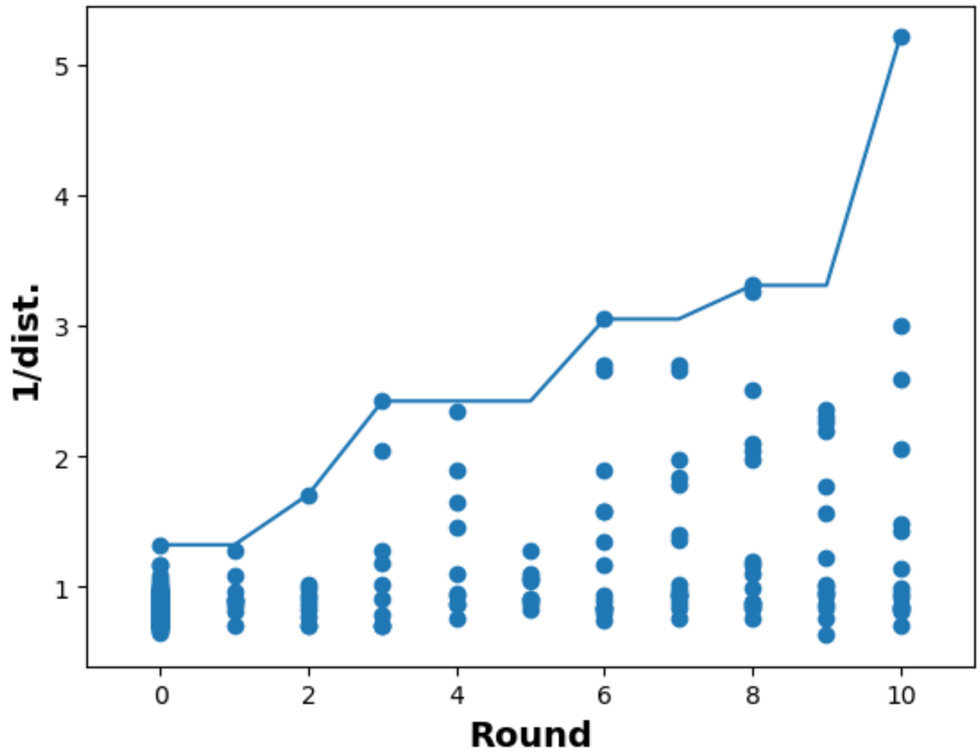

Figure SI-4: Plot of inverse distance to utopia point for N1 vs Bayesian optimization round. Lines represent running maxima at each round.

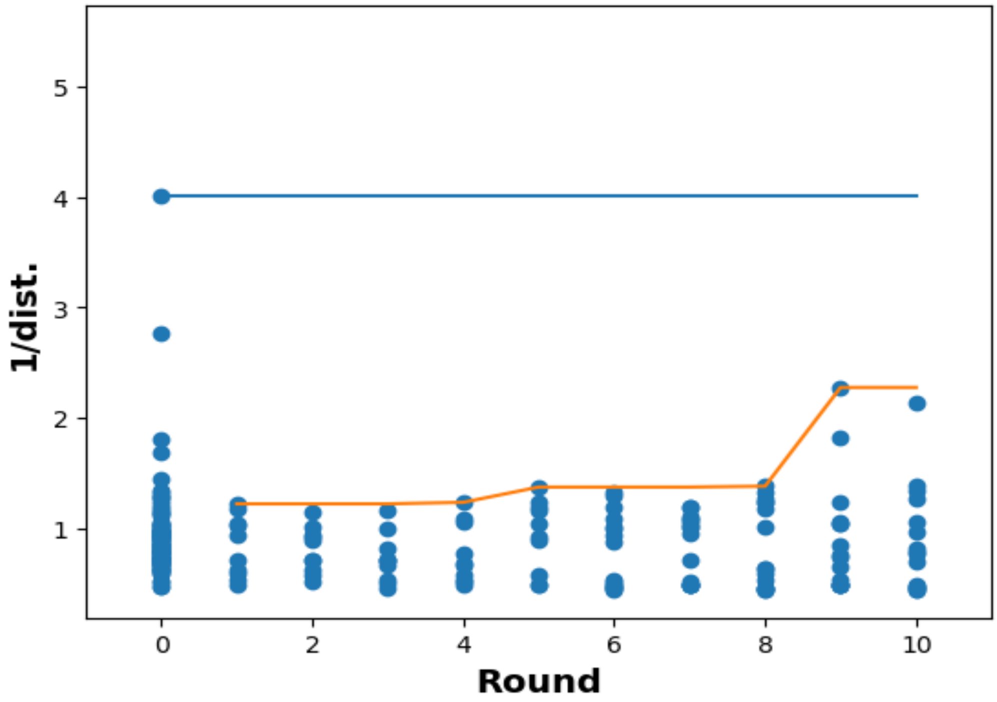

Figure SI-5: Plot of inverse distance to utopia point for N2 vs Bayesian optimization round. Lines represent running maxima at each round.

Table SI-2. Results of Bayesian Optimization with **1a**.

BO Round	BO Model	AcOH equiv	NaOH equiv	MeN_2H_3 equiv	DSI2 (1/0)	PTC (1/0)	4 Ang MS (1/0)	Temp ºC	Sol. V (mL/g)	Solvent Name	SM (area count)	N2 (area count)	N1 (area count)	Assay Yield	N1'-N2'	N2'	N1'
**BO1**	GPR_ei_01_n1	0	1	1	1	1	1	0	10	MTBE	841.4	0.0	0.0	0	0	0	0
	GPR_ucb_2_n1	1	0	1	1	1	1	0	10	MTBE	680.7	756.2	402.7	63	31	65	35
	RFR_ei_01_n1	0	5	2	0	1	0	50	10	ethyl acetate	753.3	838.2	701.8	59.2	9	54	46
	RFR_ucb_2_n1	0	5	1	0	1	0	25	10	N,N-DMF	767.8	0.0	36.9	2	-100	0	100
	MLP_ei_01_n1	5	0	5	0	1	0	50	10	tributylamine	23.8	6065.9	2963.0	96.2	34	67	33
	GPR_ei_01_n2	3	0	3	1	1	1	25	10	DPMU	26.7	1899.4	1637.0	97.7	7	54	46
	GPR_ucb_2_n2	5	0	5	1	1	1	50	30	p-Cymene	0.0	1217.3	530.3	**97.7**	**39**	**70**	**30**
	RFR_ucb_2_n2	0	5	2	1	0	1	50	30	2-MeTHF	247.0	50.4	606.4	**58.9**	**-85**	**8**	**92**
	MLP_ei_01_n2	0	5	1	0	0	1	50	30	tributylamine	540.5	0.0	57.6	6	-100	0	100
	MLP_ucb_2_n2	0	5	1	0	0	1	25	10	tributylamine	1035.6	0.0	276.2	17.8	-100	0	100
**BO2**	GPR_ei_05_n1	2	0	5	1	0	0	0	10	triethylamine	1043.8	681.2	563.3	53.7	9	55	45
	RFR_ei_05_n1	2	0	5	1	0	0	25	20	1,1,2,2-tetrachloroethane	23.2	2924.2	3806.6	73	-13	43	57
	RFR_ucb_2_n1	5	0	5	1	0	0	50	30	1,1,2,2-tetrachloroethane	39.2	3409.0	2705.9	81	11	56	44
	MLP_ei_05_n1	3	0	5	1	1	1	50	10	MeCN	0.0	3767.3	2041.9	**98.1**	**30**	**65**	**35**
	MLP_ucb_2_n1	5	0	1	1	0	0	50	30	formamide	222.8	1186.0	1533.8	89.3	-13	44	56
	GPR_ei_05_n2	0	4	1	0	0	1	0	10	triethylamine	1445.2	0.0	0.0	0	0	0	0
	GPR_ucb_2_n2	0	4	1	0	1	1	0	10	triethylamine	2161.7	0.0	0.0	0	0	0	0
	RFR_ucb_2_n2	0	5	5	0	0	1	25	10	diethylamine	1497.1	77.6	4760.1	**63.1**	**-97**	**2**	**98**
	MLP_ei_05_n2	0	5	5	0	1	1	0	10	formamide	2337.7	0.0	65.0	2.2	-100	0	100
	MLP_ucb_2_n2	0	5	1	0	0	0	0	10	carbon tetrachloride	3125.1	0.0	33.5	1	-100	0	100
**BO3**	GPR_ei_05_n1	0	0.56	5	1	1	1	0	10	alpha-Terpineol	1077.3	692.1	301.5	44.1	39	70	30
	GPR_ucb_2_n1	0	0.56	5	1	1	1	50	10	1-decanol	897.8	238.6	1668.1	58.2	-75	13	87
	RFR_ei_05_n1	0	2.78	5	0	1	0	25	10	MTBE	610.6	47.2	2429.5	**79.5**	**-96**	**2**	**98**
	MLP_ei_05_n1	5	0.00	1	1	0	1	50	30	Dimethyl sulfoxide	2015.6	1342.5	2630.8	65.6	-32	34	66
	MLP_ucb_2_n1	5	0.00	5	0	0	0	0	10	Isoamyl alcohol	0.0	2726.0	1407.1	**98.4**	**32**	**66**	**34**
	GPR_ei_05_n2	0	3.89	5	1	1	0	50	10	tetrachloroethylene	1850.6	0.0	0.0	0	0	0	0
	GPR_ucb_2_n2	0	3.89	5	1	0	0	50	10	tetrachloroethylene	2789.7	0.0	0.0	0	0	0	0
	RFR_ei_05_n2	0	2.78	5	0	0	0	37.5	10	Perfluorooctane	512.6	0.0	0.0	0	0	0	0
	RFR_ucb_2_n2	0	5	5	0	0	1	37.5	10	di-n-butyl ether	1304.0	74.3	3519.3	36.4	-96	2	98
	MLP_ei_05_n2	0	5	5	1	1	1	12.5	23.3	1-decanol	307.4	0.0	1339.8	69.1	-100	0	100
**BO4**	GPR_ei_05_n1	5	0.00	3	0	1	0	25	10	cyclohexane	10.9	4068.3	1692.9	**96.6**	**41**	**71**	**29**
	GPR_ucb_2_n1	5	0.00	1	0	1	1	25	10	cyclopentane	84.3	2681.3	2010.3	96.7	14	57	43
	RFR_ucb_2_n1	0	1.67	5	0	0	0	12.5	10	MTBE	673.6	83.2	2293.5	73.8	-93	4	96
	MLP_ei_05_n1	5	0.00	5	1	1	1	0	30	tributylamine	487.7	51.9	46.0	16.7	6	53	47
	MLP_ucb_2_n1	5	0.00	5	0	1	0	50	10	nitroethane	0.0	2723.5	1779.4	97.8	21	60	40
	GPR_ei_05_n2	0	2.78	5	1	1	0	37.5	10	triethylamine	790.8	22.8	1271.2	48.3	-96	2	98
	RFR_ei_05_n2	0	3.89	5	1	0	1	12.5	10	tributylamine	217.9	19.4	1007.4	72.5	-96	2	98
	RFR_ucb_2_n2	0	2.78	5	0	1	0	12.5	10	tributylamine	495.6	10.1	1986.4	**75.9**	**-99**	**1**	**99**
	MLP_ei_05_n2	0	3.89	1	1	1	0	37.5	30	m-cresol	10681.8	803.7	2412.0	22.8	-50	25	75
	MLP_ucb_2_n2	0	5	1	0	0	0	50	10.0	Glycerol	243.8	127.6	777.2	48.5	-72	14	86
**BO5**	GPR_ei_05_n1	3.89	0	5	0	1	1	50	10	1,8-Cineol	0	1353.4	559.2	98.4	42	71	29
	GPR_ucb_2_n1	3.89	0	5	0	0	1	50	10	1,8-Cineol	0	2006.9	956.3	97.6	35	68	32
	RFR_ei_05_n1	0.56	0	5	0	0	1	50	10	THF	5.3	3762.6	6114.5	97.8	-24	38	62
	MLP_ei_05_n1	5	0	5	0	0	0	0	23	Perfluorooctane	265.8	634.3	436	79.1	19	59	41
	MLP_ucb_2_n1	5	0	5	1	1	0	12.5	10	m-cresol	0	3556.8	977.2	**96.8**	**57**	**78**	**22**
	GPR_ei_05_n2	0	1.7	5	0	0	1	0	30	tributylamine	1564.8	13.9	556.2	20.3	-95	2	98
	GPR_ucb_2_n2	0	1.7	5	0	0	0	25	10	triethylamine	894.8	52.1	932.5	**48.1**	**-89**	**5**	**95**
	RFR_ei_05_n2	0	1.7	5	0	1	0	0	17	tributylamine	402	0	108.6	16	-100	0	100

	MLP_ei_05_n2	3.89	0	3	0	0	0	50	10	sulfolane	6.7	4420.2	2238.5	99.6	33	66	34
	MLP_ucb_2_n2	5	0	1	0	1	1	37.5	30	benzonitrile	37.4	573.9	833.3	96.9	-18	41	59
**BO6**	*RFR_ei_05_n1*	*0.0*	*0.6*	*5*	*0*	*1*	*0*	*12.5*	*10*	*MTBE*	*609.6*	*1033.9*	*196.2*	***60.5***	***68***	***84***	***16***
	RFR_ucb_2_n1	0.0	0.6	4	0	0	0	12.5	10	MTBE	750.5	604.1	276.7	50.5	37	69	31
	Grad_ei_05_n1	5.0	0.0	3	0	0	1	50.0	10	2-butanol	0	2235.5	1070.7	98.8	35	68	32
	Grad_ucb_2_n1	5.0	0.0	4	0	0	1	50.0	10	CPME	0	2754.3	1866.2	98.5	19	60	40
	ET_ei_05_n1	0.0	0.6	5	0	0	0	0.0	10	MTBE	550.8	300.9	255.1	44.3	8	54	46
	ET_ucb_2_n1	0.0	0.6	5	0	0	0	12.5	10	MTBE	768.5	479.4	320.3	47.3	20	60	40
	MLP_ei_05_n1	5.0	0.0	5	1	0	1	0.0	10	di-n-butyl ether	431.3	359.9	206.3	56.8	27	64	36
	MLP_ucb_2_n1	5.0	0.0	5	0	0	1	0.0	10	HMPTA	23.6	1739	550.6	96.7	52	76	24
	RFR_ei_05_n2	0.0	5.0	4	1	1	1	12.5	23	1-Octanol	112.7	0	1018.9	73.2	-100	0	100
	RFR_ucb_2_n2	0.0	5.0	1	1	1	1	12.5	23	1-decanol	244.2	0	301.9	45.9	-100	0	100
	Grad_ei_05_n2	*0.0*	*5.0*	*5*	*1*	*1*	*1*	*12.5*	*23*	*Oleyl alcohol*	*89.9*	*11.7*	*1669.6*	***85***	***-99***	***1***	***99***
	Grad_ucb_2_n2	0.0	5.0	4	1	1	1	12.5	30	Oleyl alcohol	48.7	0	836.3	78.5	-100	0	100
	ET_ei_05_n2	0.0	5.0	5	1	1	1	0.0	23	1-decanol	415.8	7.8	297	31.3	-95	3	97
	ET_ucb_2_n2	0.0	5.0	5	1	1	1	0.0	30	1-decanol	344.5	5.6	497.7	48	-98	1	99
	MLP_ei_05_n2	0.0	5.0	5	1	1	0	0.0	10	1-decanol	2347.2	78.3	2831.3	46.7	-95	3	97
	MLP_ucb_2_n2	0.0	5.0	5	1	1	0	12.5	10	1-decanol	1242.6	25.3	3695.9	61.7	-99	1	99
**BO7**	*RFR_ei_05_n1*	*0.556*	*0*	*5*	*0*	*0*	*0*	*0*	*10*	*MTBE*	*253*	*734*	*279.3*	***78.4***	***45***	***72***	***28***
	RFR_ucb_2_n1	0.556	0	5	0	0	0	12.5	10	MTBE	180.3	1697.4	752.6	90.7	39	69	31
	Grad_ei_05_n1	5	0	5	0	1	0	50	10	Dimethyl adipate	0	2609	1974.3	85.4	14	57	43
	Grad_ucb_2_n1	3.889	0	5	0	1	1	50	10	HMPTA	0	1906.8	1406.3	95.7	15	58	42
	ET_ei_05_n1	0.556	0	5	0	0	0	25	10	MTBE	0	2950.8	1847.6	96.6	23	61	39
	ET_ucb_2_n1	0.556	0	5	0	0	0	0	10	1,8-Cineol	809	428.2	145.5	40.6	49	75	25
	MLP_ei_05_n1	5	0	5	0	0	0	12.5	10	HMPTA	119.5	1793.6	1649.7	92.2	4	52	48
	MLP_ucb_2_n1	5	0	1	0	1	0	50	10	m-cresol	0	2293.7	1596.6	99.1	18	59	41
	RFR_ei_05_n2	0	5	3	1	1	1	12.5	23.333	1-decanol	854.8	16.3	1730.9	57.5	-98	1	99
	RFR_ucb_2_n2	0	5	1	1	1	1	12.5	23.333	1-decanol	1043.3	19	1106.9	45.4	-97	2	98
	Grad_ei_05_n2	*0*	*5*	*5*	*1*	*0*	*1*	*12.5*	*30*	*tributylamine*	*84.6*	*3.8*	*609.5*	***85.2***	***-99***	***1***	***99***
	Grad_ucb_2_n2	0	5	5	1	1	1	12.5	30	tributylamine	125.9	4.2	814.3	84	-99	1	99
	ET_ei_05_n2	0	5	4	1	1	1	12.5	23.333	1-decanol	395.8	12.1	1375.6	64.4	-98	1	99
	ET_ucb_2_n2	0	3.889	5	1	1	1	12.5	23.333	1-decanol	779.6	10.6	1926.2	59.8	-99	1	99
	MLP_ei_05_n2	0	5	5	0	1	1	50	10	TFE	454.4	1365.3	8461.6	94.4	-72	14	86
	MLP_ucb_2_n2	0	5	5	0	1	0	37.5	10	m-cresol	0	3573.5	4310.7	94.0	-9	45	55
**BO8**	GPR_ei_05_n1	3.889	0	5	0	1	1	0	8	m-cresol	0.0	550.3	224.2	100.0	42	71	29
	GPR_ucb_2_n1	3.889	0	5	0	0	1	0	8	m-cresol	0.0	531.6	212.1	100.0	43	71	29
	RFR_ei_05_n1	0.556	0	5	1	1	0	12.5	8	m-cresol	0.0	702.1	254.0	95.9	47	73	27
	RFR_ucb_2_n1	*5*	*0*	*5*	*1*	*1*	*0*	*12.5*	*8*	*m-cresol*	*0.0*	*414.3*	*114.2*	***100.0***	***57***	***78***	***22***
	Grad_ei_05_n1	0.556	0	5	1	0	1	0	8	HMPTA	117.9	547.8	133.6	55.3	61	80	20
	Grad_ucb_2_n1	0	5	5	1	0	0	50	16	m-cresol	0.0	175.6	170.7	100.0	1	51	49
	MLP_ei_05_n1	0	3.889	5	1	0	0	50	8	DMSO	256.8	32.7	457.3	46.2	-87	7	93
	MLP_ucb_2_n1	1.667	0	1	0	1	0	0	8	TFE	0.0	1442.2	451.1	97.9	52	76	24
	GPR_ei_05_n2	0	5	5	1	0	1	25	30	Butyl stearate	212.9	26.9	1549.4	79.2	-97	2	98
	GPR_ucb_2_n2	0	5	5	1	1	1	12.5	30	1,8-Cineol	67.5	0.0	202.0	62.1	-100	0	100
	RFR_ei_05_n2	0	5	4	1	1	0	25	30	Oleyl alcohol	41.8	0.0	267.2	70.6	-100	0	100
	RFR_ucb_2_n2	0	5	4	1	0	1	50	30	Oleyl alcohol	49.5	0.0	206.8	59.2	-100	0	100
	Grad_ei_05_n2	0	5	4	1	1	1	12.5	30	Oleyl alcohol	18.5	0.0	225.1	80.9	-100	0	100
	Grad_ucb_2_n2	*0*	*5*	*4*	*1*	*0*	*1*	*12.5*	*30*	*Oleyl alcohol*	*28.5*	*0.0*	*364.9*	***81.4***	***-100***	***0***	***100***
	MLP_ei_05_n2	0	5	5	1	0	1	50	30	formamide	64.7	51.8	391.7	45.5	-77	12	88
	MLP_ucb_2_n2	0	5	5	0	1	0	50	30	formamide	63.7	73.3	606.4	51.8	-78	11	89
**BO9**	*GPR_ei_05_n1*	*0.556*	*0*	*16*	*0*	*0*	*0*	*0*	*23.333*	*n-propylamine*	*0.0*	*1035.3*	*35.0*	***68.6***	***93***	***97***	***3***
	GPR_ucb_2_n1	0	1.667	20	1	0	0	0	30	NMP	711.3	167.1	295.7	26.3	-28	36	64
	RFR_ei_05_n1	0	0.556	17	1	1	1	37.5	30	tributylamine	153.7	187.8	330.2	71.9	-27	36	64
	RFR_ucb_2_n1	0	0.556	18	1	1	1	50	16.667	1-Octanol	145.0	96.8	267.8	52.5	-47	27	73
	Grad_ei_05_n1	0	5	17	1	0	0	50	16.667	m-cresol	0.0	939.7	761.4	93.9	10	55	45

	Grad_ucb_2_n1	0	5	18	1	0	0	50	16.667	m-cresol	0.0	1060.4	824.6	93.7	13	56	44
	MLP_ei_05_n1	5	0	20	1	0	0	0	30	HMPTA	13.5	1806.8	214.7	95.8	79	89	11
	MLP_ucb_2_n1	5	0	20	1	0	1	0	30	triethylamine	13.0	1533.2	612.7	97.1	43	71	29
	GPR_ei_05_n2	0	5	5	1	0	1	12.5	30	m-cresol	0.0	962.0	696.9	87.8	16	58	42
	GPR_ucb_2_n2	0	2.778	8	0	0	0	12.5	10	MTBE	1286.1	17.3	3197.1	57.1	-99	1	99
	RFR_ei_05_n2	0	5	4	0	0	1	25	30	Oleyl alcohol	62.6	5.4	566.7	73.6	-98	1	99
	RFR_ucb_2_n2	0	5	3	0	0	1	25	30	Oleyl alcohol	105.2	7.9	990.4	71.8	-98	1	99
	Grad_ei_05_n2	0	5	4	1	1	0	25	30	Oleyl alcohol	158.0	14.1	1170.6	74.9	-98	1	99
	Grad_ucb_2_n2	0	5	4	1	0	1	25	30	Oleyl alcohol	108.9	11.2	1033.3	76.2	-98	1	99
	MLP_ei_05_n2	0	5	20	1	0	0	50	30	triethylamine	1055.1	42.2	1330.5	35.8	-94	3	97
	MLP_ucb_2_n2	*0*	*5*	*20*	*1*	*0*	*0*	*50*	*30*	*tributylamine*	*32.4*	*64.4*	*739.8*	***96.1***	***-84***	***8***	***92***
**BO10**	*GPR_ei_05_n1*	*0.556*	*0*	*20*	*0*	*1*	*0*	*0*	*10*	*pyrrolidine*	*0*	*1579.1*	*123.0*	***88.2***	***86***	***93***	***7***
	GPR_ucb_2_n1	2.778	0	20	0	1	0	50	10	formamide	123	3905	4292.6	89.1	-5	48	52
	RFR_ei_05_n1	0	0.556	20	0	0	0	50	10	MeTHF	1183.2	396.2		27.2	-9	46	54
	RFR_ucb_2_n1	0.556	0	19	0	0	0	25	30	Tetrahydrofurfurylic alcohol	23.6	3674.8	1095.5	93.9	54	77	23
	ET_ei_05_n1	0.556	0	20	0	1	0	25	30	MTBE	0	1346.5	358.5	91.4	58	79	21
	ET_ucb_2_n1	0.556	0	20	0	1	0	37.5	30	MTBE	0	391.1	259.2	82.7	20	60	40
	MLP_ei_05_n1	5	0	20	0	1	0	0	30	TFE	0	3736.3	1411.9	93.0	45	73	27
	MLP_ucb_2_n1	5	0	20	0	0	0	0	10	formamide	1553.6	437.6	925.9	46.5	-36	32	68
	GPR_ei_05_n2	0	5	6	1	0	1	12.5	30	2,4-dimethyl-3-pentanone	493.4	151.3	793.3	59.9	-68	16	84
	GPR_ucb_2_n2	0	5	6	1	0	1	12.5	30	Methyl linolenate	454.7	212.8	234.2	36.1	-5	48	52
	RFR_ei_05_n2	0	5	1	0	0	0	12.5	30	1-decanol	512.6	9.1	615.1	42.9	-97	1	99
	RFR_ucb_2_n2	0	5	1	0	0	1	12.5	30	1-decanol	656.1	4.7	655.9	39.9	-99	1	99
	ET_ei_05_n2	0	5	4	1	0	0	12.5	30	1-decanol	355.3	15.6	1900.5	67.2	-98	1	99
	ET_ucb_2_n2	0	5	4	0	0	0	12.5	30	1-decanol	194.9	3.9	1309.8	72.1	-99	0	100
		0	*5*	*13*	*1*	*0*	*0*	*0*	*10*	*tributylamine*	*20.9*	*0*	*707.7*	***94.6***	***-100***	***0***	***100***
	MLP_ucb_2_n2	0	5	20	1	1	1	0	30	tributylamine	51	0	522.5	77.9	-100	0	100
**Pred.**	N1_pred_best_dist	0	5	4	1	0	1	12.5	30	1-decanol	294.1	0	752.8	57.1	-100	0	100
	N2_pred_best_dist	1.667	0	19	0	0	0	12.5	23.333	MTBE	0	1327.2	693.6	92.2	31	66	34
	N2_pred_best	2.778	0	19	0	0	0	0	10	N,N-Dimethyloctanamide		2054.1	291.4	97.5	75	88	12
	N1_pred_best	0	5	19	0	0	0	12.5	30	MTBE	193.2	23.1	2422.6	91.4	-98	1	99
	N1_pred_best_dist_0	*0*	*0.556*	*14*	*1*	*0*	*0*	*0*	*10*	*tributylamine*	*3.5*	*456*	*3958.3*	***99.7***	***-79***	***10***	***90***
	N2_pred_best_dist_0	*0*	*0*	*20*	*0*	*0*	*0*	*12.5*	*30*	*MTBE*	*202.1*	*1475*	*2.9*	***86.0***	***100***	***100***	***0***

UPBO Benchmarks

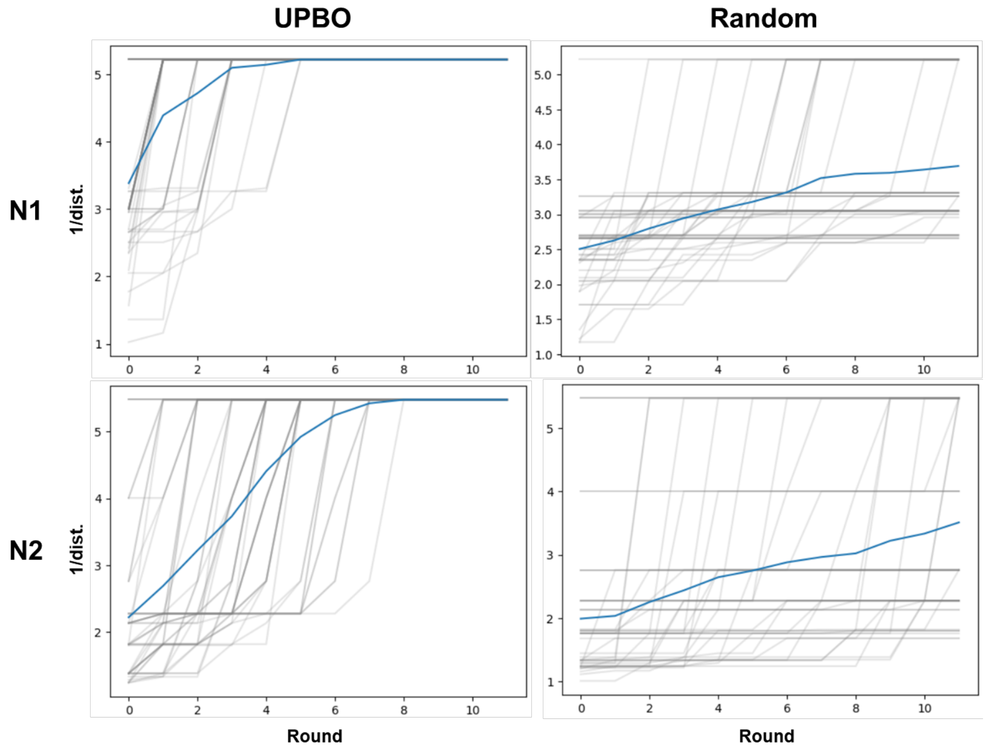

Figure SI-6: Benchmark of UPBO algorithm against random chance. The running maximum is plotted against each round. Average of all runs in blue, all individual runs in gray.

In order to benchmark our algorithm, we performed a test against random chance based on the experimental data set. A random set of 12 reactions were chosen from the experimental data set as a starting point. 12 rounds of Bayesian optimization were performed, with the experimental data set as the full search space. This procedure was repeated 50 times, and the average progression over time was plotted above. While random chance would occasionally find the best condition, on average random chance was unable to find the max value. In contrast, for either N1 or N2 isomers the UPBO algorithm was able to achieve the best possible result in 8 rounds or less.

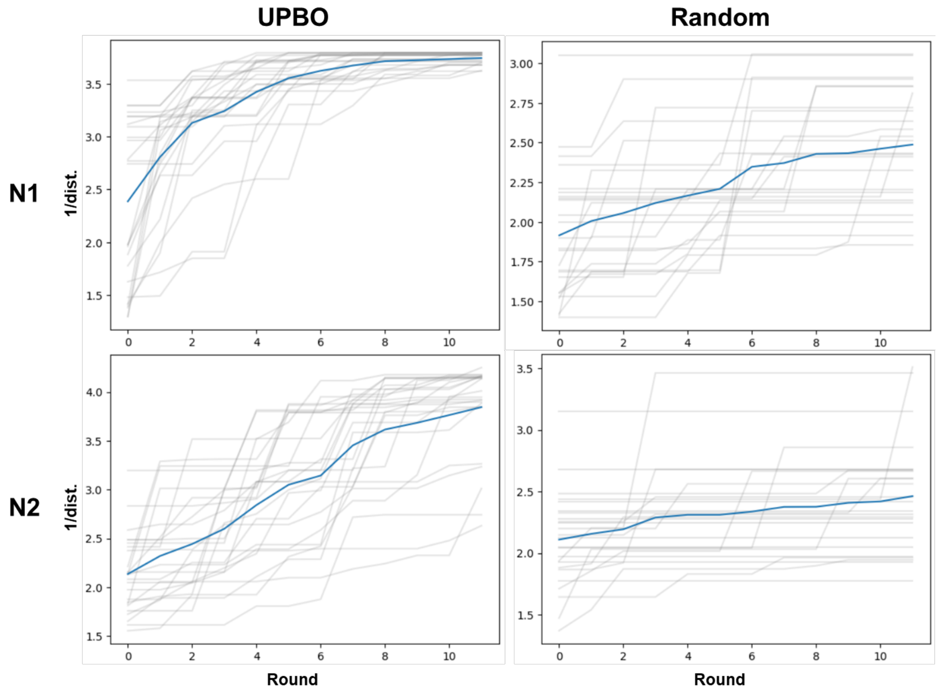

Figure SI-7 Benchmark of UPBO algorithm against random chance. Models were tested on the full search space, with values predicted by an average of three regressors. The running maximum is plotted against each round. Average of all runs in blue, all individual runs in gray.

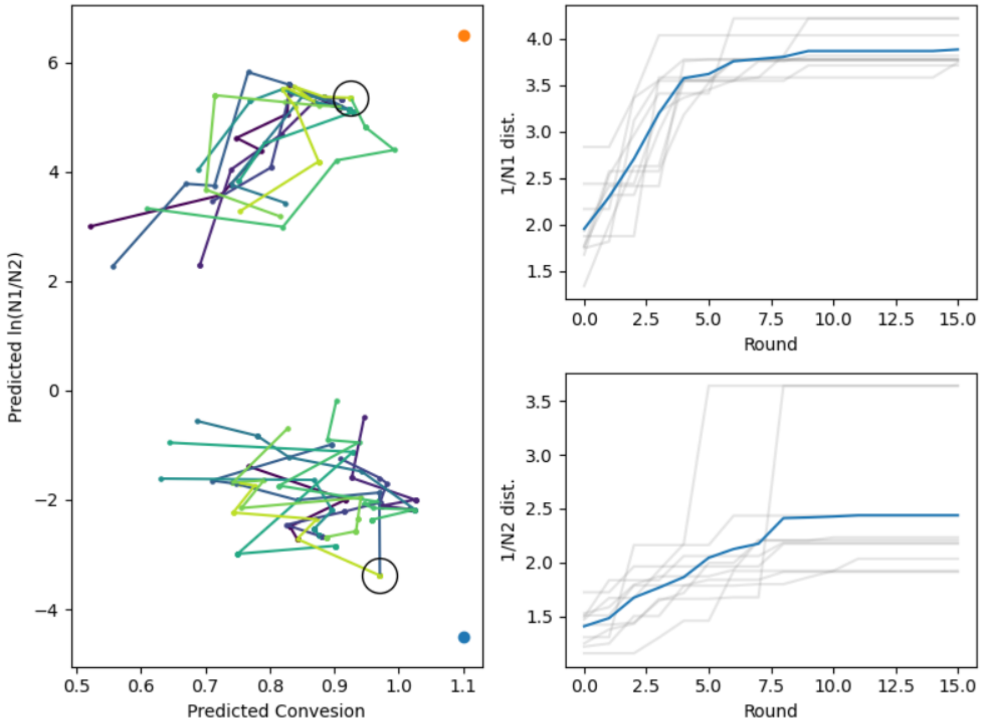

Figure SI-8: Plot showing progression in 2 dimensions for the predicted search space. Plot on the left shows the progression for 10 repeat runs, color coded by run. The running maximum for each round is plotted, showing steady progression towards the utopia point (red, blue dots) for the respective isomer. The plot on the right shows the running maximum for the inverse distance metric for each isomer. The black circles represents the best overall value in the dataset depending on the isomer.

As the experimental set was biased in favor of desirable conditions, we also wanted to check against a broader search space. In order to achieve this, yield and selectivity values were predicted for the whole search space using a voting regressor from Scikit-Learn, using a random forest, Gaussian process, and neural network based regressor. This surrogate data set was used to benchmark the UPBO algorithm against random chance. The UPBO algorithm is able to show improvement round over round even in this larger search space, though it does not always converge to the global maximum in the allotted number of iterations.

Feature Importance

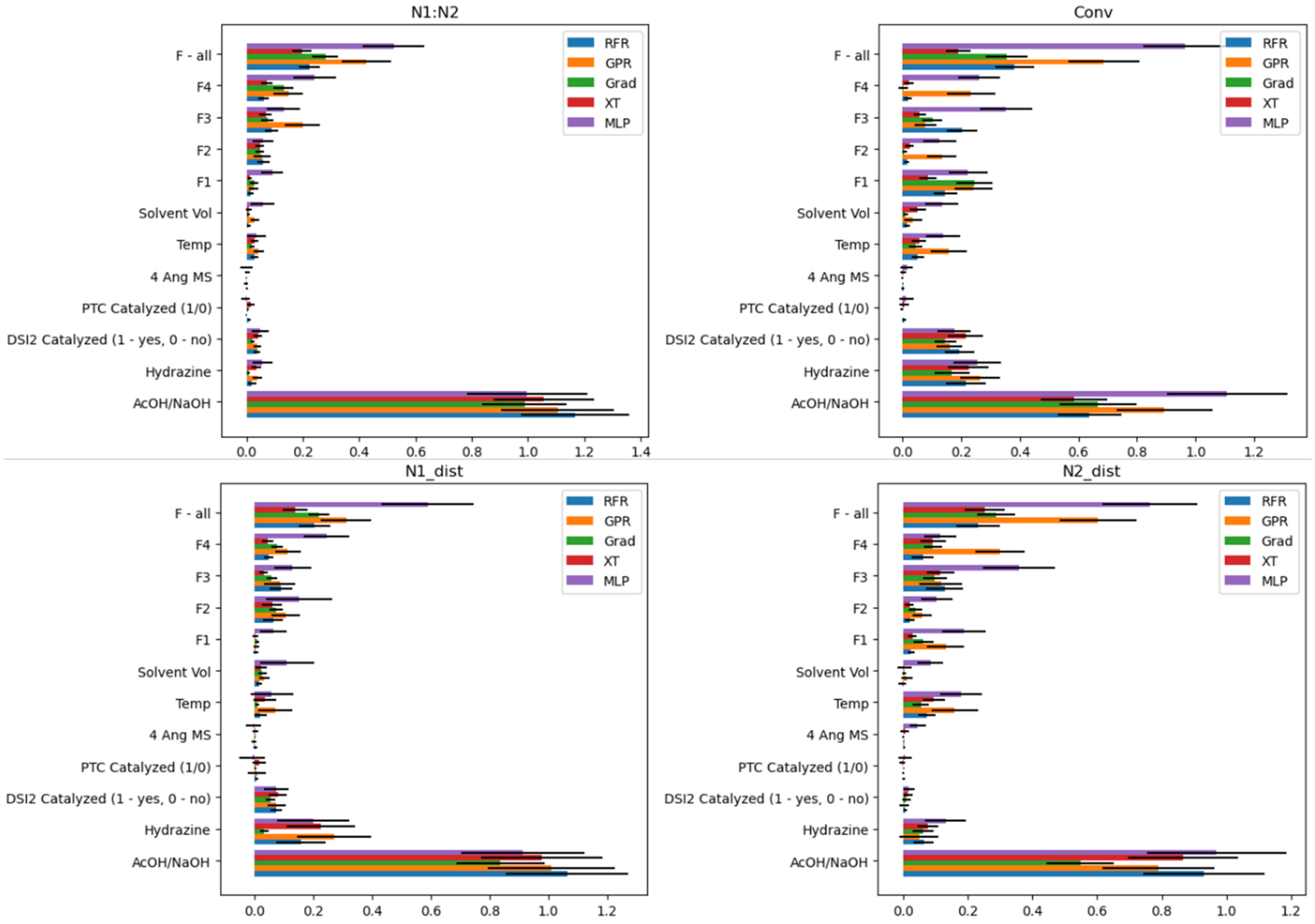

Figure SI-9: Feature importances for models used in this study.

Compound Characterization

The following compounds have been previously reported and the reported information was found to be consistent. References have been provided to the original disclosure when possible. The following compounds are commercially available **1a**, **1b**, **1c**, **1d**, **1e** and **1f**. The following N1-methyl pyrazoles have been reported: **3a**[1], **3b**[2], **3c**[3], **3d**[4], **3e**[5], **3f**[5] and **3g**[6]. The following N2-methyl pyrazoles have been reported: **4a**[7], **4b**[2], **4c**[3,8], **4d**[9], **4f**[10]. **4e** has not been reported and its characterization data is shown below.

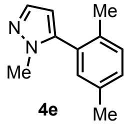

4e

5-(2,5-dimethylphenyl)-1-methyl-1*H*-pyrazole on 250 mg **1e** scale. General procedure provided **4e** as a clear colorless oil in 81% isolated yield (0.177 g) with 98% assay yield. **^{1}H-NMR** (400 MHz, $CDCl_3$) δ 7.55 – 7.50 (m, 1H), 7.22 – 7.12 (m, 2H), 7.02 (d, J = 1.8 Hz, 1H), 6.20 – 6.15 (m, 1H), 3.65 (d, J = 0.9 Hz, 3H), 2.34 (s, 3H), 2.11 (s, 3H). **^{13}C-NMR** (101 MHz, $CDCl_3$) δ 142.70, 138.40, 135.28, 134.35, 130.86, 130.17, 129.79, 128.28, 106.27, 36.60, 20.83, 19.29. **HRMS** calc. (m/z, m+1) 187.1235, found (m/z, m+1)

[1] a) ELI LILLY & CO - WO2005/80380, 2005, A1 Location in patent: Page/Page column 40-41 b) Yamamoto, T.; Togo, H. Transformation of Arenes into 3-Arylpyrazoles and 3-Arylisoxazolines with β-Bromopropionyl Chloride, Hydrazine, and Hydroxylamine. *Tetrahedron* **2020**, 76 (8), 130920. https://doi.org/10.1016/j.tet.2019.130920. c) Kankala, S.; Rama, K. R.; Kesari, C.; Björkling, F.; Nerella, S.; Gundepaka, P.; Guguloth, H.; Thota, N. Synthesis of Novel Fluorophenylpyrazole-Picolinamide Derivatives and Determination of Their Anticancer Activity. *Synthetic Commun* **2020**, 50 (19), 2997–3006. https://doi.org/10.1080/00397911.2020.1791341.

[2] Elguero, J.; Marzin, C.; Roberts, J. D. Carbon-13 Magnetic Resonance Studies of Azoles. Tautomerism, Shift Reagent Effects, and Solvent Effects. *J Org Chem* **1974**, 39 (3), 357–363. https://doi.org/10.1021/jo00917a017.

[3] Giudice, M. R. D.; Mustazza, C.; Borioni, A.; Gatta, F.; Tayebati, K.; Amenta, F.; Tucci, P.; Pieretti, S. Synthesis of 1-Methyl-5-(Pyrazol-3- and -5-yl- and 1, 2, 4-triazol-3- and 5-yl)-1, 2, 3, 6-tetrahydropyridine Derivatives and Their Evaluation as Muscarinic Receptor Ligands. *Arch. Pharm. Pharm. Med. Chem.* **2003**, 336 (3), 143–154. https://doi.org/10.1002/ardp.200390013.

[4] Hao, L.; Liu, H.; Zhang, Z.; Wen, F.; Xia, C.; Pang, Z. Cascade Reaction to 1H-Pyrazoles from Hydrazones via Sodium Ni-Trite Promoted Dual C–C/C–N Formation, Annulation and Aromatization with 1,2-Dichloroethane. *Chinese Chem Lett* **2021**, 32 (7), 2309–2312. https://doi.org/10.1016/j.cclet.2021.02.025.

[5] Yamamoto, T.; Togo, H. Transformation of Arenes into 3-Arylpyrazoles and 3-Arylisoxazolines with β-Bromopropionyl Chloride, Hydrazine, and Hydroxylamine. *Tetrahedron* **2020**, 76 (8), 130920. https://doi.org/10.1016/j.tet.2019.130920.

[6] a) Bayer AG - US2013/281455, 2013, A1. b) Bayer AG - US2011/183978, 2011, A1.

[7] Hackenberger, D.; Weber, P.; Blakemore, D. C.; Goossen, L. J. Synthesis of 3-Substituted 2-Arylpyridines via Cu/Pd-Catalyzed Decarboxylative Cross-Coupling of Picolinic Acids with (Hetero)Aryl Halides. *J Org Chem* **2017**, 82 (7), 3917–3925. https://doi.org/10.1021/acs.joc.7b00046.

[8] Levy, J. N.; Alegre-Requena, J. V.; Liu, R.; Paton, R. S.; McNally, A. Selective Halogenation of Pyridines Using Designed Phosphine Reagents. *J Am Chem Soc* **2020**, 142 (25), 11295–11305. https://doi.org/10.1021/jacs.0c04674.

[9] Mateos, C.; Mendiola, J.; Carpintero, M.; Mínguez, J. M. Regioselective Palladium-Catalyzed Arylation of 4-Chloropyrazoles. *Org Lett* **2010**, 12 (21), 4924–4927. https://doi.org/10.1021/ol1020898.

[10] Beladhria, A.; Beydoun, K.; Ammar, H.; Salem, R.; Doucet, H. Pd-Catalysed Direct 5-Arylation of 1-Methylpyrazole with Aryl Bromides. *Synthesis* **2011**, 2011 (16), 2553–2560. https://doi.org/10.1055/s-0030-1260076.

187.1228. **IR** (cm^{-1}) 2945, 2920, 1493, 1456, 1420, 1389, 1328, 1276, 1238, 1192, 1165, 1136, 1063, 1037, 998, 971, 889, 860, 812, 779, 749, 705, 674, 648, 576.

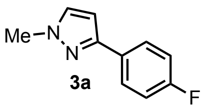

3-(4-fluorophenyl)-1-methyl-1H-pyrazole on 100 mg (**1a**, 0.50 mmol) scale. General procedure provided **3a** as a white solid in 84% isolated yield (0.0745 g, 0.423 mmol) with 94% assay yield.

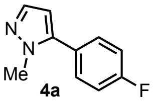

5-(4-fluorophenyl)-1-methyl-1H-pyrazole on 100 mg (**1a**, 0.50 mmol) scale. General procedure provided **4a** as a white solid in 85% isolated yield (0.0756 g, 0.429 mmol) with 98% assay yield.

Pyrazole Cyclization Reaction Profiles

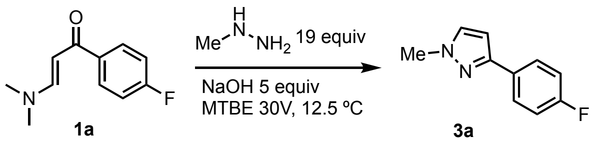

Table SI-3. N1 Selective reaction conditions with **1a**.

40	Rxn Number	AcOH	NaOH (equiv 50% Aq NaOH)	Hydrazine (equiv)	DSI2 Catalyzed 10mol% (1/0 = yes/no)	PTC Catalyzed (1/0)	4 Ang MS (1:1 g/g MS:SM)	Temp	Solvent Vol	Solvent Name
1	N1_pred_best	0	5	19	0	0	0	12.5	30	MTBE

Table SI-4. Results of N1 Selective Reaction Profile with **1a**.

Time min	SM A%	Pyr. A%	N2 A%	N1 A%	N2'	N1'	Prod. Formation	SM Consumption	N2'-N1'
0	97.73	0.00	0.25	1.71	13	87	2	2	-75
5	88.24	0.00	0.56	10.38	5	95	11	12	-90
10	76.60	0.33	0.68	21.01	3	97	22	23	-94
30	42.29	0.60	0.79	54.38	1	99	55	58	-97
40	32.65	0.60	0.96	63.90	1	99	65	67	-97
50	26.51	0.61	0.74	69.99	1	99	71	73	-98
60	21.82	0.57	0.64	74.84	1	99	75	78	-98
80	16.15	0.55	0.79	80.38	1	99	81	84	-98
100	12.65	0.41	1.49	83.48	2	98	85	87	-96
120	10.09	0.30	0.80	87.23	1	99	88	90	-98
150	8.07	0.17	0.44	89.77	0	100	90	92	-99
180	7.04	0.13	0.38	90.61	0	100	91	93	-99
210	6.44	0.15	0.56	91.48	1	99	92	94	-99
240	6.16	0.19	0.72	91.62	1	99	92	94	-98
300	5.92	0.18	0.60	92.18	1	99	93	94	-99
360	5.79	0.27	0.28	92.30	0	100	93	94	-99
540	5.80	0.31	0.85	90.93	1	99	92	94	-98
720	5.80	0.36	0.59	91.58	1	99	92	94	-99
1080	5.81	0.18	0.18	91.81	0	100	92	94	-100

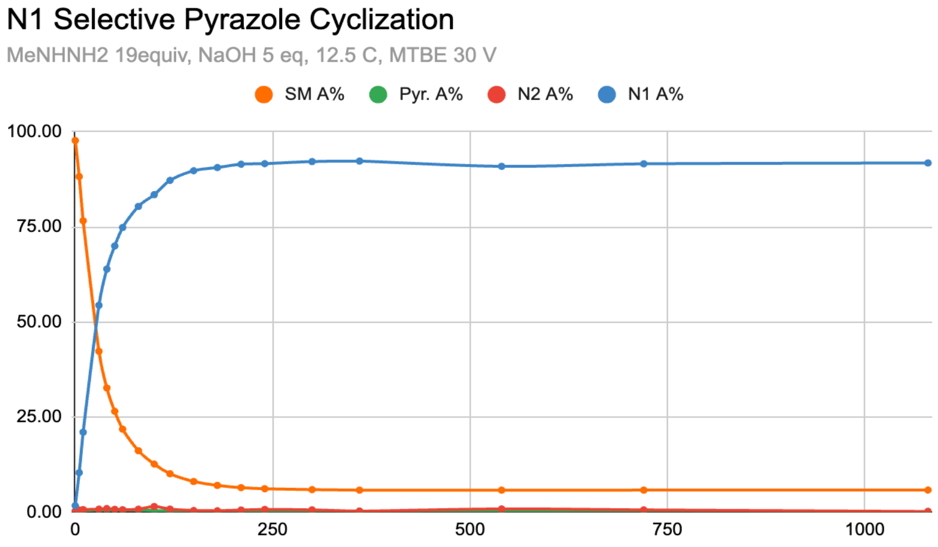

Figure SI-10. Graph of N1 selective reaction profile with vinylogous amide **1a**.

N2-Selective pyrazole cyclization

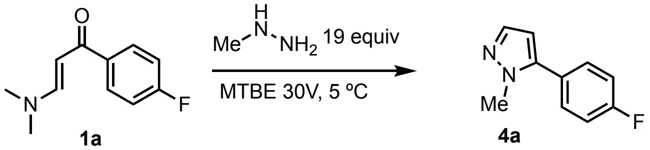

1a **4a**

Table SI-5. N2 selective reaction conditions with **1a**.

40	Rxn Number	AcOH	NaOH (equiv 50% Aq NaOH)	Hydrazine (equiv)	DSI2 Catalyzed 10mol% (1/0 = yes/no)	PTC Catalyzed (1/0)	4 Ang MS (1:1 g/g MS:SM)	Temp	Solvent Vol	Solvent Name
1	N2_pred_best	0	0	20	0	0	0	5	30	MTBE

Table SI-6. Results of N2 Selective Reaction Profile with **1a**

Time (min)	SM A%	HN-Pyr. A%	N2 A%	N1 A%	N2'	N1'	Prod. Formation	SM Consumption	N2'-N1'
0	66.96	3.21	25.01	4.82	84	16	30	33	68
5	61.73	4.01	29.02	5.25	85	15	34	38	69
10	57.75	4.66	31.87	5.72	85	15	38	42	70
20	50.30	6.36	36.77	6.57	85	15	43	50	70
30	46.01	7.90	39.44	6.65	86	14	46	54	71
40	43.48	8.58	41.25	6.69	86	14	48	57	72
50	37.66	10.44	45.32	6.59	87	13	52	62	75
60	32.85	11.68	48.70	6.78	88	12	55	67	76
80	29.84	12.91	50.74	6.51	89	11	57	70	77
100	24.08	15.14	54.40	6.38	89	11	61	76	79
120	21.00	15.82	56.96	6.22	90	10	63	79	80
150	16.95	16.81	60.68	5.56	92	8	66	83	83
180	13.42	17.33	64.34	4.91	93	7	69	87	86
300	3.43	16.02	77.26	3.28	96	4	81	97	92
360	2.63	15.63	78.86	2.88	96	4	82	97	93
540	0.95	12.92	83.87	2.26	97	3	86	99	95
720	0.42	10.55	86.91	2.13	98	2	89	100	95
1080	0.17	6.67	90.95	2.21	98	2	93	100	95

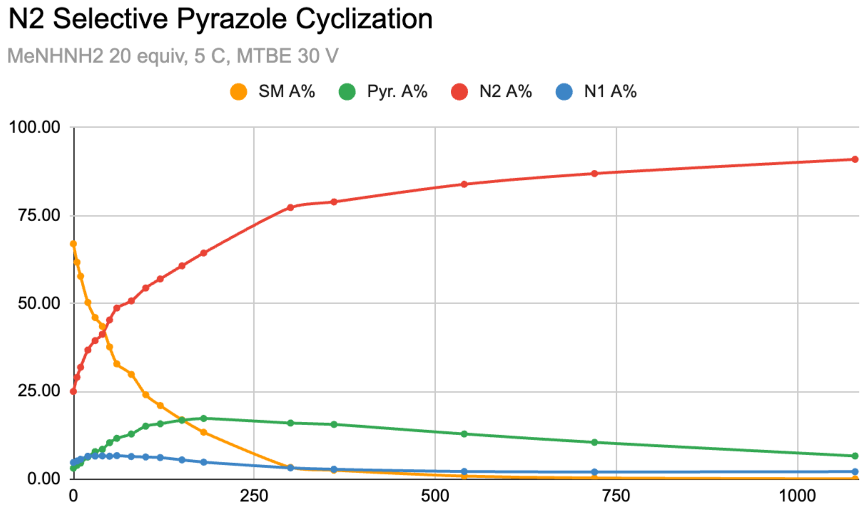

Figure SI-11. Graph of N2 selective reaction profile with **1a**

Pyrazole Cyclization Control Reactions

N1 pyrazole subjected to N2 cyclization conditions

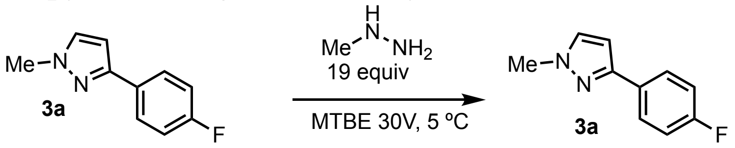

Table SI-7. Conditions for reaction of **3a** under N2 cyclization conditions.

44A	Rxn Number	AcOH	NaOH (equiv 50% Aq NaOH)	Hydrazine (equiv)	DSI2 Catalyzed 10mol% (1/0 = yes/no)	PTC Catalyzed (1/0)	4 Ang MS (1/1 Ms w/ VA)	Temp	Solvent Vol	Solvent Name
R	control	0	0	19	0	0	0	12.5	30	MTBE

Table SI-8. Results of control reaction in which 3a is subjected to N2 cyclization conditions.

TIme min	SM A%	Pyr. A%	N2 A%	N1 A%	N2'	N1'	Conv to P	SM Consumption	N2'-N1'
0	2.93	4.15	0.82	92.10	1	99	93	97	-98
5	0.89	1.84	0.88	96.38	1	99	97	99	-98
10	1.00	1.76	1.04	96.20	1	99	97	99	-98
20	1.18	1.71	0.96	96.15	1	99	97	99	-98
30	1.01	1.93	0.90	96.15	1	99	97	99	-98
40	1.03	1.85	0.98	96.14	1	99	97	99	-98
50	1.54	2.48	1.01	94.96	1	99	96	98	-98
60	3.49	4.85	1.20	90.45	1	99	92	97	-97
80	1.69	2.62	0.99	93.10	1	99	94	98	-98
100	1.04	1.88	0.90	93.82	1	99	95	99	-98
120	1.05	1.91	0.99	94.49	1	99	95	99	-98
180	0.90	1.85	1.07	95.36	1	99	96	99	-98
240	1.01	2.81	0.87	95.30	1	99	96	99	-98
480	0.93	4.78	0.89	93.40	1	99	94	99	-98
960	1.27	6.40	0.88	91.46	1	99	92	99	-98

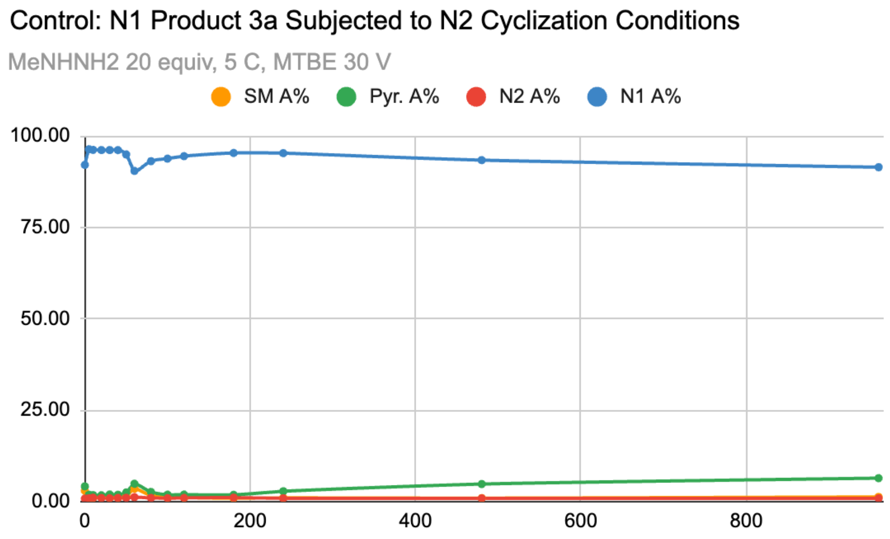

Figure SI-12. Graphs of reaction profile for product 3a under N2 condensation conditions.

N2 pyrazole subjected to N1 cyclization conditions

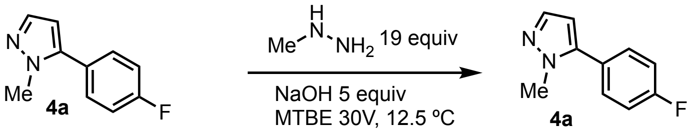

Table SI-9. Conditions for reaction of **4a** under N2 cyclization conditions.

44A	Rxn Number	AcOH	NaOH (equiv 50% Aq NaOH)	Hydrazine (equiv)	DSI2 Catalyzed 10mol% (1/0 = yes/no)	PTC Catalyzed (1/0)	4 Ang MS (1/1 Ms w/ VA)	Temp	Solvent Vol	Solvent Name
R	control	0	5	19	0	0	0	12.5	30	MTBE

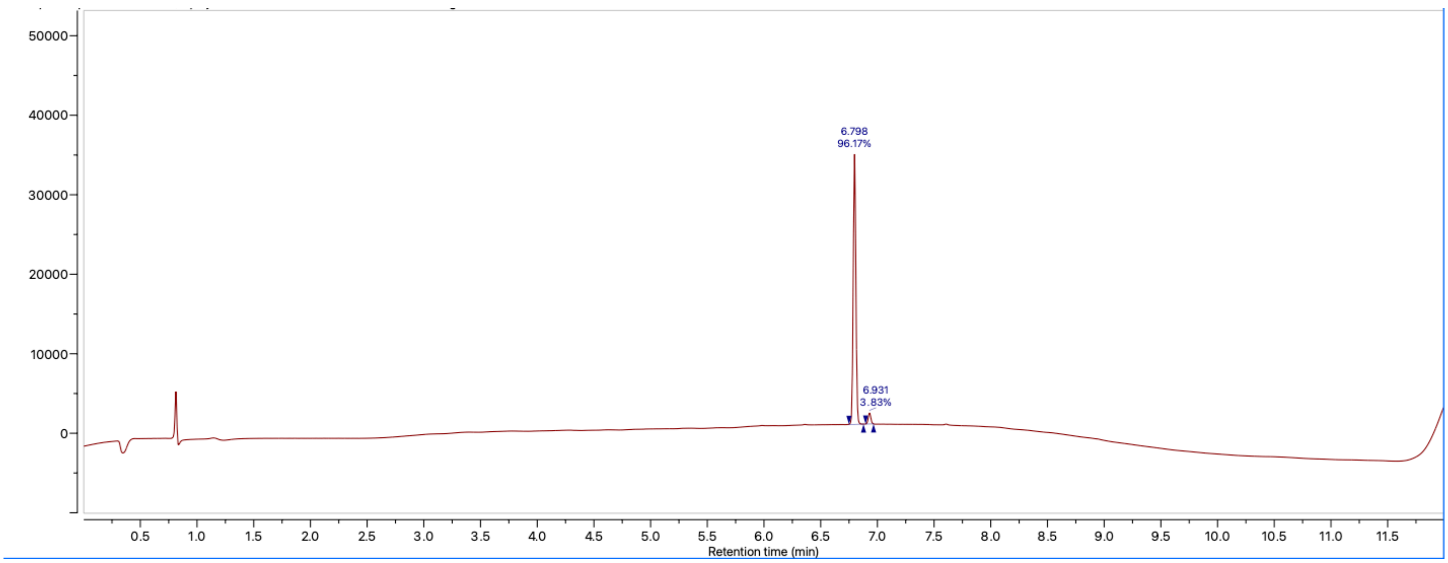

Figure SI-13. HPLC of product **4a** after 16h under N1 reaction conditions.

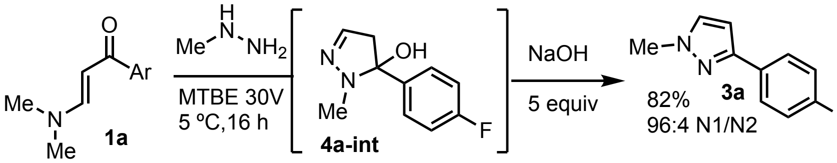

(7)

Table SI-10. Reaction conditions for formation of **4a-int** and addition of NaOH.

44C	Rxn Number	AcOH	NaOH (equiv 50% Aq NaOH)	Hydrazine (equiv)	DSI2 Catalyzed 10mol% (1/0 = yes/no)	PTC Catalyzed (1/0)	4 Ang MS (1/1 Ms w/ VA)	Temp	Solvent Vol	Solvent Name
1	control	0	5	19	0	0	0	12.5	30	MTBE

Table SI-11. HPLC Results of N2 pyrazole **4a** under N1 selective conditions

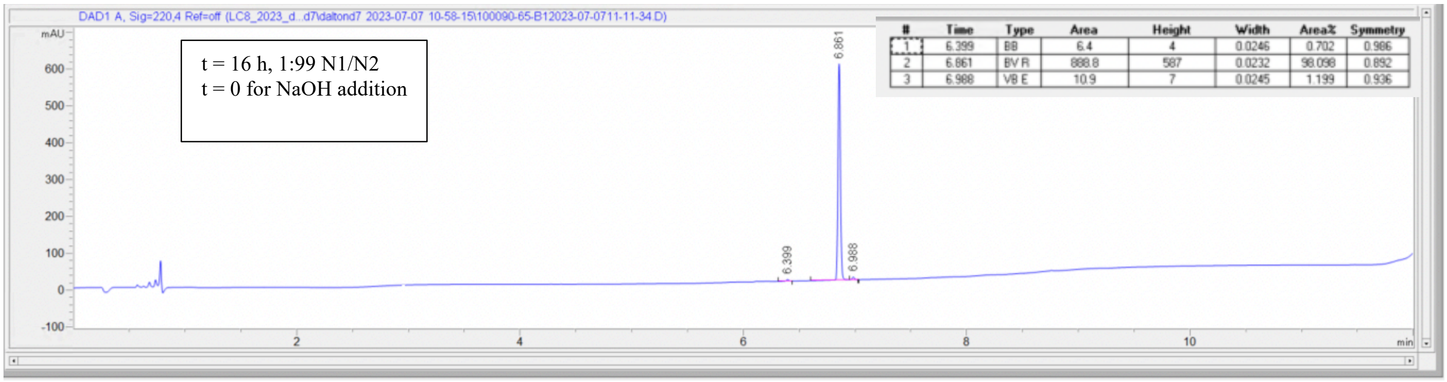

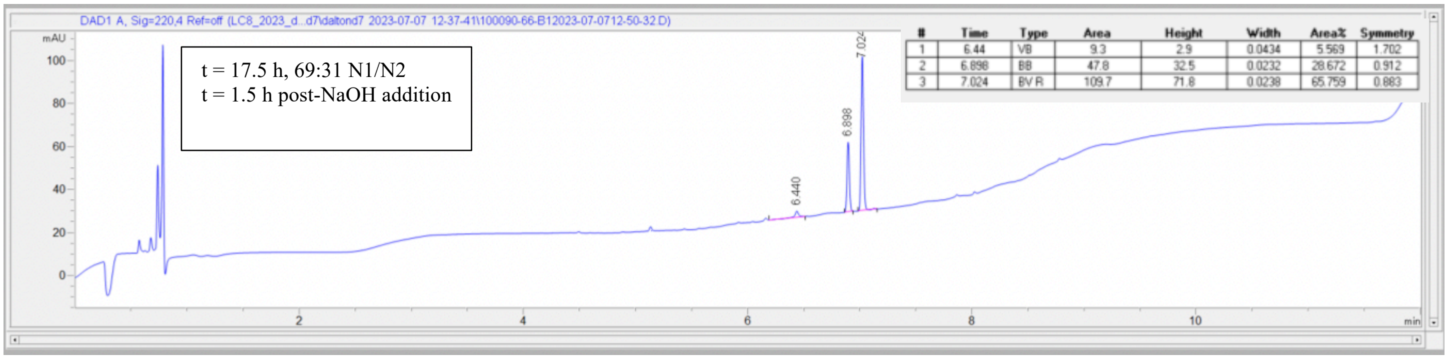

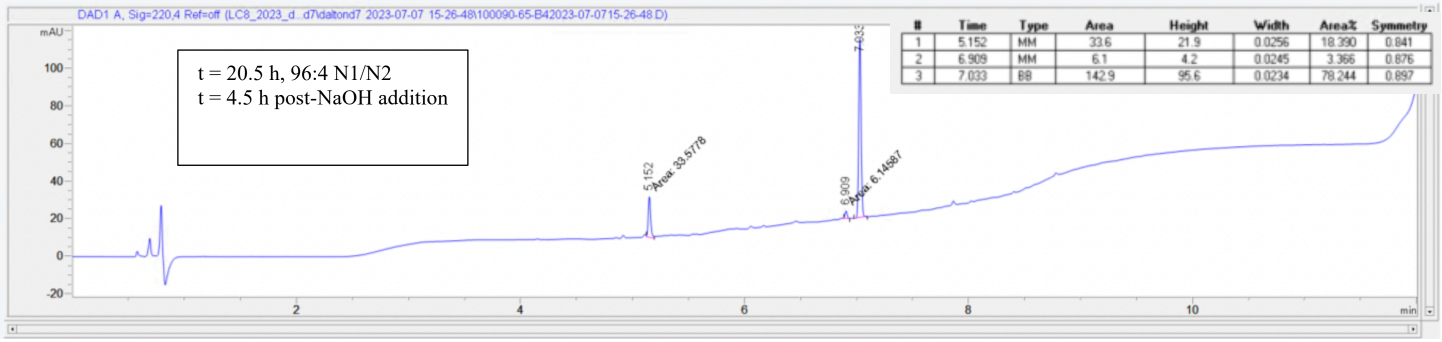

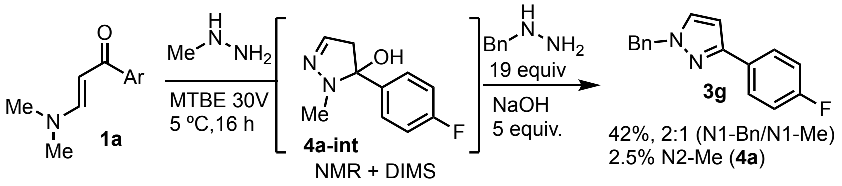

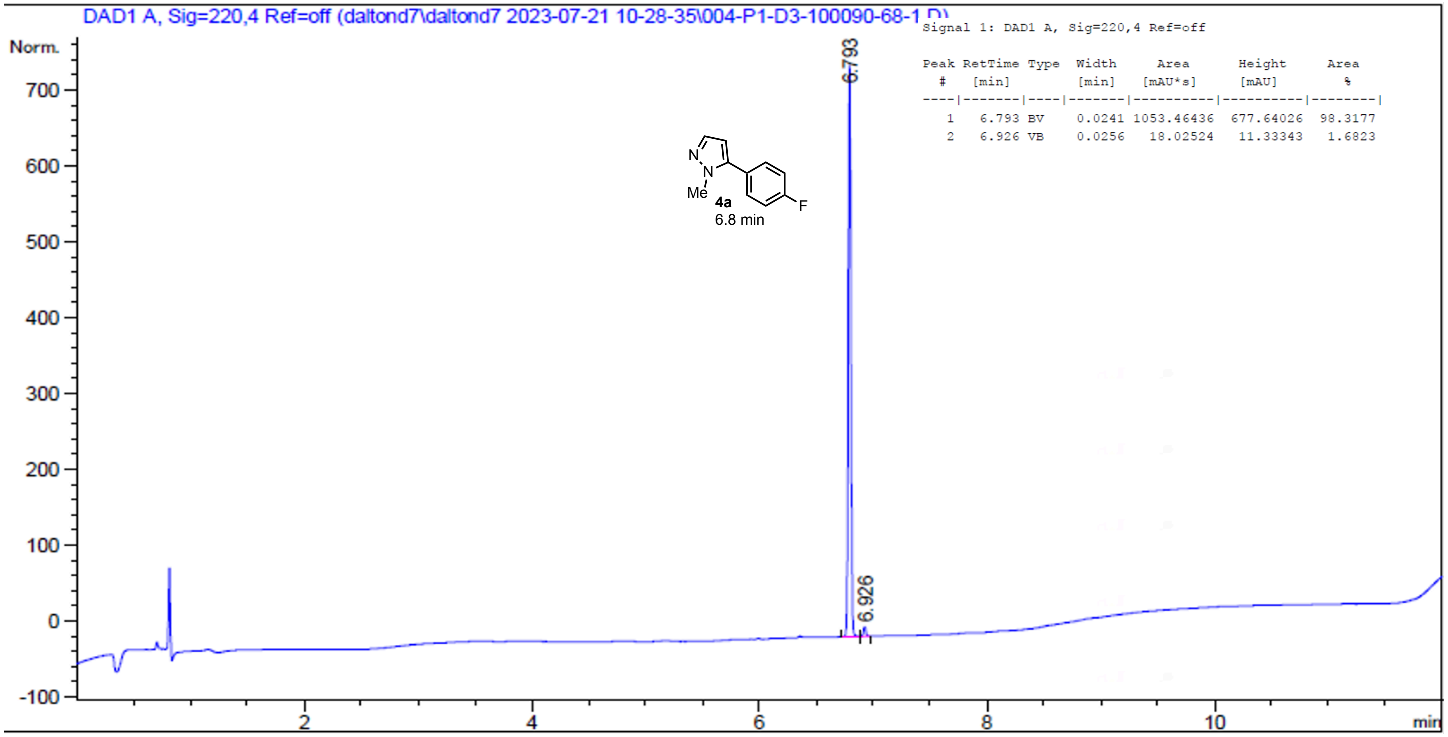

Figure SI-14. HPLC spectrum of reaction of 1a under N2 cyclization conditions at 16h.

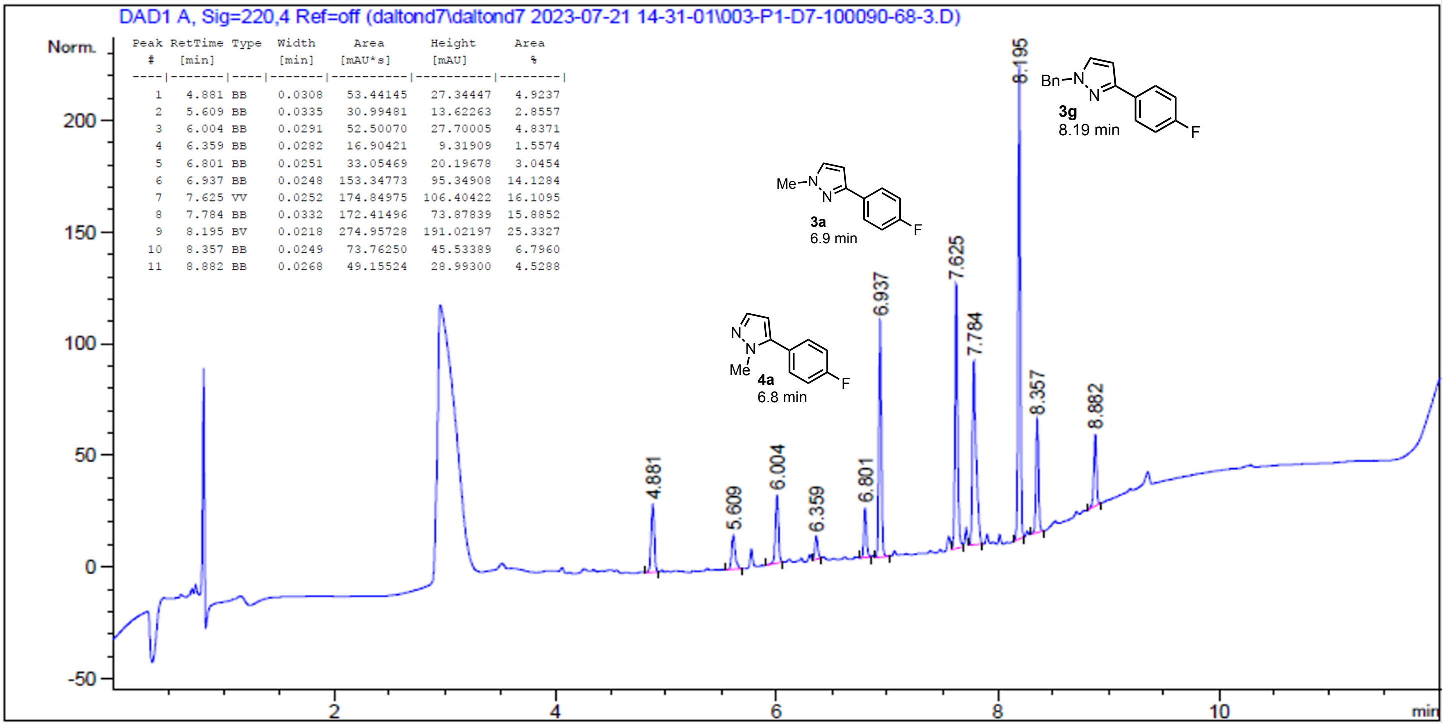

Figure SI-15. HPLC spectrum of reaction of 1a after addition of benzyl hydrazine (19 equiv) and NaOH (50% aq, 5 equiv)

NMR and MS Reaction Analysis of N2-selective conditions with Vinylogous Amide 1a.

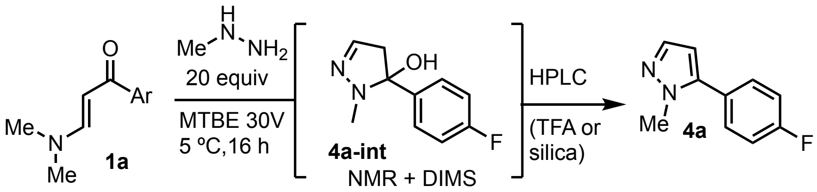

General procedure for N2 selective cyclization was followed with **1a** on 100 mg scale. After 16h at 5 C, an HPLC showed complete consumption of starting material **1a** and formation of **4a** product. A 50 uL aliquot of the reaction mixture was added to 500 uL of $CDCl_3$ and analyzed by ^{1}H- and ^{19}F-NMR. A 100 uL aliquot of the reaction mixture was rotovaped with a rotavary evaporator (14 mbar, 25 C bath T). A 100 uL aliquot of the reaction mixture was analyzed by qExactiv direct injection mass spectroscopy. The summation of the data suggests that the major intermediate is hemi-aminal **4a-int** above that rapidly dehydrates in the presence of acid to form N2-methyl pyrazole **4a**.

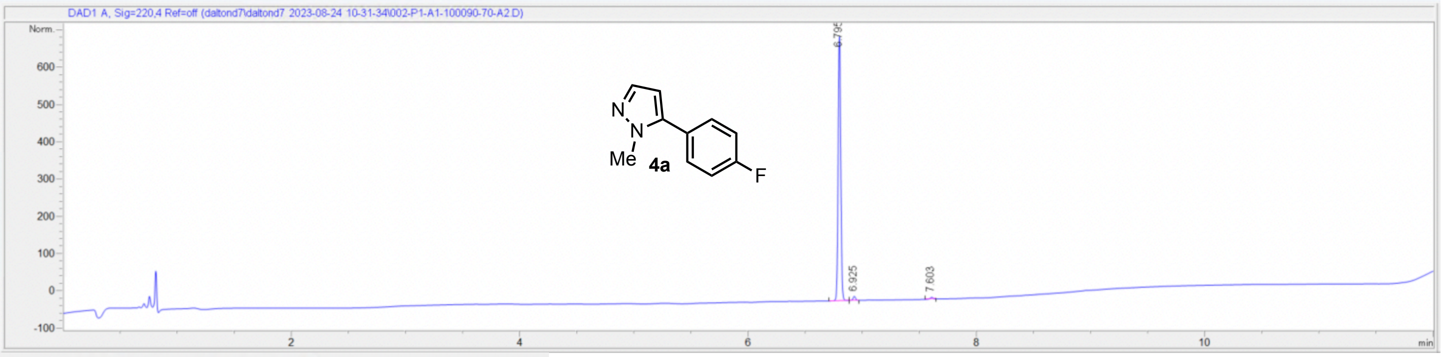

#	Time	Type	Area	Height	Width	Area%	Symmetry
1	6.795	BV	1015.7	637.6	0.0246	97.687	0.927
2	6.925	VB	16.6	10	0.0253	1.596	0.994
3	7.603	BB	7.5	4.4	0.0266	0.717	0.964

Figure SI-16. HPLC trace of reaction mixture after 16h. N2-isomer 4a is at RT = 6.79 and N1-isomer is at RT = 6.93 min.

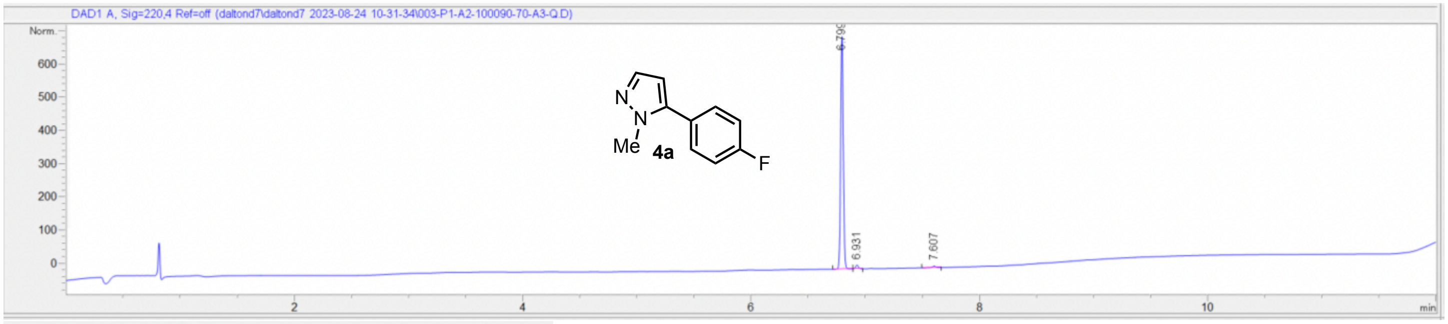

#	Time	Type	Area	Height	Width	Area%	Symmetry
1	6.799	BV	1008.9	631.7	0.0256	97.502	0.926
2	6.931	VB	16.9	10.3	0.0251	1.636	0.968
3	7.607	BB	8.9	4.7	0.028	0.861	1.047

Figure SI-17. HPLC trace of reaction mixture after addition of 1% TFA to the reaction mixture and stirring for 30 min.

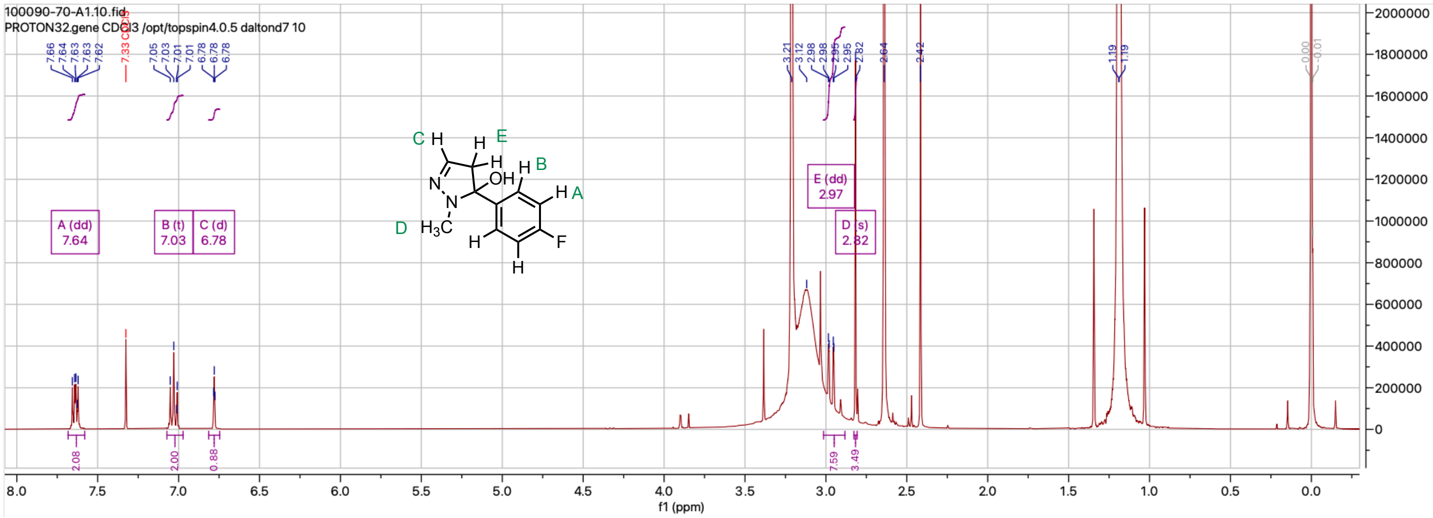

Figure SI-18. Direct ^{1}H-NMR spectrum of reaction mixture with no alteration ($CDCl_3$).

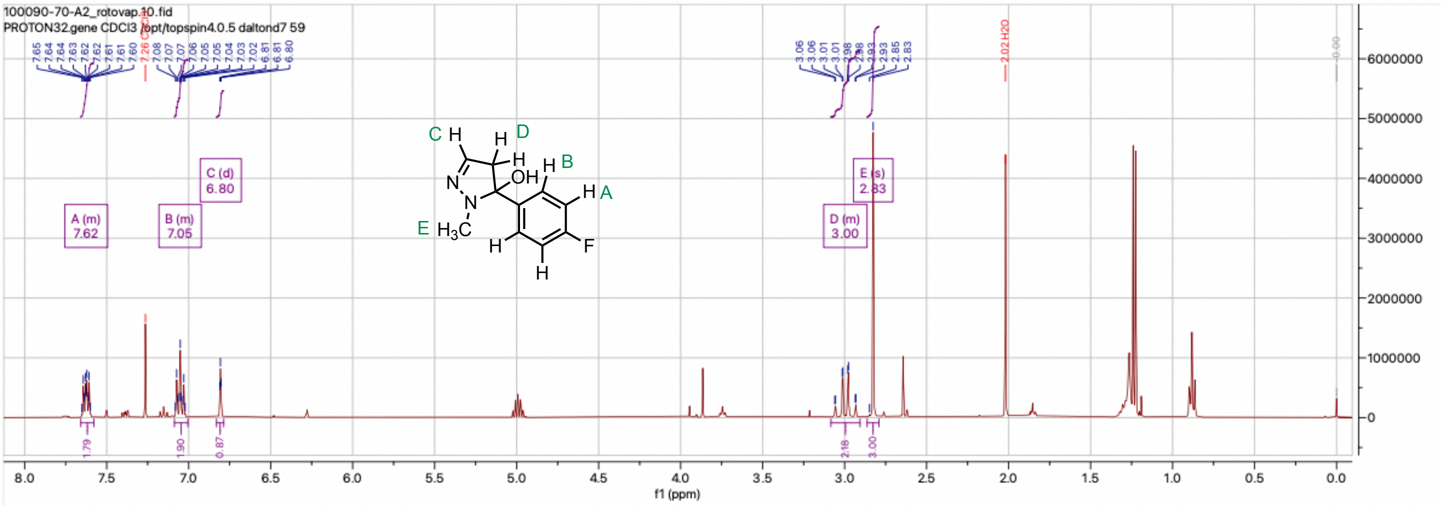

Figure SI-19. ^{1}H-NMR spectrum after rotary evaporation of the reaction mixture with 25 °C bath temp.

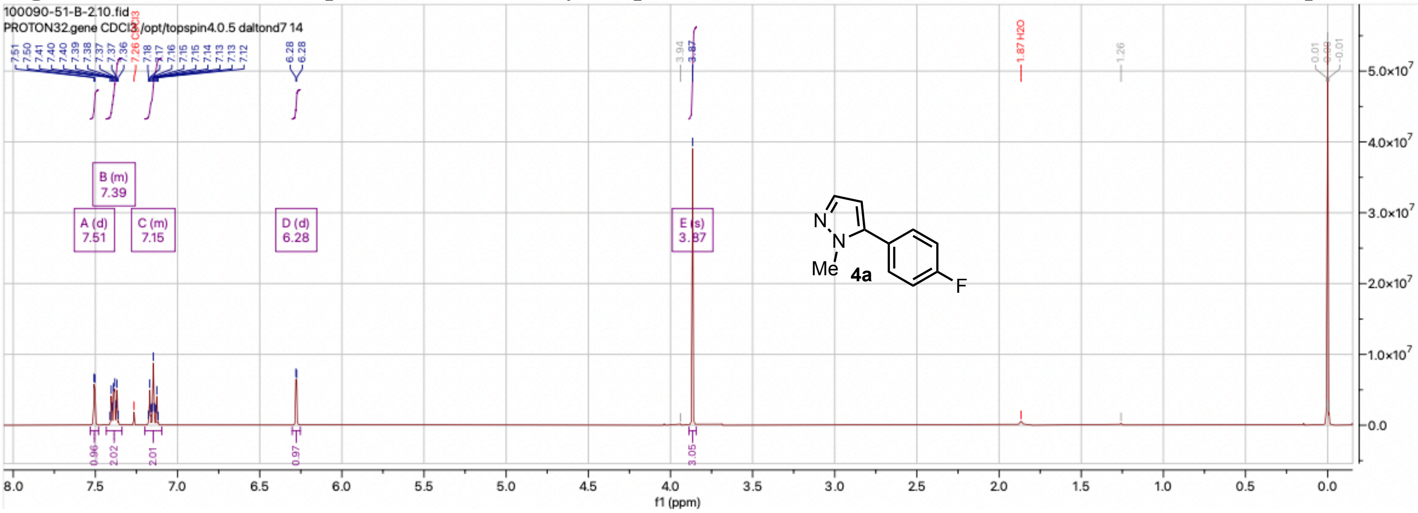

Figure SI-20. ^{1}H-NMR spectrum of isolated product.

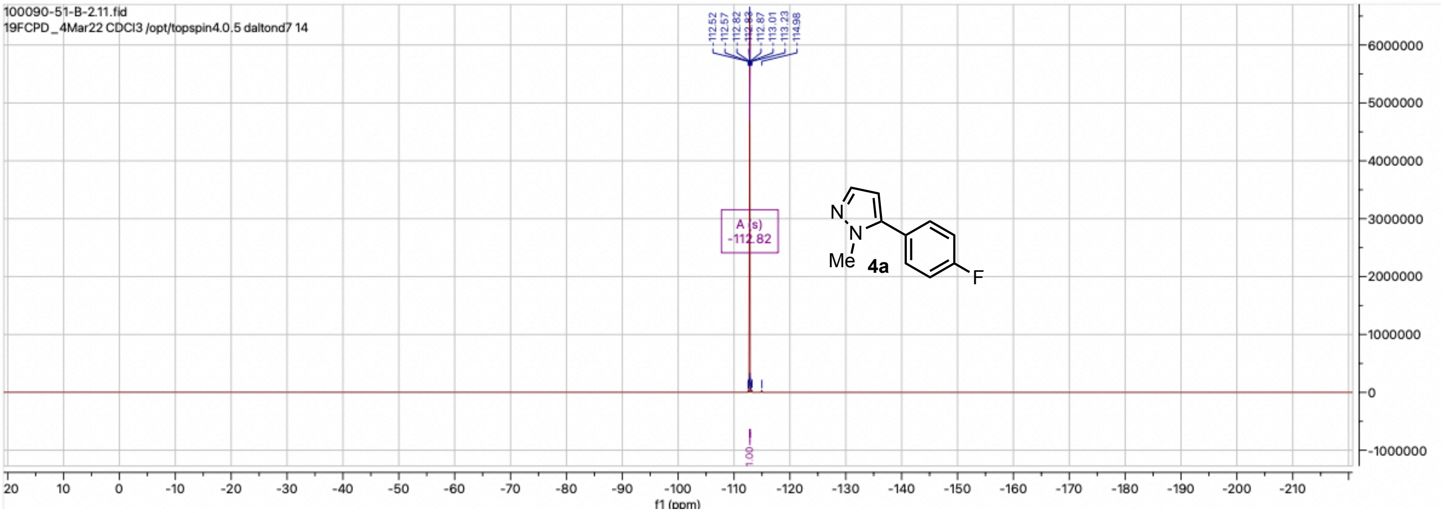

Figure SI-21. ^{19}F-NMR of purified N2-methylpyrazole **4a**.

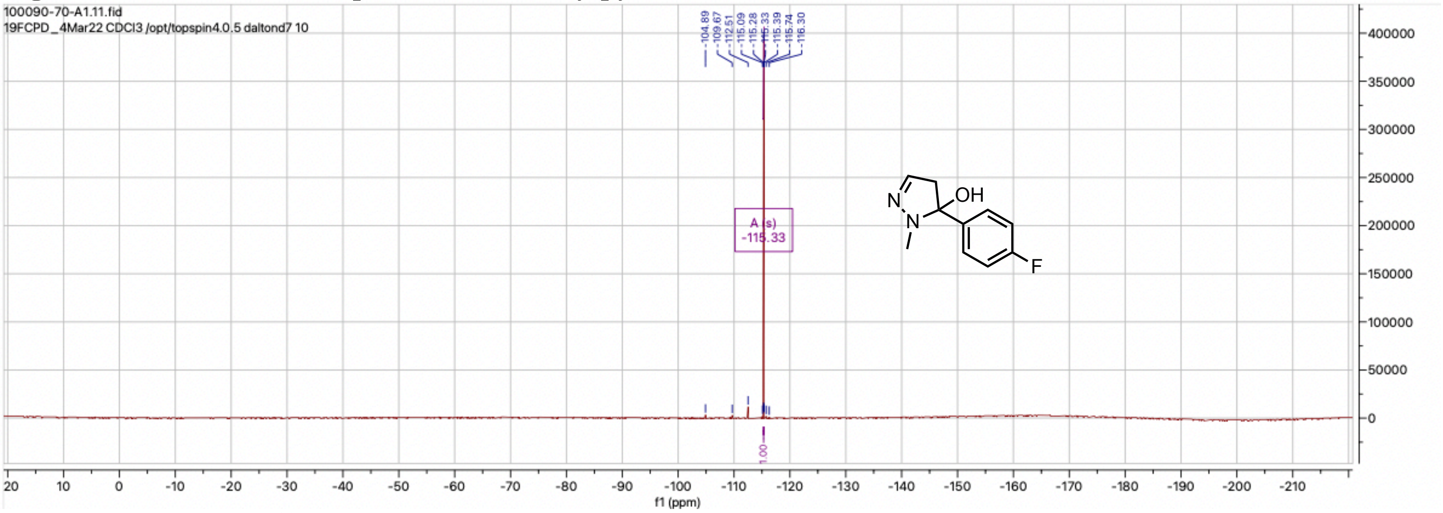

Figure SI-22. Direct ^{19}F-NMR spectrum of crude reaction mixture of **1a** under N2 favoring conditions.

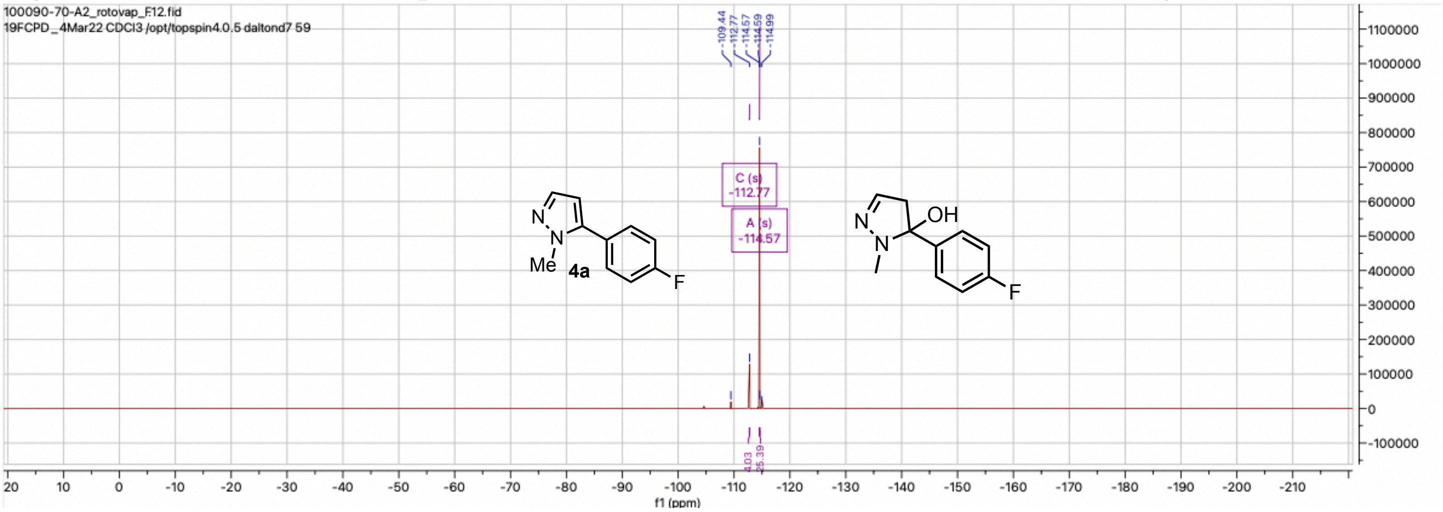

Figure SI-23. ^{19}F-NMR spectrum of rotavaped reaction mixture with **1a**.

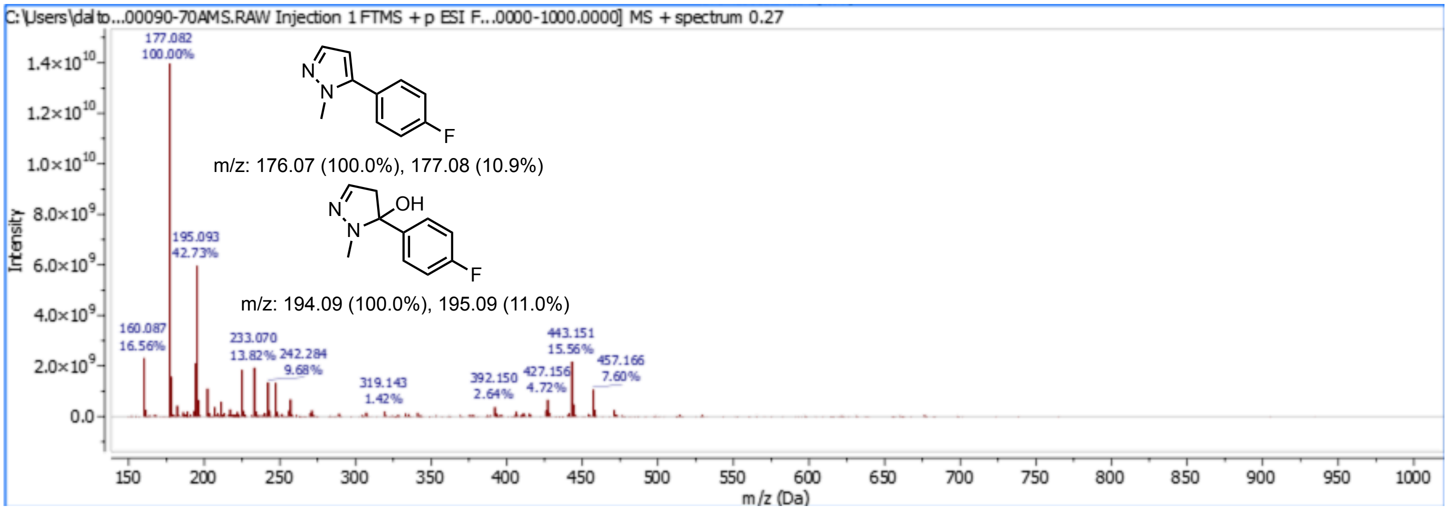

Figure SI-24. Direct-Injection Mass Spectrum of reaction mixture with **1a**.

MS Reaction Analysis of N1-selective conditions with Vinylogous Amide 1a.

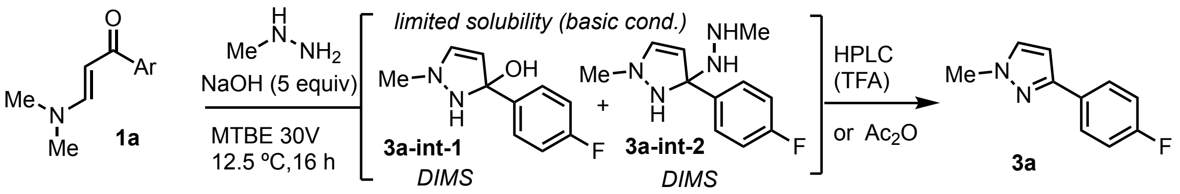

General procedure for N1 selective cyclization was followed with **1a** on 100 mg scale. After 16h at 12.5 C, an HPLC showed complete consumption of starting material **1a** and formation of **3a** product. A 100 uL aliquot of the reaction mixture was analyzed by qExactiv direct injection mass spectroscopy. The data suggests that the intermediate is a mixture of hemi-aminals **3a-int-1** and **3a-int-2** which have different solubilities. Both intermediates rapidly dehydrate in the presence of acid or acetic anhydride to form N2-methyl pyrazole **3a**.

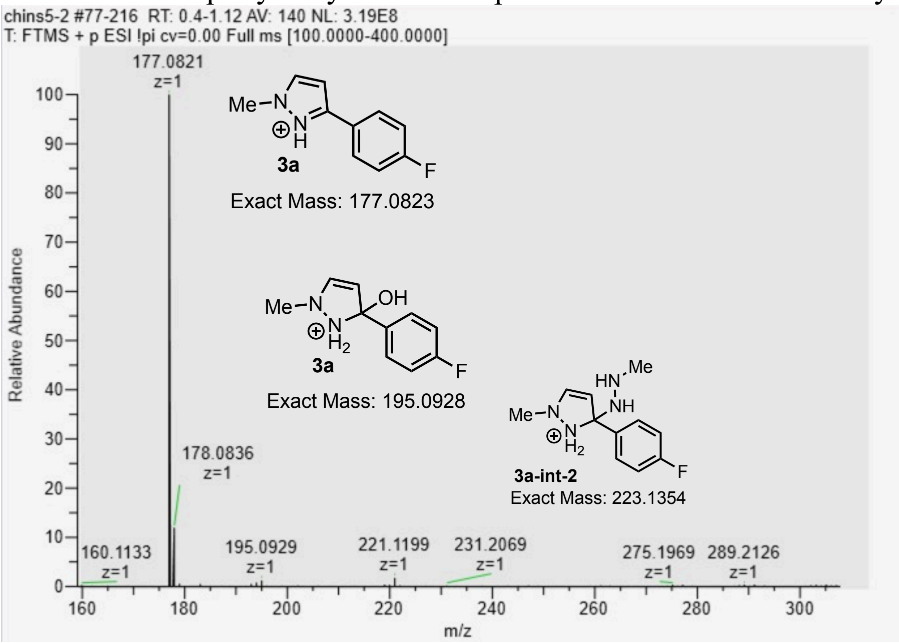

3a Theoretical ESI$^+$: 177.0823, Experimental ESI$^+$: 177.0821

Figure SI-25. Direct-injection mass spectrum of N1 selective reaction mixture with **1a**.

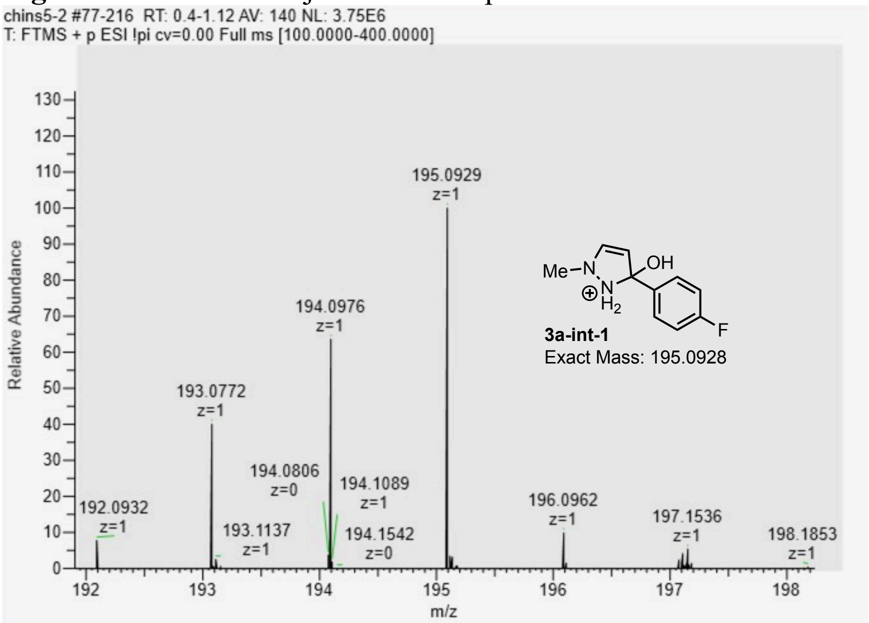

3a-int-1 Theoretical ESI$^+$: 195.0928, Experimental ESI$^+$: 195.0929

Figure SI-26. Direct-injection mass spectrum of N1 selective reaction mixture with **1a** showing **3a-int-1**.

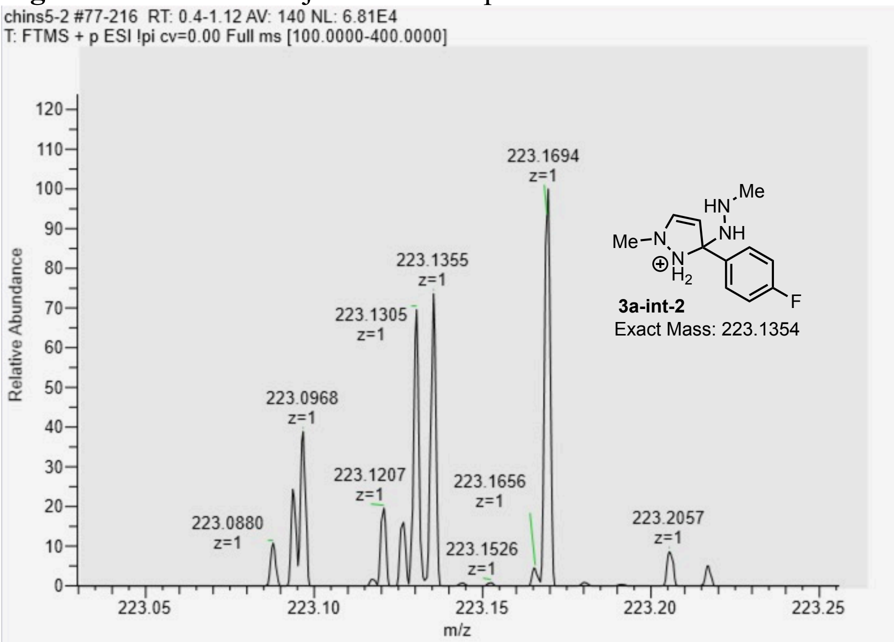

3a-int-2 Theoretical ESI$^+$: 223.1354, Experimental ESI$^+$: 223.1355

Figure SI-27. Direct-injection mass spectrum of N1 selective reaction mixture with **1a** showing **3a-int-2**.

DFT Calculations of 3a and 4a

All calculations were performed using Gaussian 09 (Rev. E.01).[11] Geometry optimizations were performed using the hybrid density functional B3LYP[12] and 6-311G(d,p) basis set. Vibrational frequency calculations were performed for all stationary points to confirm that the optimized structures are at a local minimum. The M06-2X functional[13] with the Def2-TZVP[14] basis set was used for single-point energy calculations in the gas phase and in solution using the SMD continuum solvation model[15] with THF and Et_2O. At equilibrium, **3a**:**4a** is expected to be > 99:1.

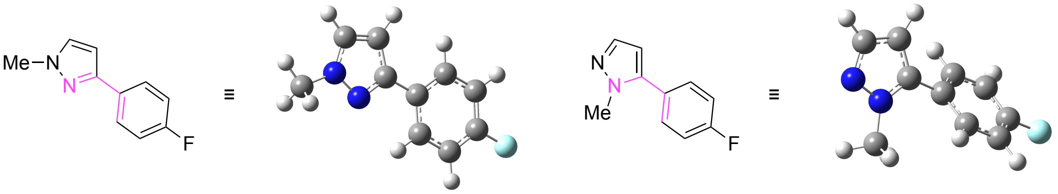

3a
dihedral angle: 0°
0.0 kcal/mol (gas)
0.0 kcal/mol (THF)

4a
dihedral angle: 47°
+1.7 kcal/mol (gas)
+2.0 kcal/mol (THF)

[16]

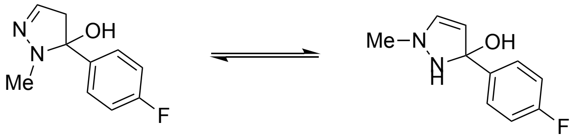

4a-int-N2
0.0 kcal/mol (gas)
0.0 kcal/mol (THF)

4a-int-N1
+11.7 kcal/mol (gas)
+11.3 kcal/mol (THF)

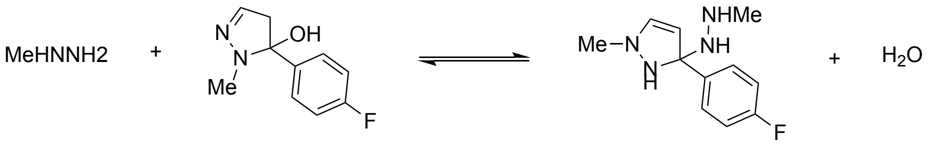

4a-int-N2
0.0 kcal/mol (gas)
0.0 kcal/mol (THF)

4a-int-N1-hyd
+8.7 kcal/mol (gas)
+6.5 kcal/mol (THF)

Figure SI-28. Results of energy minimization DFT calculations of **3a** and **4a**.
==================

---

[11] Gaussian 09, Revision E.01, M. J. Frisch, G. W. Trucks, H. B. Schlegel, G. E. Scuseria, M. A. Robb, J. R. Cheeseman, G. Scalmani, V. Barone, G. A. Petersson, H. Nakatsuji, X. Li, M. Caricato, A. Marenich, J. Bloino, B. G. Janesko, R. Gomperts, B. Mennucci, H. P. Hratchian, J. V. Ortiz, A. F. Izmaylov, J. L. Sonnenberg, D. Williams-Young, F. Ding, F. Lipparini, F. Egidi, J. Goings, B. Peng, A. Petrone, T. Henderson, D. Ranasinghe, V. G. Zakrzewski, J. Gao, N. Rega, G. Zheng, W. Liang, M. Hada, M. Ehara, K. Toyota, R. Fukuda, J. Hasegawa, M. Ishida, T. Nakajima, Y. Honda, O. Kitao, H. Nakai, T. Vreven, K. Throssell, J. A. Montgomery, Jr., J. E. Peralta, F. Ogliaro, M. Bearpark, J. J. Heyd, E. Brothers, K. N. Kudin, V. N. Staroverov, T. Keith, R. Kobayashi, J. Normand, K. Raghavachari, A. Rendell, J. C. Burant, S. S. Iyengar, J. Tomasi, M. Cossi, J. M. Millam, M. Klene, C. Adamo, R. Cammi, J. W. Ochterski, R. L. Martin, K. Morokuma, O. Farkas, J. B. Foresman, and D. J. Fox, Gaussian, Inc., Wallingford CT, 2016.
[12] A. D. Becke, *Phys. Rev. A* **1988**, *38*, 3098–3100; A. D. Becke, *J. Chem. Phys.* **1993**, *98*, 5648–5652; C. Lee, W. Yang, R. G. Parr, *Phys. Rev. B* **1988**, *37*, 785–789
[13] Y. Zhao, D. G. Truhlar, *Theoretical Chemistry Accounts* **2008**, *120*, 215–241.
[14] F. Weigend, R. Ahlrichs, *Phys. Chem. Chem. Phys.* **2005**, *7*, 3297–3305; F. Weigend, *Phys. Chem. Chem. Phys.* **2006**, *8*, 1057–1065
[15] A. V. Marenich, C. J. Cramer, D. G. Truhlar, *J. Phys. Chem. B* **2009**, *113*, 6378–6396
[16] CYLview, 1.0b; Legault, C. Y., Université de Sherbrooke, 2009 (http://www.cylview.org)

3a

===================

C	1.02711000	0.31974300	-0.00003200
C	1.78857700	1.51738400	0.00009200
C	3.10164200	1.10277800	-0.00016400
N	3.08605500	-0.25495100	-0.00032400
H	1.43665300	2.53554100	0.00015500
H	4.02578200	1.65907600	-0.00021500
C	-0.43490000	0.15352700	-0.00001900
C	-1.29222600	1.26170900	0.00037100
C	-1.00523500	-1.12866800	-0.00038700
C	-2.67470300	1.10382300	0.00039600
H	-0.88241800	2.26467700	0.00068000
C	-2.38365300	-1.30109100	-0.00037100
H	-0.35031700	-1.99039200	-0.00069000
C	-3.19904000	-0.17871600	0.00002200
H	-3.34332200	1.95573800	0.00070100
H	-2.83136700	-2.28730900	-0.00065400
N	1.83425300	-0.74640900	-0.00015200
C	4.22118800	-1.16120500	0.00037100
H	4.83729300	-1.00997400	-0.88908000
H	4.83237100	-1.01521300	0.89411900
H	3.82444500	-2.17357500	-0.00366800
F	-4.54264800	-0.34052800	0.00003400

===================

4a

===================

C	1.27295700	-0.36159400	0.10847800
C	2.02864700	-1.47758300	0.44244200
C	3.36598400	-1.06173500	0.32443300
N	3.45285900	0.20762300	-0.06041900
H	1.65202300	-2.43795400	0.75455800
H	4.26810400	-1.62803400	0.50173300
C	-0.18945700	-0.20586100	0.06434900
C	-0.97465100	-1.20388400	-0.53331500
C	-0.84055400	0.89510100	0.64107400
C	-2.36155100	-1.10968400	-0.55996000
H	-0.48806500	-2.05584600	-0.99258900
C	-2.22707800	1.00643500	0.61391600
H	-0.26215500	1.66228600	1.14108700
C	-2.96577000	-0.00053300	0.01232500
H	-2.97363300	-1.87364400	-1.02287100
H	-2.73738600	1.84988900	1.06191700
N	2.17622700	0.62515300	-0.18830200
F	-4.31239400	0.10098200	-0.01537200
C	1.93851500	1.98209300	-0.65277400
H	1.00443500	2.02526500	-1.21151300
H	1.89395500	2.68676700	0.18233400
H	2.76841800	2.25647100	-1.30106900

===================

4a-int-N1

===================

C	-2.24588200	1.41149700	-0.04682700
C	-3.21382200	0.41922600	-0.05590500
C	-2.88457300	-0.92524700	0.00541900
C	-1.54072700	-1.28340400	0.07724300

C	-0.53918800	-0.30803100	0.08455300
C	-0.90678500	1.03861700	0.02132000
H	-2.54508500	2.45150300	-0.09007300
H	-3.67113100	-1.66973200	0.00307400
H	-1.26148900	-2.32577400	0.14402600
H	-0.13326300	1.79475700	0.04200000
F	-4.51835300	0.77459500	-0.12301000
C	0.93334000	-0.70562100	0.10419000
C	1.62667300	-0.74211400	-1.23749400
C	2.83097000	-0.17160500	-1.10536600
H	3.61207900	-0.06536700	-1.84576400
N	1.75635500	0.29584200	0.82000200
N	3.05910000	0.32482000	0.17558500
O	0.99550200	-1.99546800	0.74717500
H	1.82635500	-2.40006000	0.47273300
H	1.88619700	-0.03429300	1.77349600
C	3.68504400	1.64088900	0.28538500
H	4.63581400	1.61874000	-0.25150200
H	3.04794600	2.43760300	-0.12042700
H	3.89402400	1.85517300	1.33447800
H	1.23121700	-1.26004100	-2.09658200

==================

4a-int-N2

==================

C	-2.25823200	1.17663100	-0.53574700
C	-3.09805200	0.16046100	-0.10530500
C	-2.60539400	-1.04107000	0.37684400
C	-1.22620500	-1.23068500	0.42475600
C	-0.35274500	-0.22957000	-0.00690200
C	-0.88275700	0.97401600	-0.48294500
H	-2.68240500	2.10505400	-0.89742600
H	-3.29458000	-1.80726100	0.70962100
H	-0.82067400	-2.15747100	0.80620300
H	-0.20978400	1.76115200	-0.80000100
F	-4.43606100	0.35115900	-0.15352100
C	1.15058200	-0.45265000	-0.00357200
C	1.76818800	-0.76499800	-1.39237400
H	1.17766700	-0.31528300	-2.19740700
C	3.09981600	-0.07190600	-1.26368900
H	3.94701300	-0.17424200	-1.92955500
N	1.89997500	0.77288100	0.34386000
N	3.13605600	0.76209000	-0.29175100
C	1.93944800	1.20682500	1.73348900
H	0.91756200	1.35491900	2.08541000
H	2.43578600	0.48603800	2.39415200
H	2.47148300	2.15654300	1.77694300
H	1.81371900	-1.84096100	-1.57161900
O	1.41893500	-1.51368500	0.91490400
H	2.35716500	-1.72655100	0.85403500

==================

4a-int-N1-hyd

==================

C	-2.49370000	-1.41075100	0.83783800
C	-3.31123500	-0.71183000	-0.03537300

C	-2.81309600	0.27321300	-0.87323300
C	-1.45339200	0.56800600	-0.82566300
C	-0.59893100	-0.11342900	0.04478900
C	-1.13521200	-1.10646600	0.86683800
H	-2.91997200	-2.17612000	1.47466600
H	-3.48529500	0.79531500	-1.54317400
H	-1.04492200	1.33584200	-1.46886200
H	-0.48124400	-1.65597400	1.53349200
F	-4.63433900	-1.00060600	-0.07212400
C	0.90607900	0.15352200	0.04356400
C	1.64952400	-0.21590700	1.31460400
C	2.76725200	-0.86877500	0.97908500
H	3.56053200	-1.22355500	1.62342300
N	1.58011400	-0.73665900	-0.93847200
N	2.89289400	-1.07315000	-0.39636400
H	1.72413600	-0.21659000	-1.80182100
C	3.30807700	-2.40927100	-0.82536000
H	4.26025300	-2.64839500	-0.34654300
H	2.55917400	-3.17048500	-0.57236500
H	3.46230500	-2.41043600	-1.90551500
H	1.36746400	0.12621500	2.29801500
N	1.17017300	1.55979600	-0.37438400
H	2.18170300	1.64503400	-0.49497000
N	0.69831100	2.50552400	0.58230000
H	1.36666600	2.60624500	1.34407700
C	0.45170400	3.80293500	-0.03370300
H	0.14267000	4.50311600	0.74723200
H	1.32538800	4.22641600	-0.55566600
H	-0.36267000	3.71277300	-0.75476300

==================

4a-int-N2-hyd

==================

C	2.72784600	-1.26035600	-0.10828300
C	3.35618200	-0.02672800	-0.04703300
C	2.63590700	1.15542000	-0.01644300
C	1.24397800	1.09971000	-0.04605100
C	0.57471800	-0.12557800	-0.10865900
C	1.33712700	-1.29985400	-0.14024200
H	3.32192200	-2.16577500	-0.12371200
H	3.16229500	2.10089900	0.02956000
H	0.67512400	2.01693600	-0.04722600
H	0.83132300	-2.25694900	-0.17134300
F	4.70844600	0.02297900	-0.01482000
C	-0.94894200	-0.22644200	-0.17243100
C	-1.48915100	-0.58709000	-1.58728500
H	-0.74452400	-1.15286900	-2.15870600
C	-2.64938700	-1.47677800	-1.21771100
H	-3.43640300	-1.82027000	-1.87694100
N	-1.43646300	-1.40383100	0.58347200
N	-2.58242700	-1.90815200	-0.01313100
C	-1.43931400	-1.40622200	2.03902400
H	-0.43980000	-1.14675000	2.38966600
H	-2.15627000	-0.70462300	2.48331900
H	-1.68254600	-2.41479000	2.37390900
H	-1.73734900	0.29689100	-2.17553700
N	-1.62480300	0.95777200	0.41400200

H	-2.57701500	0.67938400	0.65088700
N	-1.63563700	2.12027300	-0.41093800
H	-2.39154900	2.07320300	-1.09272300
C	-1.80516300	3.32064000	0.40516000
H	-0.94339200	3.43066900	1.06570900
H	-1.85424200	4.18818100	-0.25845000
H	-2.71106800	3.30629000	1.03085700

===================

MeHNNH$_2$

===================

N	1.18808200	-0.26972600	0.15909600
H	1.99054500	0.26070900	-0.18001600
H	1.14003800	-1.05453900	-0.49153600
N	0.02322100	0.56898900	-0.19596700
H	0.03214200	1.29125700	0.52072700
C	-1.18541900	-0.22023400	0.03267700
H	-2.05327800	0.43777400	-0.05138200
H	-1.27485100	-0.98432500	-0.74678900
H	-1.20120500	-0.72431900	1.01102900

===================

H$_2$O

===================

O	0.00000000	0.00000000	0.11866500
H	0.00000000	0.75694400	-0.47466100
H	0.00000000	-0.75694400	-0.47466100

HPLC Determination of Product ratio

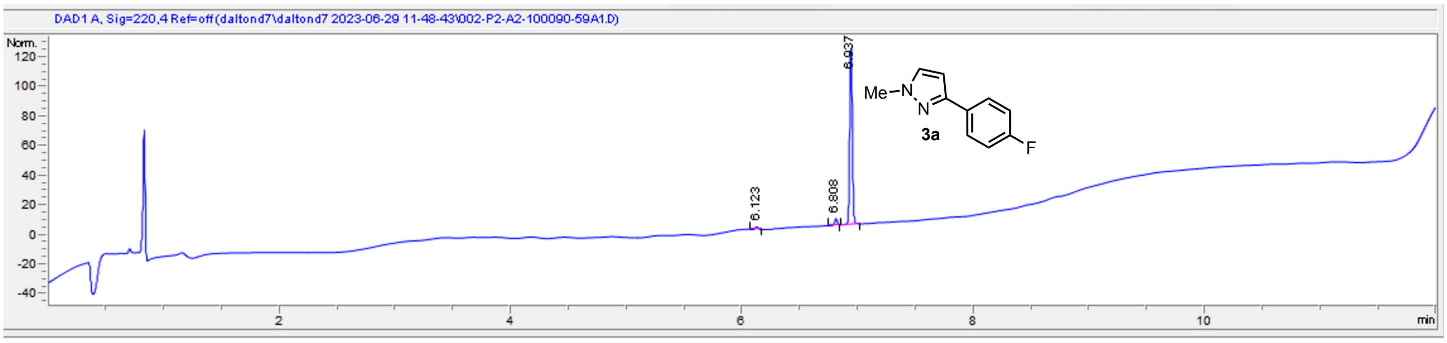

#	Time	Type	Area	Height	Width	Area%	Symmetry
1	6.123	BB	3	1.7	0.0268	1.659	0.945
2	6.808	BB	6.2	4	0.0242	3.492	0.962
3	6.937	BB	169.5	107.4	0.0244	94.849	0.925

Figure SI-29. HPLC of crude N1 selective reaction conditions with vinylogous amide **1a**.

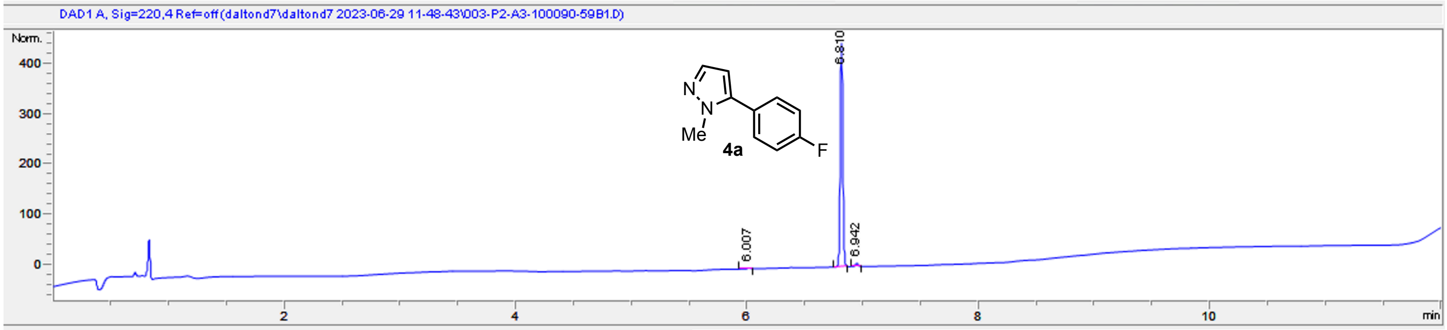

#	Time	Type	Area	Height	Width	Area%	Symmetry
1	6.007	BB	2.9	1.6	0.0281	0.458	1.067
2	6.81	BB	627.5	400.6	0.0243	97.997	0.92
3	6.942	BB	9.9	6.3	0.0252	1.546	0.937

Figure SI-30. HPLC of crude N2 selective reaction conditions with vinylogous amide **1a** (4-F-Ph).

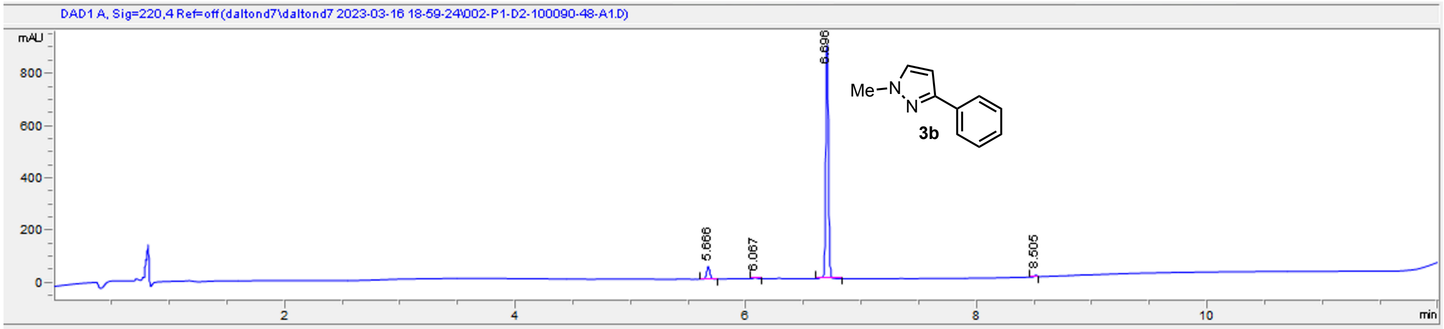

#	Time	Type	Area	Height	Width	Area%	Symmetry
1	5.666	BB	92.8	47.7	0.0307	5.904	0.966
2	6.067	BB	8	4.1	0.0299	0.511	0.728
3	6.696	BB	1460.8	896.3	0.025	92.941	0.934
4	8.505	BB	10.1	6.6	0.0239	0.644	1.07

Figure SI-31. HPLC of crude N1 selective reaction conditions with vinylogous amide **1b**. (Ph)

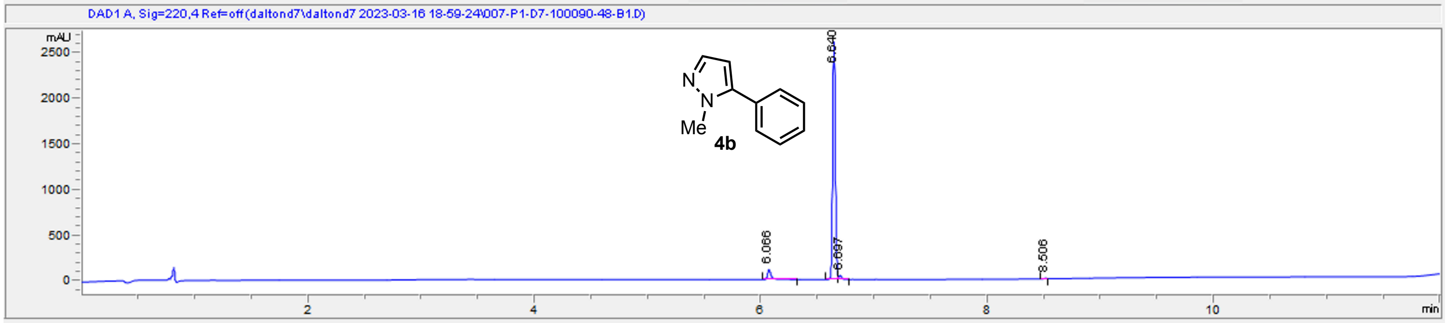

#	Time	Type	Area	Height	Width	Area%	Symmetry
1	6.066	BB	239.3	106.9	0.0331	5.289	0.605
2	6.64	BV	4203.4	2588.8	0.0249	92.930	0.923
3	6.697	VB	68	42.4	0.0237	1.504	0.73
4	8.506	BB	12.5	8.2	0.0239	0.277	1.04

Figure SI-32. HPLC of crude N2 selective reaction conditions with vinylogous amide **1b** (Ph).

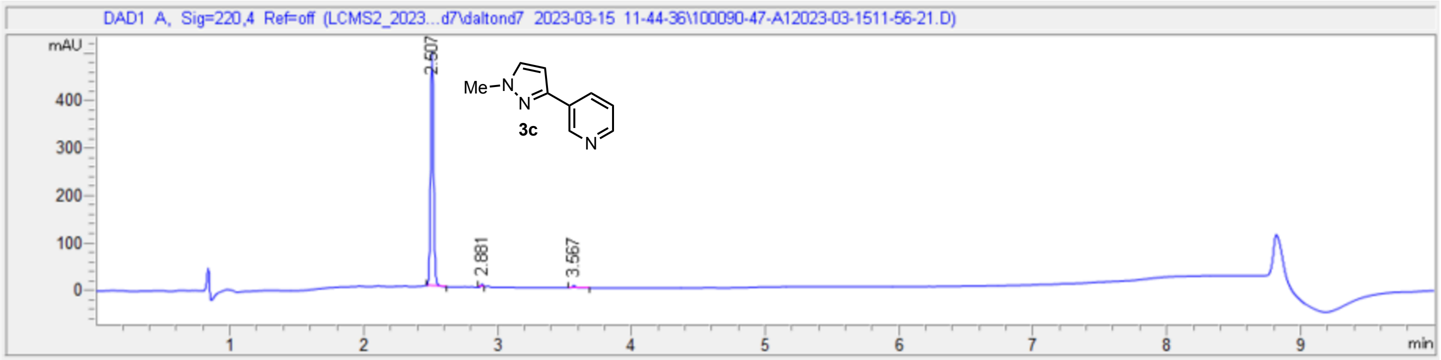

#	Time	Type	Area	Height	Width	Area%	Symmetry
1	2.507	BB	761.6	497.1	0.0234	98.120	0.934
2	2.881	BB	7.4	6.5	0.018	0.960	0.988
3	3.567	BB	7.1	4.9	0.0221	0.921	0.795

Figure SI-33. HPLC of crude N1 selective reaction conditions with vinylogous amide **1c** (pyr)

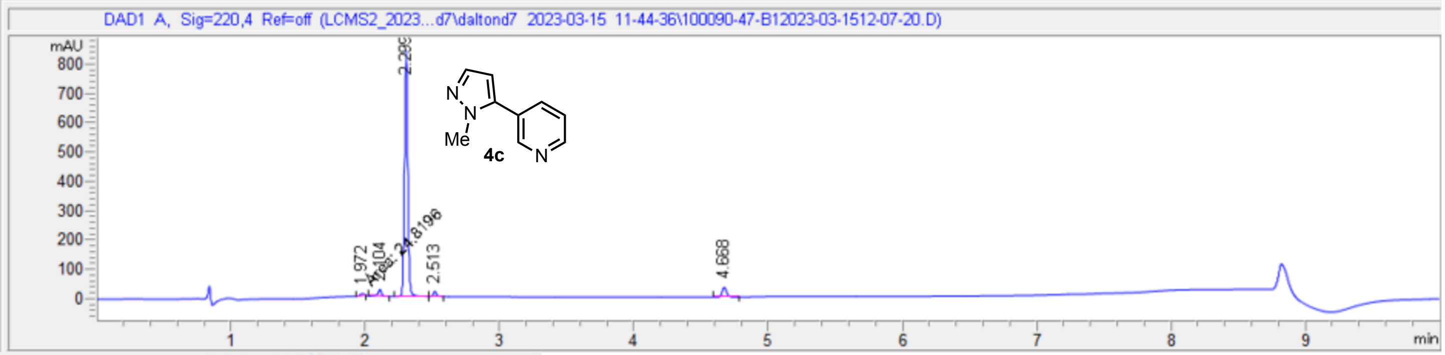

#	Time	Type	Area	Height	Width	Area%	Symmetry
1	1.972	MM	24.8	9.7	0.0426	1.538	1.133
2	2.104	BB	41.8	23	0.0267	2.592	1.417
3	2.299	BB	1444.2	836.6	0.0266	89.513	1.003
4	2.513	BB	27.3	17.2	0.0245	1.691	0.94
5	4.668	BB	75.3	32.8	0.0358	4.665	0.841

Figure SI-34. HPLC of crude N2 selective reaction conditions with vinylogous amide **1c** (pyr).

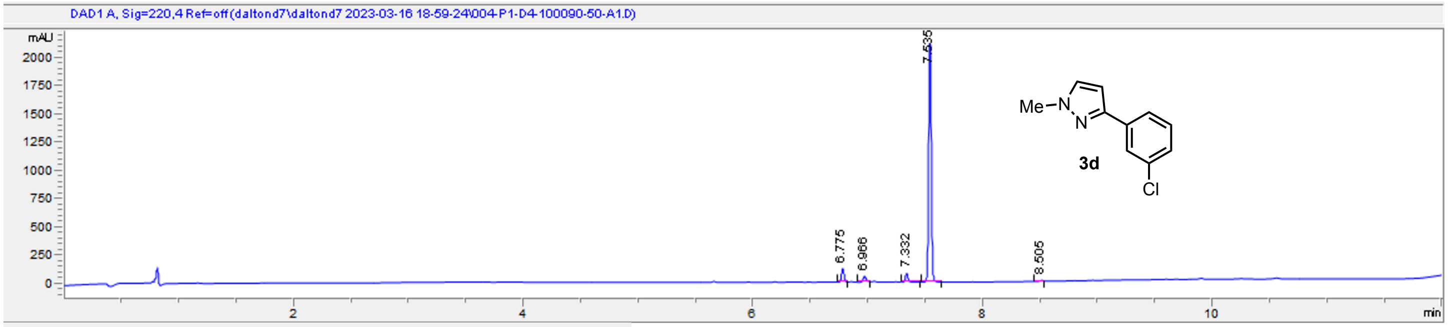

#	Time	Type	Area	Height	Width	Area%	Symmetry
1	6.775	VV	191.9	118	0.026	5.113	0.917
2	6.966	BV	102	51	0.0303	2.718	0.743
3	7.332	VB	119.5	77.2	0.0251	3.184	0.924
4	7.535	BB	3322	2107.3	0.0254	88.517	0.936
5	8.505	VB	17.6	9.7	0.0281	0.468	1.128

Figure SI-35. HPLC of crude N1 selective reaction conditions with vinylogous amide **1d** (3-Cl-ph)

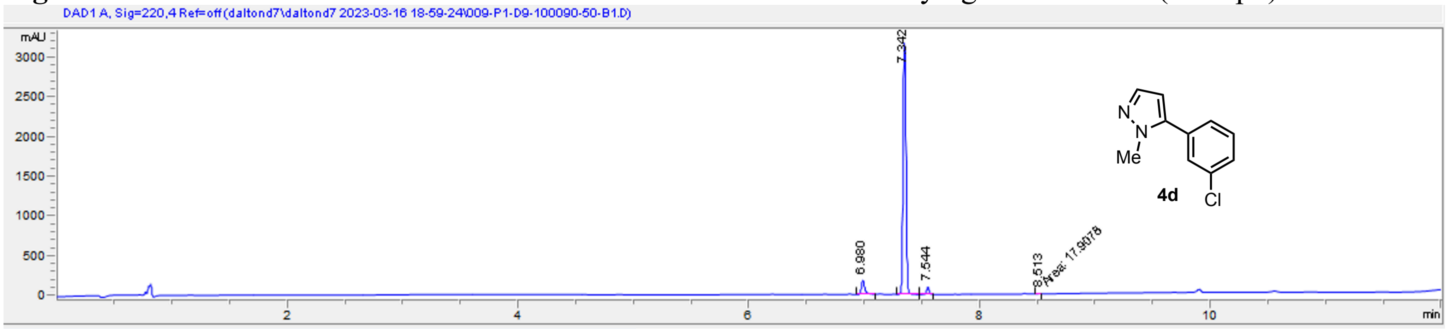

#	Time	Type	Area	Height	Width	Area%	Symmetry
1	6.98	BV	371.5	178.3	0.0313	6.024	0.629
2	7.342	BB	5640.6	3155.1	0.0289	91.461	0.911
3	7.544	BB	137.2	88.8	0.024	2.224	0.976
4	8.513	MM	17.9	9.6	0.0312	0.290	1.014

Figure SI-36. HPLC of crude N2 selective reaction conditions with vinylogous amide **1d** (3-Cl-ph).

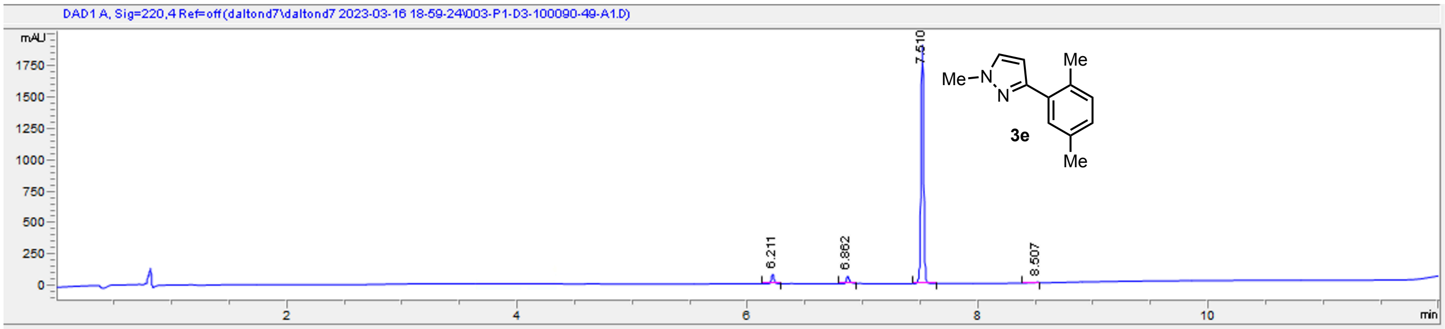

#	Time	Type	Area	Height	Width	Area%	Symmetry
1	6.211	BB	128.9	76.6	0.0256	3.886	0.944
2	6.862	BV	120	58.4	0.031	3.620	0.637
3	7.51	BB	3054.7	1902.9	0.0247	92.127	0.98
4	8.507	BB	12.2	7.2	0.0258	0.367	1.248

Figure SI-37. HPLC of crude N1 selective reaction conditions with vinylogous amide **1e** (1,4-dimethyl-Ph)

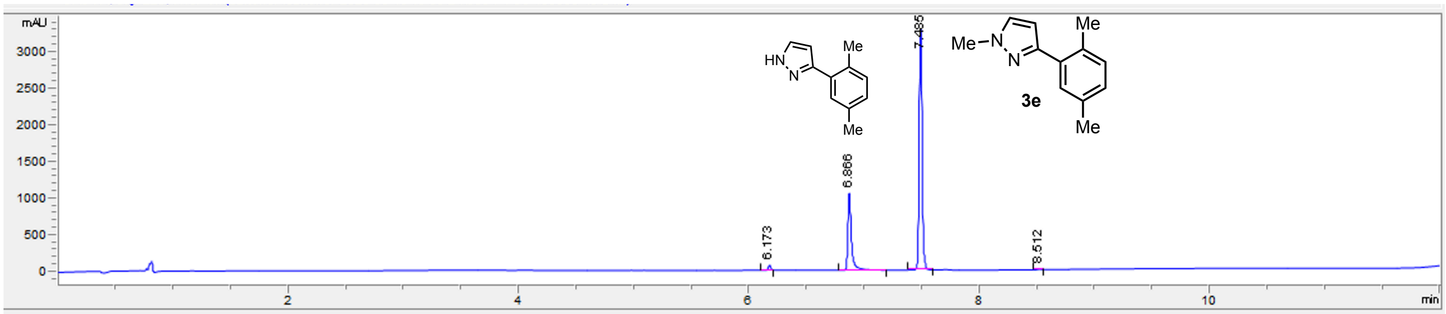

#	Time	Type	Area	Height	Width	Area%	Symmetry
1	6.173	BV	107	69.5	0.024	1.296	0.911
2	6.866	BB	2234.9	1047	0.0319	27.079	0.581
3	7.485	BB	5896.9	3291.3	0.0279	71.452	0.88
4	8.512	BB	14.2	9	0.0254	0.172	0.968

Figure SI-38. HPLC of crude N2 selective reaction with vinylogous amide **1e** run at 12.5 °C.

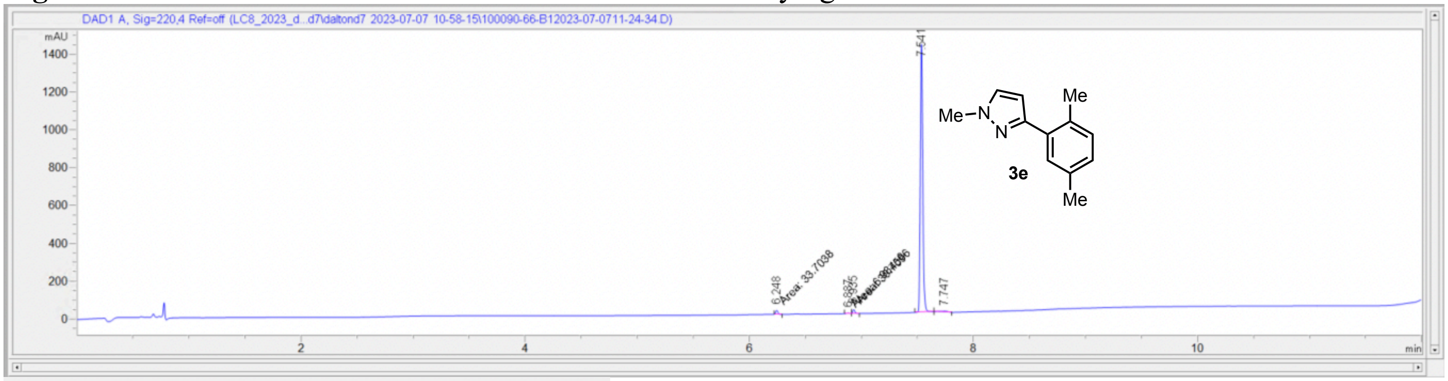

#	Time	Type	Area	Height	Width	Area%	Symmetry
1	6.248	MM	33.7	21.9	0.0256	1.501	0.9
2	6.887	MM	7	3	0.0383	0.311	1.232
3	6.935	MM	30.7	20.8	0.0246	1.368	0.791
4	7.541	BB	2154.2	1418.7	0.0237	95.935	0.84
5	7.747	BB	19.9	3.9	0.0794	0.885	1.274

**run at 0 °C.

Figure SI-39. HPLC of crude N2 selective reaction conditions with vinylogous amide **1e** (1,4-dimethyl-Ph).

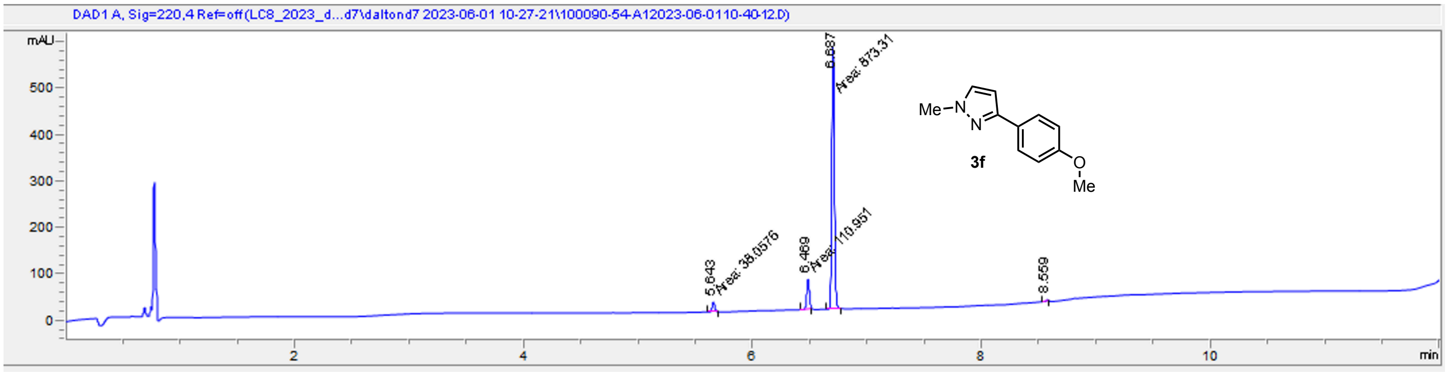

#	Time	Type	Area	Height	Width	Area%	Symmetry
1	5.643	MM	38.1	21.6	0.0293	3.698	0.905
2	6.469	MM	111	64.8	0.0285	10.781	0.951
3	6.687	MM	873.3	566.8	0.0257	84.856	0.887
4	8.559	VB	6.8	4.4	0.0241	0.666	1.147

Figure SI-40. HPLC of crude N1 selective reaction conditions with vinylogous amide **1f** (4-OMe-ph)

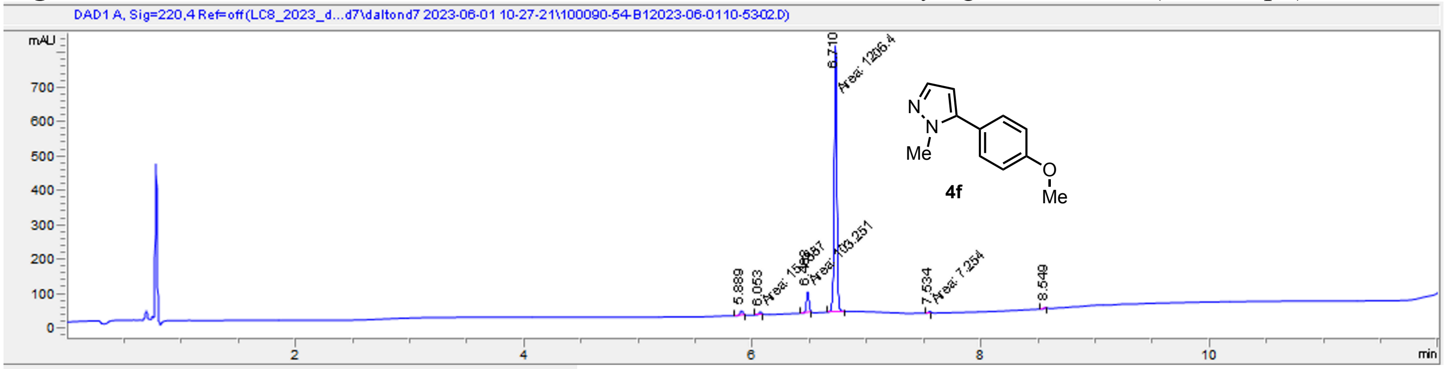

#	Time	Type	Area	Height	Width	Area%	Symmetry
1	5.889	BV	27.2	14.2	0.0298	1.990	0.976
2	6.053	MM	15.8	9.3	0.0283	1.156	0.892
3	6.468	MM	103.3	60.6	0.0284	7.563	0.938
4	6.71	MM	1206.4	771.1	0.0261	88.364	0.986
5	7.534	MM	7.3	5.5	0.0221	0.531	0.888
6	8.549	BB	5.4	3.9	0.0222	0.395	1.046

Figure SI-41. HPLC of crude N2 selective reaction conditions with vinylogous amide **1f** (4-OMe-ph).

Single Crystal X-ray Analysis of 3a

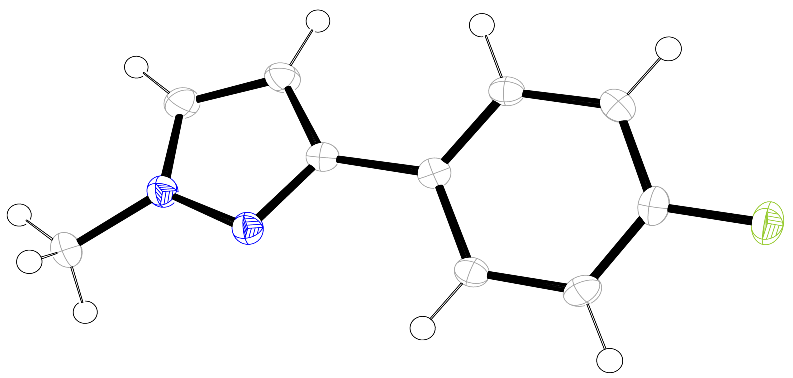

Table SI-12. Crystal data and structure refinement for N-methyl-4-F-phenyl pyrazole **3a** (100090-51-2a).

Crystals suitable for x-ray analysis were grown by slow diffusion of n-heptane into a solution of *N*-methyl pyrazole **3a** (0.025 mg) in DCM (0.5 mL) to obtain clear colorless crystals.

X-ray quality crystals were used as received. A colorless prism 0.160 x 0.160 x 0.040 mm^3 in size was mounted on a Cryoloop with Paratone oil. Single Crystal X-ray crystallography data was collected with a Rigaku Synergy Custom SC-XRD with HyPix 6000 Hybrid Pixel Detector. Data was collected in a nitrogen gas stream at 90(2) K using phi and omega scans. Crystal-to-detector distance was 40 mm and exposure time was 0.05 seconds per frame using a scan width of 0.5°. Data collection was 100.0% complete to 67.000° in q. A total of 16536 reflections were collected covering the indices, -6<=h<=7, -8<=k<=9, -25<=l<=25. 1731 reflections were found to be symmetry independent, with an R_{int} of 0.0319. Indexing and unit cell refinement indicated a primitive, orthorhombic lattice. The space group was found to be P 21 21 21. The data were integrated and scaled using CrysAlisPro 1.171.42.89a. Solution by iterative methods (SHELXT-2018) produced a complete heavy-atom phasing model. All non-hydrogen atoms were refined anisotropically by full-matrix least-squares (SHELXL-2019/2). All hydrogen atoms were placed using a riding model. Their positions were constrained relative to their parent atom using the appropriate HFIX command in SHELXL-2019/2.

Identification code	100090-51-2a	
Empirical formula	C10 H9 F N2	
Formula weight	176.19	
Temperature	90(2) K	
Wavelength	1.54184 Å	
Crystal system	Orthorhombic	
Space group	P 21 21 21	
Unit cell dimensions	a = 5.80000(10) Å	α= 90°.
	b = 7.18730(10) Å	β= 90°.
	c = 20.6605(3) Å	γ = 90°.
Volume	861.26(2) $Å^3$	
Z	4	
Density (calculated)	1.359 Mg/m^3	
Absorption coefficient	0.812 mm^{-1}	
F(000)	368	
Crystal size	0.160 x 0.160 x 0.040 mm^3	
Theta range for data collection	4.280 to 77.207°.	
Index ranges	-6<=h<=7, -8<=k<=9, -25<=l<=25	
Reflections collected	16536	
Independent reflections	1731 [R(int) = 0.0319]	
Completeness to theta = 67.000°	100.0 %	
Absorption correction	Gaussian	
Max. and min. transmission	1.000 and 0.667	
Refinement method	Full-matrix least-squares on F^2	
Data / restraints / parameters	1731 / 0 / 120	
Goodness-of-fit on F^2	1.107	
Final R indices [I>2sigma(I)]	R1 = 0.0254, wR2 = 0.0670	
R indices (all data)	R1 = 0.0259, wR2 = 0.0673	
Absolute structure parameter	0.03(4)	
Extinction coefficient	0.0036(8)	
Largest diff. peak and hole	0.210 and -0.130 e.$Å^{-3}$	

Table SI-13. Atomic coordinates (x 10^4) and equivalent isotropic displacement parameters ($Å^2$x 10^3) for 100090-51-2a. U(eq) is defined as one third of the trace of the orthogonalized U^{ij} tensor.

	x	y	z	U(eq)
C(1)	2126(3)	6849(2)	7294(1)	21(1)
C(2)	1445(3)	6537(2)	6667(1)	21(1)
C(3)	3492(3)	6092(2)	6335(1)	16(1)
C(4)	3800(2)	5546(2)	5652(1)	16(1)
C(5)	2101(2)	5940(2)	5192(1)	17(1)
C(6)	2375(3)	5405(2)	4550(1)	19(1)
C(7)	4363(3)	4477(2)	4379(1)	19(1)
C(8)	6095(2)	4069(2)	4815(1)	19(1)
C(9)	5801(3)	4616(2)	5454(1)	17(1)
C(10)	5941(3)	6746(2)	7877(1)	24(1)
N(1)	5318(2)	6138(2)	6733(1)	18(1)
N(2)	4434(2)	6600(2)	7316(1)	19(1)
F(1)	4621(2)	3914(1)	3754(1)	26(1)

Table SI-14. Bond lengths [Å] and angles [°] for 100090-51-2a.

C(1)-N(2)	1.351(2)	C(6)-H(6)	0.9500
C(1)-C(2)	1.374(2)	C(7)-F(1)	1.3608(17)
C(1)-H(1)	0.9500	C(7)-C(8)	1.380(2)
C(2)-C(3)	1.408(2)	C(8)-C(9)	1.388(2)
C(2)-H(2)	0.9500	C(8)-H(8)	0.9500
C(3)-N(1)	1.3414(19)	C(9)-H(9)	0.9500
C(3)-C(4)	1.474(2)	C(10)-N(2)	1.455(2)
C(4)-C(5)	1.398(2)	C(10)-H(10A)	0.9800
C(4)-C(9)	1.401(2)	C(10)-H(10B)	0.9800
C(5)-C(6)	1.391(2)	C(10)-H(10C)	0.9800
C(5)-H(5)	0.9500	N(1)-N(2)	1.3503(18)
C(6)-C(7)	1.379(2)		
N(2)-C(1)-C(2)	107.13(13)	C(1)-C(2)-C(3)	104.74(13)
N(2)-C(1)-H(1)	126.4	C(1)-C(2)-H(2)	127.6
C(2)-C(1)-H(1)	126.4	C(3)-C(2)-H(2)	127.6

N(1)-C(3)-C(2)	111.18(12)	C(7)-C(8)-H(8)	121.0
N(1)-C(3)-C(4)	119.81(13)	C(9)-C(8)-H(8)	121.0
C(2)-C(3)-C(4)	128.96(14)	C(8)-C(9)-C(4)	121.05(14)
C(5)-C(4)-C(9)	118.77(14)	C(8)-C(9)-H(9)	119.5
C(5)-C(4)-C(3)	120.70(13)	C(4)-C(9)-H(9)	119.5
C(9)-C(4)-C(3)	120.53(13)	N(2)-C(10)-H(10A)	109.5
C(6)-C(5)-C(4)	120.81(14)	N(2)-C(10)-H(10B)	109.5
C(6)-C(5)-H(5)	119.6	H(10A)-C(10)-H(10B)	109.5
C(4)-C(5)-H(5)	119.6	N(2)-C(10)-H(10C)	109.5
C(7)-C(6)-C(5)	118.29(14)	H(10A)-C(10)-H(10C)	109.5
C(7)-C(6)-H(6)	120.9	H(10B)-C(10)-H(10C)	109.5
C(5)-C(6)-H(6)	120.9	C(3)-N(1)-N(2)	104.68(12)
F(1)-C(7)-C(6)	118.63(14)	N(1)-N(2)-C(1)	112.27(13)
F(1)-C(7)-C(8)	118.39(14)	N(1)-N(2)-C(10)	119.99(13)
C(6)-C(7)-C(8)	122.98(14)	C(1)-N(2)-C(10)	127.73(14)
C(7)-C(8)-C(9)	118.09(13)		

Symmetry transformations used to generate equivalent atoms:

Table SI-15. Anisotropic displacement parameters (Å^{2}x 10^3)for 100090-51-2a. The anisotropic displacement factor exponent takes the form: -2π^2[h^{2}a*^{2}U^{11} + ... + 2 h k a* b* U^{12}__

	U^{11}	U^{22}	U^{33}	U^{23}	U^{13}	U__
C(1)	18(1)	27(1)	19(1)	0(1)	4(1)	3(1)
C(2)	16(1)	27(1)	19(1)	2(1)	1(1)	2(1)
C(3)	15(1)	15(1)	18(1)	1(1)	0(1)	-1(1)
C(4)	16(1)	14(1)	17(1)	2(1)	2(1)	-2(1)
C(5)	15(1)	16(1)	21(1)	1(1)	0(1)	0(1)
C(6)	18(1)	19(1)	19(1)	2(1)	-3(1)	-2(1)
C(7)	23(1)	19(1)	16(1)	-2(1)	3(1)	-5(1)
C(8)	16(1)	19(1)	23(1)	-1(1)	4(1)	0(1)
C(9)	14(1)	17(1)	19(1)	1(1)	-1(1)	-1(1)
C(10)	25(1)	28(1)	18(1)	-4(1)	-5(1)	2(1)
N(1)	18(1)	20(1)	16(1)	-1(1)	0(1)	-1(1)
N(2)	19(1)	23(1)	16(1)	-1(1)	0(1)	0(1)
F(1)	29(1)	33(1)	16(1)	-6(1)	2(1)	0(1)

Table SI-16. Hydrogen coordinates (x 10^4) and isotropic displacement parameters (Å^{2}x 10^3) for 100090-51-2__

	x	y	z	U(e__

H(1)	1157	7178	7647	26
H(2)	-71	6607	6495	25
H(5)	743	6581	5320	21
H(6)	1221	5673	4237	23
H(8)	7449	3432	4681	23
H(9)	6975	4355	5761	20
H(10A)	6789	5577	7932	36
H(10B)	5014	6990	8264	36
H(10C)	7035	7768	7811	36

Table SI-17. Torsion angles [°] for 100090-51-2a.

N(2)-C(1)-C(2)-C(3)	0.21(18)
C(1)-C(2)-C(3)-N(1)	-0.34(18)
C(1)-C(2)-C(3)-C(4)	177.07(15)
N(1)-C(3)-C(4)-C(5)	-163.30(14)
C(2)-C(3)-C(4)-C(5)	19.5(2)
N(1)-C(3)-C(4)-C(9)	16.9(2)
C(2)-C(3)-C(4)-C(9)	-160.27(15)
C(9)-C(4)-C(5)-C(6)	0.6(2)
C(3)-C(4)-C(5)-C(6)	-179.15(14)
C(4)-C(5)-C(6)-C(7)	0.0(2)
C(5)-C(6)-C(7)-F(1)	178.59(13)
C(5)-C(6)-C(7)-C(8)	-0.5(2)
F(1)-C(7)-C(8)-C(9)	-178.76(13)
C(6)-C(7)-C(8)-C(9)	0.3(2)
C(7)-C(8)-C(9)-C(4)	0.3(2)
C(5)-C(4)-C(9)-C(8)	-0.8(2)
C(3)-C(4)-C(9)-C(8)	178.97(14)
C(2)-C(3)-N(1)-N(2)	0.32(17)
C(4)-C(3)-N(1)-N(2)	-177.35(13)
C(3)-N(1)-N(2)-C(1)	-0.18(17)
C(3)-N(1)-N(2)-C(10)	178.81(13)
C(2)-C(1)-N(2)-N(1)	-0.03(19)
C(2)-C(1)-N(2)-C(10)	-178.92(15)

Symmetry transformations used to generate equivalent atoms:
